# Targeting an MDM2/MYC Axis to Overcome Drug Resistance in Multiple Myeloma

**DOI:** 10.3390/cancers14061592

**Published:** 2022-03-21

**Authors:** Omar Faruq, Davidson Zhao, Mariusz Shrestha, Andrea Vecchione, Eldad Zacksenhaus, Hong Chang

**Affiliations:** 1 Department of Laboratory Medicine and Pathobiology, University of Toronto, Toronto, ON M5S 1A8, Canada; omar.faruq@uhnresearch.ca (O.F.); davidson.zhao@mail.utoronto.ca (D.Z.); m.shrestha@mail.utoronto.ca (M.S.); eldad.zacksenhaus@uhnresearch.ca (E.Z.); 2 Laboratory Medicine Program, Department of Laboratory Hematology, University Health Network, Toronto, ON M5G 2C4, Canada; 3 Toronto General Hospital Research Institute, University Health Network, Toronto, ON M5G 2C4, Canada; 4 Department of Clinical and Molecular Medicine, Sant’Andrea Hospital, “La Sapienza” University, 00189 Rome, Italy; andrea.vecchione@uniroma1.it

**Keywords:** multiple myeloma, MDM2, c-Myc, MX69, drug resistance

## Abstract

**Simple Summary:**

We show that high expression of MDM2 is associated with poor prognosis and enhanced drug resistance in human myeloma cell lines (HMCLs). Inhibition of MDM2 by RNAi or by the MDM2/XIAP dual inhibitor MX69 significantly increased the sensitivity of resistant HMCLs and primary MM samples to bortezomib and other anti-myeloma drugs, demonstrating that MDM2 can modulate drug response. We uncovered a novel oncogenic regulatory loop between MDM2 and c-Myc that mediated MM drug resistance. MDM2 inhibition resulted in a remarkable induction of apoptosis and suppression of relapsed MM cell growth. Mechanistically, MDM2 stabilized c-Myc mRNA to upregulate its expression, while c-Myc transcriptionally inducedMDM2. Targeting this regulatory loop with MX69 re-sensitized chemo-resistant MM cells to anti-myeloma agents, irrespective of TP53 tumor suppressor status, and prolonged survival in xenograft MM mouse model. These results establish a rationale for therapeutic targeting of the MDM2/c-Myc axis to improve the clinical outcome of patients with refractory/relapsed MM.

**Abstract:**

Background: MDM2 is elevated in multiple myeloma (MM). Although traditionally, MDM2 negatively regulates p53, a growing body of research suggests that MDM2 plays several p53-independent roles in cancer pathogenesis as a regulator of oncogene mRNA stability and translation. Yet, the molecular mechanisms underlying MDM2 overexpression and its role in drug resistance in MM remain undefined. Methods: Both myeloma cell lines and primary MM samples were employed. Cell viability, cell cycle and apoptosis assays, siRNA transfection, quantitative real-time PCR, immunoblotting, co-immunoprecipitation (Co-IP), chromatin immunoprecipitation (ChIP), soft agar colony formation and migration assay, pulse-chase assay, UV cross-linking, gel-shift assay, RNA-protein binding assays, MEME-analysis for discovering c-Myc DNA binding motifs studies, reporter gene constructs procedure, gene transfection and reporter assay, MM xenograft mouse model studies, and statistical analysis were applied in this study. Results: We show that MDM2 is associated with poor prognosis. Importantly, its upregulation in primary MM samples and human myeloma cell lines (HMCLs) drives drug resistance. Inhibition of MDM2 by RNAi, or by the MDM2/XIAP dual inhibitor MX69, significantly enhanced the sensitivity of resistant HMCLs and primary MM samples to bortezomib and other anti-myeloma drugs, demonstrating that MDM2 can modulate drug response. MDM2 inhibition resulted in a remarkable suppression of relapsed MM cell growth, colony formation, migration and induction of apoptosis through p53-dependent and -independent pathways. Mechanistically, MDM2 was found to reciprocally regulate c-Myc in MM; MDM2 binds to AREs on c-Myc 3′UTR to increase c-Myc mRNA stability and translation, while MDM2 is a direct transcriptional target of c-Myc. MDM2 inhibition rendered c-Myc mRNA unstable, and reduced c-Myc protein expression in MM cells. Importantly, in vivo delivery of MX69 in combination with bortezomib led to significant regression of tumors and prolonged survival in an MM xenograft model. Conclusion: Our findings provide a rationale for the therapeutic targeting of MDM2/c-Myc axis to improve clinical outcome of patients with refractory/relapsed MM.

## 1. Introduction

Multiple myeloma (MM) is a plasma cell malignancy characterized by abnormal proliferation of clonal plasma cells in the bone marrow. MM accounts for approximately 1% of all cancers and 10% of hematological malignancies. Although current anti-myeloma agents such as bortezomib and lenalidomide have significantly improved the outcomes of MM patients, MM remains an incurable disease with a high rate of development of drug resistance and relapse. Therefore, the development of novel anti-myeloma agents and studies into the mechanisms of MM drug resistance are greatly needed [1,2,3,4].

Mouse double minute 2 homolog (MDM2) is an E3 ubiquitin ligase that is associated with radio/chemotherapy resistance in several hematological malignancies [5]. Early studies discovered that MDM2 is overexpressed in MM [6], a phenomenon that was shown to enhance myeloma cell cycle progression, proliferation, and survival [6,7]. MDM2 is best characterized for binding and ubiquitinating the tumor suppressor protein p53, targeting it for proteasomal degradation. Previous studies from our group and others have demonstrated the potential of nutlin and RITA as anti-myeloma agents that inhibit MDM2-p53 interaction leading to apoptosis in myeloma cells [8,9,10,11]. Other MDM2 inhibitors such as RG7388, MI77301, AMG232, and HLI98 are actively being investigated in preclinical studies and clinical trials for cancer [11,12]. Therefore, MDM2 is an attractive therapeutic target for MM.

Although TP53 mutations/deletions are rare occurrences (~10%) in newly diagnosed MM [13,14,15,16], TP53 inactivation is an adverse risk factor in MM due to its association with resistance to standard therapy and poor prognosis. Preclinical studies from our group and others have shown that, in the context of TP53 inactivation, therapeutic agents specifically targeting the MDM2/p53 interaction such as nutlin and RITA have limited anti-cancer activity [9]. Therefore, novel drugs effective against TP53-inactivated MM are required.

MDM2 also plays several p53-independent roles in cancer pathogenesis. MDM2 binds the tumor suppressor protein p73 and inhibits its transactivation without promoting its degradation [17]. Moreover, the C-terminal RING domain of MDM2 stabilizes target mRNAs of XIAP, VEGF, MYCN and Slug to regulate gene expression at the translational level [18,19,20,21]. However, the oncogenic pathways regulated by MDM2 in MM have not been fully elucidated.

MX69 is novel MDM2/XIAP dual inhibitor shown to have a robust cytotoxic effect in acute lymphoblastic leukemia and neuroblastoma cells irrespective of p53 status [22]. Mechanistically, MX69 blocks the physical interaction between the MDM2 and XIAP mRNA, thereby inhibiting IRES-dependent translation of XIAP and promoting MDM2 homodimerization, autoubiquitination and self-degradation [22]. However, it remains unknown if MX69 exerts anti-myeloma effects, especially in drug-resistant MM cells. In addition, other potential targets of MX69 have not yet been explored.

In this study, we demonstrate that MDM2 is a potential prognostic biomarker and therapeutic target in MM. We characterize MX69 as a potential anti-myeloma agent with cytotoxic effects in MM cells irrespective of p53 status, both alone and in combination with current frontline anti-myeloma agents. We also uncover a novel oncogenic regulatory mechanism by which MDM2 reciprocally upregulates c-Myc which is inhibited by MX69. Our study provides the preclinical framework for the use of MX69 and other small molecule inhibitors of MDM2 for the treatment of MM.

## 2. Materials and Methods

Cell culture and patient samples: MM1.S, MM1.R, RPMI-8226 (8226S), OPM-2/WT, OPM-2/VR, U266, and 1XG-1 human MM cell lines were obtained from American Type Culture Collection. RPMI-8226R5 (8226R5) is a multidrug-resistant MM cell line that is cross-resistant to bortezomib (BTZ) and was kindly provided by Dr. R Buzzeo [23]. Resistance of RPMI-8226R5 and MM1.R to BTZ were shown in our previous study [24]. Cell culture methods described previously [23,24]. Bone marrow mononuclear cells (BMMNCs) were separated using Ficoll-Hypaque density gradient centrifugation. Primary CD138+ plasma cells were freshly isolated and purified from the bone marrow of MM patients and normal healthy donors. All experiments were approved by the UHN research ethic board.

Drug treatment, cell viability assay, cell cycle and apoptosis assay, quantitative real-time PCR, immunoblotting, co-immunoprecipitation (Co-IP), chromatin immunoprecipitation (ChIP), soft agar colony formation and migration assay, pulse-chase assay, UV cross-linking, gel-shift assay, RNA-protein binding assays, MEME-analysis for discovering c-Myc DNA binding motifs studies, reporter gene constructs procedure, gene transfection and reporter assay, and MM xenograft mouse model studies and statistical analysis were applied in this study.

These assays, studies, and analyses are detailed in Supplementary Methods.

## 3. Results

### 3.1. MDM2 Contributes Chemoresistance in MM

**Figure 1:**
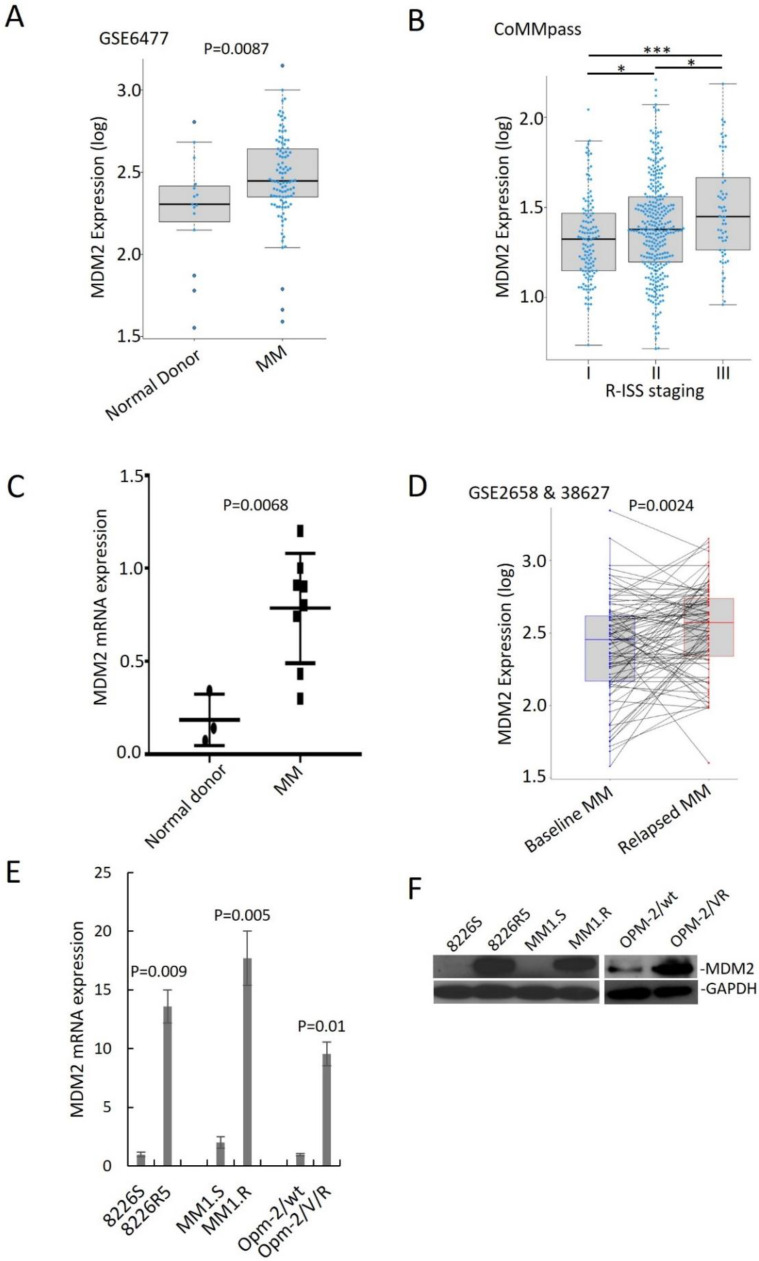
MDM2 overexpression is associated with advanced staging, disease relapse and poor outcomes in MM patients. (**A**) MDM2 expression in normal donor, newly diagnosed MM, and relapsed MM (GSE6477; ND *n* = 15, newly diagnosed MM *n* = 73). (**B**) Baseline MDM2 expression in different clinical stages of MM (CoMMpass; R-ISS-I *n* = 128; R-ISS-II *n* = 318; R-ISS-III *n* = 60). R-ISS-I vs. R-ISS-II, *p* = 0.029; R-ISS-II vs. R-ISS-III, *p* = 0.01, R-ISS-I, vs. R-ISS-III, *p* < 0.0001. (**C**) qRT-PCR quantification of MDM2 mRNA from CD138+ cells of three normal donors and eight MM patients. Results are presented as mean ± SD of three independent experiments. (**D**) MDM2 expression before and after relapse in paired MM samples (GSE2658 expression at diagnosis; GSE38627 expression at relapse; *n* = 88). (**E**) qRT-PCR for MDM2 expression in paired drug-sensitive (8226S, MM1.S and OPM-2/wt) and drug-resistant (8226R5, MM1.R and OPM-2/VR) MM cell lines. Results are presented as mean ± SD of three independent experiments. (**F**) Western blot for MDM2 expression in paired drug-sensitive (8226S, MM1.S and OPM-2/wt) and drug-resistant (8226R5, MM1.R and OPM-2/VR) MM cell lines. The uncropped blot is in Supplemental Figure S12. *, *p* < 0.05; ***, *p* < 0.001.

To assess the clinical outcomes associated with MDM2 upregulation in MM, we analyzed published gene expression profiles of plasma cells from MM patients. MDM2 was significantly upregulated in MM patient samples compared to normal donor samples and in advanced stage of MM diseases (Figure 1A,B). To confirm that MDM2 is upregulated in MM, we quantified RNA from normal donor and MM patient plasma cells isolated by CD138+ selection. We found that MDM2 was at a higher level in MM patient samples compared to normal donor samples (Figure 1C). Moreover, overexpression of MDM2 was correlated with shorter overall survival and progression-free survival in MM patients (Supplemental Figure S1A–D). These findings suggest that overexpression of MDM2 is associated with poor clinical features in MM patients.

Furthermore, we analyzed gene expression profiling (GSE2658 and GSE38627) of 88 paired samples from MM patients at diagnosis and relapse and found that MDM2 was significantly higher in relapsed MM (Figure 1D). We confirmed this finding in an independent dataset (Supplemental Figure S1E). To determine the endogenous MDM2 expression in MM cells, we performed immunoblotting and qRT-PCR on paired drug-sensitive (MM1.S, 8226S and OPM-2/wt) and drug-resistant (MM1.R, 8226R5 and OPM-2/VR) MM cell lines. MDM2 was significantly higher in drug-resistant MM cells compared to their parental drug-sensitive counterparts (Figure 1E,F). These findings suggest that MDM2 contributes to drug resistance in MM.

### 3.2. Silencing MDM2 Induces Apoptosis via p53-Dependent/Independent Pathways in Drug-resistant MM Cells and Re-Sensitizes MM Cells to Conventional Chemotherapy

**Figure 2:**
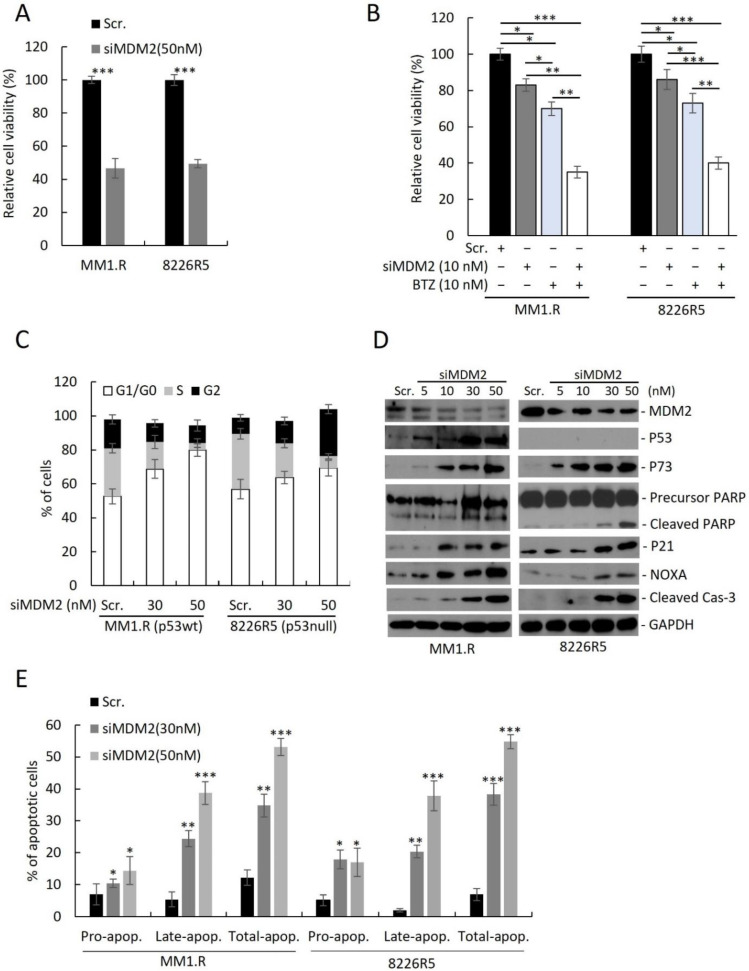
Knockdown of MDM2 induces growth inhibition, apoptosis and cell cycle arrest in drug resistant MM cells. (**A**) Cell viability of MM1.R and 8226R5 cells after 48 h transfection with scrambled siRNA (Scr) or siMDM2 (50 nM). (**B**) Cell viability of MM1.R and 8226R5 cells after 24 h transfection with scrambled siRNA (Scr) or 10 nM siMDM2 followed by 24 h treatment with 10 nM BTZ or drug vehicle. Results are presented as mean ± SD of three independent experiments. (**C**) Bar plots indicate the distribution of cells in each cell cycle phase for MM1.R (p53wt) and 8226R5 (p53null) after 48 h transfection with scrambled siRNA (Scr) or different concentrations of siMDM2, as indicated. Results are presented as mean ± SD of three independent experiments. (**D**) Western blot of cell lysates from MM1.R and 8226R5 cells after 48 h transfection with scrambled siRNA (Scr) or siMDM2. The uncropped blot is in Supplemental Figure S13. (**E**) Apoptosis assay using flow cytometry after staining with annexin V-FITC/propidium iodide. Bar plots indicate the percentage of pro/early apoptotic, late apoptotic and total apoptotic cells for MM1.R and 8226R5 after 48 h transfection with scrambled siRNA (Scr) or different concentrations of siMDM2, as indicated. Results are presented as mean ± SD of three independent experiments. *, *p* < 0.05; **, *p* < 0.01, ***, *p* < 0.001.

To assess the utility of targeting MDM2 in MM, we knocked down (KD) MDM2 in two drug-resistant MM cell lines (MM1.R, p53^wt^; 8226R5, p53^null^) by siRNA and measured cell viability. Transfection with 50 nM of siMDM2 significantly reduced MM1.R and 8226R5 cell viability (Figure 2A) and mRNA of MDM2 (Supplemental Figure S2A). Since MDM2 upregulation was correlated with MM drug resistance, we sought to test if depleting MDM2 could re-sensitize drug resistant MM cells to frontline anti-myeloma agents. The combination of low dose BTZ (10 nM) and siMDM2 (10 nM) significantly impaired MM1.R and 8226R5 cell viability, whereas the exposure to siRNA or drug alone did not significantly impair MM cell viability (Figure 2B). Consistently, MDM2 KD significantly enhanced the cytotoxic effect of Len (5 µM), Dex (10 µM), and Dox (1 µM) in 8226R5 and MM1.R cells (Supplementary Figure S2B). Together, these results indicate that silencing MDM2 inhibits MM cell growth and re-sensitizes drug-resistant MM cells to current anti-myeloma agents irrespective of p53 status.

To determine if MDM2 KD can influence the cell cycle distribution of MM cells, we silenced MDM2 by siRNA in MM cell lines and performed FACS analysis with propidium iodide (PI) staining. MDM2 KD in MM1.R (p53^wt^) cells resulted in an accumulation of G1/G0 phase cells (Figure 2C; Scr. (52.6 ± 1.3%) vs. 30 nM (68.8 ± 2.8%) vs. 50 nM (9.2 ± 2.5%); Supplementary Figure S2C). Conversely MDM2 KD in 8226R5 (p53^null^) resulted in an accumulation of both G1/G0 and G2 phase cells (Figure 2C; Scr. (53.2 ± 2.2%) vs. 30 nM (59.6 ± 3.3%) vs. 50 nM (64.8 ± 3.5%); Supplementary Figure S2D). The cell cycle analysis (Figure 2C) shows that MDM2 depletion results in accumulation of cells in G1 in p53wt and to a lesser extent in p53 null MM cells. Interestingly, while in p53wt MM cells, the increase in G1 was at the expense of reduced S and G2 phases, in p53 null cells, S phase decreased whereas G2 has dramatically increased. These results indicate that MDM2 silencing had differential impact on the cell cycle progression, or on the stage in the cell cycle in which these p53-proficient vs. p53-defcient cells die. However, this distinct cell cycle accumulation did not significantly change the kinetics of cell death as evident from caspase-3 cleavage (Figure 2D), which is observed at 10 uM in both cell lines at similar intensity.

Next, to determine if MDM2 KD activates p53-dependent and independent pathways leading to apoptosis, we measured protein levels of key pro-apoptotic factors. We observed a dose-dependent upregulation of p73, cleaved PARP, p21, NOXA, and cleaved caspase-3 in both MM1.R and 8226R5 cells, as well as p53 in MM1.R cells (Figure 2D). To confirm, that MDM2 KD can induce apoptosis in MM cells, we silenced MDM2 in MM cell lines and performed FACS analysis with PI/annexin V staining. MDM2 KD in MM1.R cells resulted in an increased proportion of apoptotic cells (Figure 2E; Scr. (12.3 ± 3.5%) vs. 30 nM (24.1 ± 3.1%) vs. 50 nM (53.1 ± 1.8%); Supplementary Figure S2E). Similar results were seen after MDM2 KD in 8226R5 cells (Figure 2E, Supplementary Figure S2F). Altogether, these results suggest that depleting MDM2 inhibits MM cell cycle progression and induces apoptosis in MM cells irrespective of p53 status.

### 3.3. MX69 Inhibits the Growth of Drug-Resistant MM Cells through Induction of p53-Dependent and Independent Pathways

**Figure 3:**
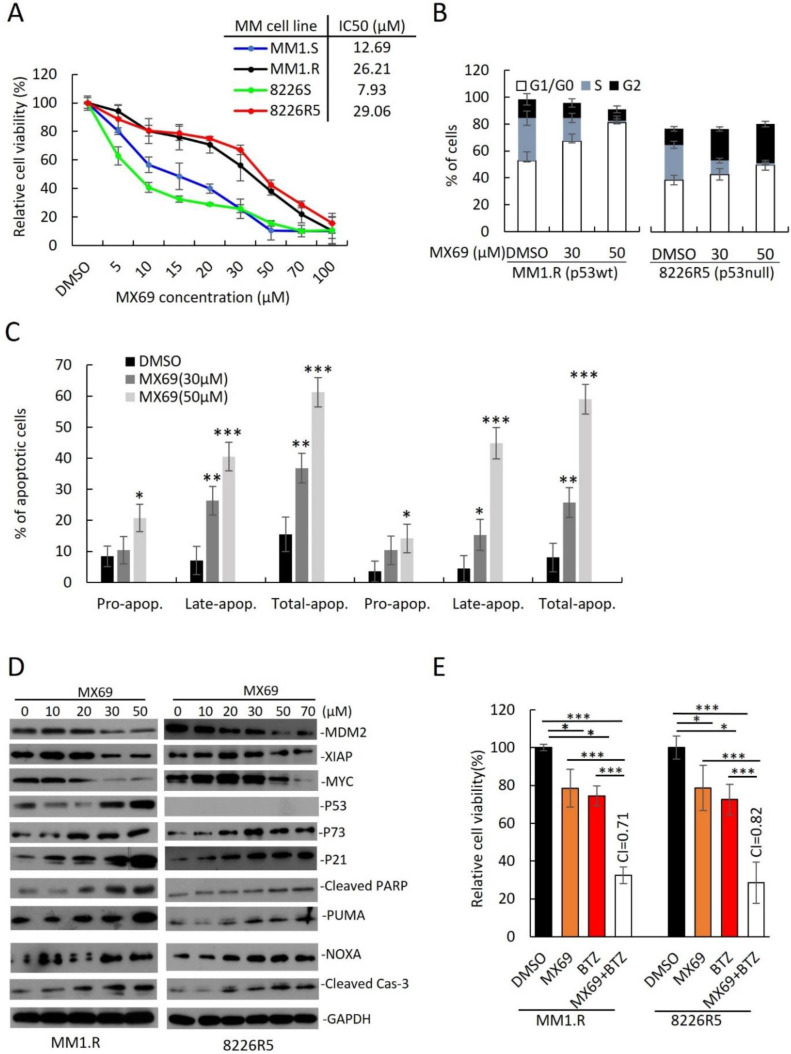
MX69 inhibits MM cell growth, apoptosis and cell cycle arrest in drug-resistant MM cells (**A**) Cell viability of MM cell lines after 48 h treatment with DMSO or different concentrations of MX69, as indicated. Figure insert—IC50 of MX69 in different MM cell lines, as indicated. Results are presented as mean ± SD of three independent experiments. (**B**) Bar plots indicate the distribution of cells in each cell cycle phase for MM1.R (p53wt) and 8226R5 (p53null) after 48 h treatment with DMSO or different concentrations of MX69, as indicated. Results are presented as mean ± SD of three independent experiments. (**C**) Apoptosis assay using flow cytometry after staining with annexin V-FITC/propidium iodide Bar plots indicate the percentage of pro/early apoptotic, late apoptotic, and total apoptotic cells for MM1.R and 8226R5 after 48 h treatment with DMSO or different concentrations of MX69. The distribution of cells in each cell cycle phase indicates MM1.R and 8226R5. Results are presented as mean ± SD of three independent experiments. (**D**) Western blot of cell lysates from MM1.R and 8226R5 cells after 24 h treatment with different concentrations of MX69, as indicated. The uncropped blot is in Supplemental Figure S14. (**E**) Cell viability of MM1.R and 8226R5 after 48 h treatment with DMSO or single/combination treatment with 20 µM MX69 and 5 nM BTZ. Results are presented as mean ± SD of three independent experiments. *, *p* < 0.05; **, *p* < 0.01, ***, *p* < 0.001.

To translate the findings from our MDM2 KD studies to develop novel treatments for MM, we evaluated the anti-myeloma effects of MX69 (Figure 3A, Supplemental Figure S3A). We treated a panel of drug-sensitive and drug-resistant MM cell lines with MX69 and performed cell viability assay. All MM cell lines were sensitive to MX69 regardless of p53 status (Supplementary Figure S3B). While MX69 treatment reduced viability in all MM cell lines, drug-resistant MM cells also showed a hyperbolic dose-response curve and have a two-to-five-fold higher IC50 than their parental drug-sensitive cells, in line with their higher MDM2 basal levels (Figure 3A, Supplementary Figure S3B).

Next, we sought to evaluate whether MX69 can influence the cell cycle distribution of MM cells. MX69 resulted in an accumulation of MM1.R (TP53^wt^) cells in G1/G0 phase (Figure 3B; DMSO (52.8 ± 2.4%) vs. 30 μM (67.2 ± 3.5%) vs. 50 μM (81.2 ± 1.5%); Supplementary Figure S3C). Conversely, MX69 treatment resulted in an accumulation of 8226R5 (p53^null^) cells in G1/G0 and G2 phase (Figure 3B; DMSO (47.8 ± 2.5%) vs. 30 μM (51.5 ± 2.8%) vs. 50 μM (59.4 ± 1.1%); Supplementary Figure S3D). Furthermore, to determine if MX69 treatment can induce apoptosis in MM cells, we performed apoptosis assay. MX69 treatment increased the proportion of apoptotic MM1.R cells (Figure 3C; DMSO (15.4 ± 2.8%) vs. 30 μM (36.03 ± 1.8%) vs. 50 μM (60.4 ± 2.0%); Supplementary Figure S3E). Similar results were seen after treatment with MX69 in 8226R5 cells (Figure 3C; Supplementary Figure S3F). We also observed a dose- and time-dependent upregulation of p21, p73, NOXA, PUMA, cleaved PARP and cleaved Caspase-3 in both MM1.R and 8226R5 cells, as well as p53 in MM1.R cells (Figure 3D, Supplementary Figure S4A). Since MDM2 has been shown to stabilize MYCN mRNA and enhance MYCN translation [18], we hypothesized that MDM2 could also interact similarly with the MYCN homologue c-Myc to induce c-Myc expression. Interestingly, we observed a time- and dose-dependent downregulation of c-Myc protein levels after MX69 treatment (Figure 3D, Supplementary Figure S4A). These results indicate that MX69 inhibits cell cycle progression and induces apoptosis in MM cells irrespective of p53 status.

To confirm the mechanism of action of MX69 as a promoter of MDM2 homodimerization, autoubiquitination and self-degradation, CHX pulse-chase assay confirmed that MX69 promoted MDM2 degradation since the half-life of MDM2 after MX69 treatment (<90 min) was shorter than in DMSO-treated control (>90 min) (Supplemental Figure S4B).

Next, we examined the effects of MX69 on colony-forming and migratory potential of MM cells. Treatment of MM1.R and 8226R5 cells with MX69 significantly inhibited MM colony formation (Supplemental Figure S4C,D) and migration (Supplemental Figure S4E,F). These differences were not due to cytotoxicity as MM cells treated at the indicated doses showed ∼60% viability when cell viability assayed in parallel. These findings suggest that MX69 suppresses the clonogenic and migratory potential of MM cells. Since the standard of care of MM involves combination therapy, we evaluated whether MX69 can synergize with anti-myeloma drugs. Treatment of drug-resistant MM cell lines MM1.R and 8226R5 with suboptimal doses of MX69 (20 µM) or BTZ (5 nM) alone did not significantly decrease MM cell viability, however, combined treatment significantly decreased viability (Figure 3E). Drug synergy by CompuSyn analysis confirmed that MX69 synergized with BTZ with combination index values < 0.9 (Figure 3E). Similarly, single treatment with Len (5 µM), Dex (2.5 µM), and Dox (1 µM) did not significantly reduce MM1.R and 8226R5 cell viability, but there was significant growth inhibition in MM cells when combined with MX69 (Supplemental Figure S5A,B). Drug synergy analysis confirmed that MX69 exerted synergism with Len, Dex, and Dox. These results suggest that MX69 re-sensitizes drug-resistant MM cells to frontline anti-myeloma drugs.

Since MX69 demonstrated synergism with BTZ, we next examined if MX69 treatment could circumvent chemoresistance and enhance the pro-apoptotic effects of BTZ. Combination treatment with MX69 (20 µM) + BTZ (10 nM) resulted in an increase in the proportion of apoptotic MM1.R and 8226R5 cells (Supplemental Figure S5C,D). To confirm that combination treatment with MX69 (20 µM) + BTZ (10 nM) or MX69 (20 µM) + Len (5 µM) activated apoptotic pathways leading to MM cell death, we measured the expression of pro- and anti-apoptotic factors. Immunoblotting revealed that combination treatment with MX69 + BTZ or MX69 + Len downregulated MDM2, XIAP and c-Myc in both MM1.R and 8226R5 cells. We also observed an upregulation in p73, p21, cleaved Caspase-3 and NOXA in both MM1.R and 8226R5 cells, as well as p53 in MM1.R cells (Supplemental Figure S5E). These results indicate that MX69 effectively induces apoptosis in combination with current anti-myeloma agent BTZ [24].

**Figure 4:**
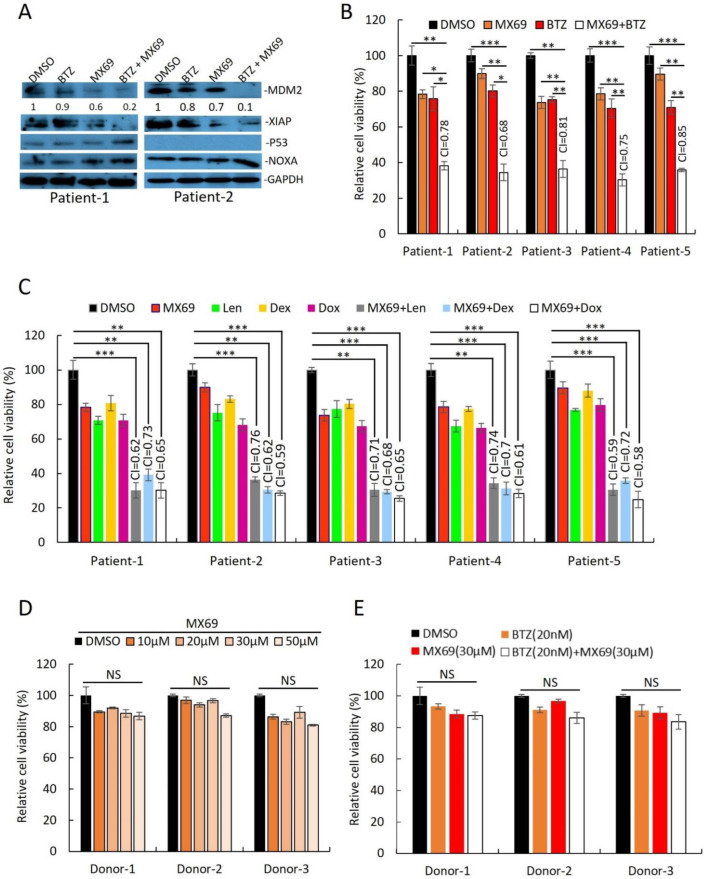
Combination of MX69 with anti-myeloma drugs show synergistic effects on MM patient samples. (**A**) Western blot of cell lysates from MM patient PBMCs after 48 h treatment with DMSO or single/combination treatment with 20 μM MX69 and 10 nM BTZ. MDM2 protein densitometry measured through normalized GAPDH. The uncropped blot is in Supplemental Figure S15. (**B**,**C**) Cell viability of CD138+ cells from MM patients after 48 h treatment with DMSO or single/combination treatment with 20 μM MX69, 10 nM BTZ 5 μM Len, 10 μM Dex, and 1 μM Dox. Results are presented as mean ± SD of three independent experiments. (**D**) Cell viability of normal donor PBMCs after 48 h treatment with DMSO or different concentrations of MX69, as indicated. Results are presented as mean ± SD of three independent experiments. (**E**) Cell viability of normal donor PBMCs after 48 h treatment with DMSO or single/combination treatment with 30 μM MX69 and 20 nM BTZ, as indicated. Results are presented as mean ± SD of three independent experiments. *, *p* < 0.05; **, *p* < 0.01, ***, *p* < 0.001.

Supporting the results from MM cell lines, MX69 had cytotoxic effects in MM patient plasma cells isolated by CD138+ selection. Immunoblotting of cell lysates from two primary MM samples revealed that combination treatment with BTZ (10 nM) + MX69 (20 µM) downregulated MDM2 and XIAP, and upregulated NOXA (Figure 4A). MX69 (20 µM) had synergistic effects when used in combination with BTZ (10 nM), Dex (10 µM), Dox (1 µM), and Len (5 µM) (Figure 4B,C). To rule out off-target cytotoxic effects of MX69, we performed cell viability assay of PBMC obtained from healthy donors following treatment with MX69 and/or BTZ. The cell viability of healthy PBMCs was not significantly impacted by drug concentrations effective against MM cells (Figure 4D,E). Altogether, these results suggest that MX69 is a potential anti-myeloma agent with selective cytotoxic effects in MM cells.

### 3.4. MX69 Inhibits c-Myc mRNA Potentially by Attenuating MDM2-cMYC mRNA Interaction

**Figure 5:**
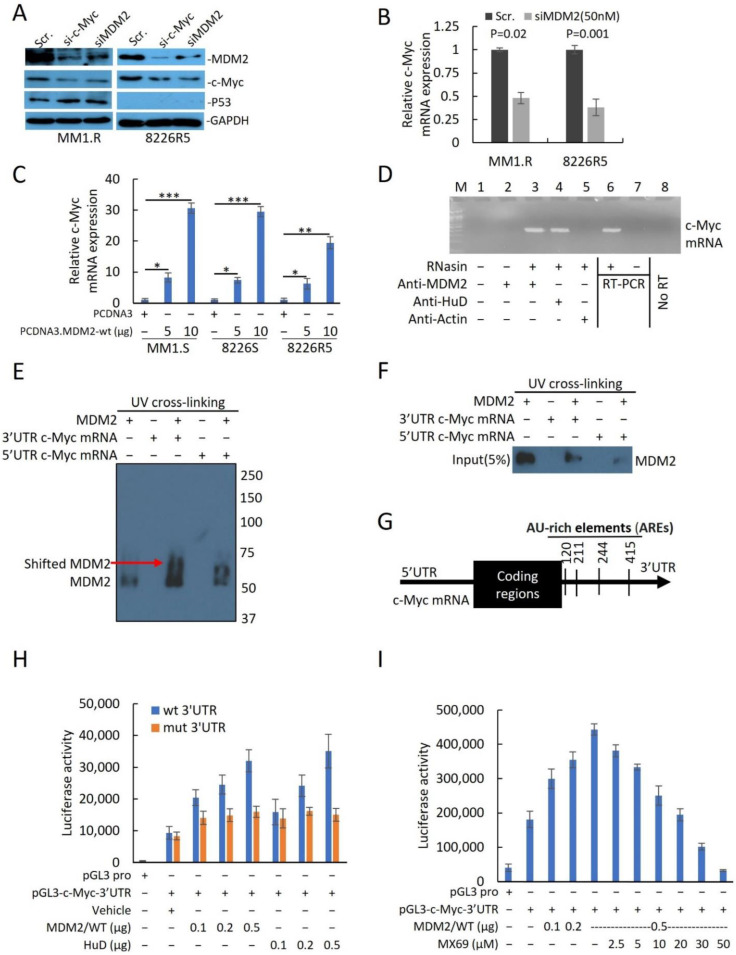
MDM2 directly regulates c-Myc mRNA stabilization and translation in MM cells. (**A**) Western blot of cell lysates from MM1.R and 8226R5 cells after 48 h transfection with si-MDM2 (50 nM) or si-c-Myc (50 nM). The uncropped blot is in Supplemental Figure S16. (**B**) Quantitative RT-PCR indicating the levels of c-Myc expression in 8226R5 cells after 48 h transfection with siMDM2 or control siRNA (Scr) with indicated concentrations. Results are presented as mean ± SD of three independent experiments. (**C**) Quantitative RT-PCR indicating the levels of c-Myc mRNA in MM1.S, 8226S, and 8226R5 cells after transfection with MDM2-WT or control plasmid for 48 h. Results are presented as mean ± SD of three independent experiments. (**D**) Cell extracts prepared from 8226R5 cells in the presence of an RNase inhibitor RNasin. Following Co-IP using anti-MDM2, anti-HuD, or anti-Actin, c-Myc mRNA was detected by traditional RT-PCR analysis. The positive (total RNA from MM1.R as template, lane-6), negative (no template, lane-7) and the no-RT-PCR (lane-8) controls for RT-PCR are also shown. (**E**) Purified MDM2/rhMDM2 was mixed with c-Myc 3′UTR or 5′UTR RNA probes. The protein/RNA complexes were exposed to UV for 10 min for UV cross-linking. Then, samples were loaded onto native–PAGE. Gel protein was detected using immunoblotting. (**F**) Immunoblots of two biotinylated RNA probes (c-Myc 3′UTR and the c-Myc 5′UTR) pull down protein. Pure MDM2/rhMDM2 protein was incubated with biotinylated RNAs immobilized on Streptavidin agarose beads and the bound proteins were eluted and probed with an anti-MDM2 antibody used at a 1:5000 dilution. (**G**) Schematic diagram of c-Myc 3′UTR region marked with AREs. (**H**) 293T and 8226S cells were transfected with 5 µg of pGL3-c-Myc 3′UTR (wt or mutant) plasmids with or without increasing amounts (100, 200, and 500 ng) of MDM2-WT or pcDNA 3.1-HuD plasmids. After 48 h, cell extracts were prepared, and firefly luciferase activity (pGL3-c-Myc 3′UTR) was detected with the Dual-Luciferase Reporter System. The firefly luciferase activities in the transfection of pGL3-c-Myc 3′UTR only were set at 1. Firefly luciferase activity normalized to renila luciferase activity is presented as mean ± SD of three independent experiments. (**I**) Effect of MX69 on MDM2-mediated pGL3-c-Myc 3′UTR activity. 293T cells were co-transfected with 5 µg pGL3-c-Myc 3′UTR, and increasing amounts of MDM2 plasmid (100 ng, 200 ng, and 500 ng) or a constant amount of MDM2 (500 ng), in the presence or absence of increasing amounts of MX69 (2.5, 5, 10, 20, 30, and 50 μM). Controls included transfection of pRL empty vector alone. Quantitative renila luciferase and firefly luciferase activities were detected using the Dual-Luciferase Reporter System. Firefly luciferase activity normalized to renila luciferase activity is presented as mean ± SD of three independent experiments. *, *p* < 0.05; **, *p* < 0.01, ***, *p* < 0.001.

Given that MX69 treatment resulted in the downregulation of c-Myc, we sought to investigate the relationship between MDM2 and c-Myc expression. To confirm the positive association between MDM2 and c-Myc expression levels, we evaluated the effects of MDM2 or c-Myc knockdown in 8226R5 and MM1.R cell lines. Interestingly, we found that knockdown of MDM2 reduced c-Myc protein expression, while knockdown of c-Myc reduced MDM2 protein expression (Figure 5A). Furthermore, knockdown of MDM2 or MX69 treatment resulted in downregulation of c-Myc mRNA levels in 8226R5 and MM1.R cells (Figure 5B, Supplemental Figure S6A–D). We performed qRT-PCR to evaluate the effects of modulating MDM2 expression on c-Myc mRNA levels. Ectopic overexpression of MDM2 resulted in the dose-dependent upregulation of c-Myc mRNA levels in MM1.S, 8226S, and 8226R5 cells (Figure 5C, Supplemental Figure S6E).

To find evidence of the association between MDM2 and c-Myc, we performed correlation analysis of MDM2 in this GSE6477 dataset. MDM2 had a significant correlation with genes (data not shown) in relapsed MM samples, including c-Myc (Supplemental Figure S6F). We confirmed this finding in an independent dataset (Supplemental Figure S6G). To gain insight into the biological significance of the alterations in MDM2 gene expression level, we performed gene set enrichment analysis (GSEA) to identify the specific biological processes, molecular functions, or cellular components which are over-/under-represented in the context of MDM2 overexpression [25]. We stratified the patient sets based on the median MDM2 expression value from GSE6477 resistant MM patient samples. GSEA analysis revealed that MDM2 overexpression phenotype was associated with enrichment of ten hallmark cancer gene sets (Supplemental Figure S6H). Among these ten hallmark gene sets, MYC_TARGETS_V1 and MYC_TARGETS_V2 were enriched in the MDM2 overexpression group (Supplemental Figure S6I,J). These findings suggest that c-Myc and MDM2 expression levels are positively correlated.

Since c-Myc activation contributes to the development of hematological malignancies including MM [26], we investigated the clinical characteristics associated with c-Myc overexpression. Relapsed MM patients’ plasma cells had higher levels of c-Myc compared to newly diagnosed MM, while both groups had higher c-Myc expression compared to normal donor (Supplemental Figure S6K). We further confirmed that relapsed MM patient samples had higher expression levels of c-Myc compared to newly diagnosed MM in an independent dataset (Supplemental Figure S6L). Our findings affirm that c-Myc correlates with MM disease advancement and is a potential therapeutic target in MM.

To further confirm the positive association between MDM2 and c-Myc expression levels, we ectopically overexpressed MDM2 in MM cell lines expressing low endogenous levels of MDM2. Overexpression of MDM2 in drug sensitive (MM1.S and 8226S) cells resulted in upregulated c-Myc expression, which was reversed by MDM2 KD or MX69 treatment (Supplemental Figure S7A,B). In parallel, we performed cell viability assay and found that overexpression of MDM2 resulted in increased viability which was reversed by concurrent siMDM2 transfection or MX69 treatment (Supplemental Figure S7A(below),B(below)). Of note, immunoblotting of p53 in 8226S cells showed MDM2 expression levels had no effect on mutant p53 expression since MDM2 cannot target mutant p53 (Supplemental Figure S7B). Conversely, overexpression of c-Myc in MM1.S and 8226S cells increased MDM2 expression, which was reversed by concurrent si-c-Myc transfection or MX69 treatment (Supplemental Figure S7C,D). In parallel, overexpression of c-Myc resulted in increased MM cell viability, which was reversed by concurrent si-c-Myc transfection or MX69 treatment (Supplemental Figure S7C(below),D(below)). Of note, immunoblotting of 8226S cells showed that c-Myc expression level had no effect on mutant p53 expression (Supplemental Figure S7D). These results confirm that the expression of MDM2 and c-Myc are positively correlated and suggest that MDM2 and c-Myc form a reciprocal regulatory loop.

Next, we sought out to investigate the molecular mechanisms underlying the association between MDM2 and c-Myc expression levels. Since MDM2 stabilizes MYCN mRNA transcript [18] and our results revealed that MDM2 and c-Myc expression levels are correlated, we sought to test whether MDM2 can stabilize c-Myc transcripts. We performed quantitative RT-PCR for after treatment with actinomycin-D to inhibit mRNA synthesis to measure c-Myc mRNA turnover [27]. MDM2 KD significantly reduced the stability of c-Myc mRNA with concurrent treatment with actinomycin-D (Supplemental Figure S8A). This result suggests that MDM2 stabilizes c-Myc mRNA.

To investigate whether MDM2 protein can directly bind to and stabilize c-Myc mRNA, we performed a protein–RNA binding assay to search for possible physical associations between MDM2 protein and c-Myc mRNA. After pulldown of MDM2 or mRNA binding protein like HuD [28] and RT-PCR, revealed that MDM2, like HuD, was able to bind to c-Myc Mrna (Figure 5D). Since MDM2 protein binds either the 3′UTR or 5′UTR mRNA sequence to regulate gene expression at the translational level, we further investigated whether MDM2 binds specifically to the c-Myc 3′UTR or 5′UTR. We performed UV crosslinking of c-Myc 3′UTR or 5′UTR probes with MDM2 protein for gel shift assay. Results indicated that 3′UTR c-Myc mRNA showed greater shift with MDM2 protein compared to 5′UTR c-Myc RNA suggesting that MDM2 binds to the 3′UTR of c-Myc mRNA (Figure 5E). To further confirm that MDM2 binds specifically to the 3′UTR of c-Myc mRNA, we performed UV-crosslinking of UTP-biotinylated c-Myc 3′UTR and 5′UTR RNA probes with 8226R5 cells extracts. RNA-protein complexes that were pulled down revealed that MDM2 binds to the c-Myc 3′UTR RNA probe, but not to the c-Myc 5′UTR RNA probe (Figure 5F). To rule out the possibility that MDM2 binds to c-Myc mRNA through its interaction with endogenous HuD in extracts, we performed co-immunoprecipitation and found no binding interaction between MDM2 and HuD (Supplemental Figure S8B).

To further investigate the binding mechanism between MDM2 and the 3′UTR of c-Myc mRNA, we analyzed the c-Myc 3′UTR sequence and found four AU-rich elements (AREs) (Figure 5G) and hypothesized that MDM2 stabilizes c-Myc mRNA by binding its AREs. To confirm that MDM2 induces c-Myc translation and ascertain whether the translational effect is exerted through the 3′UTR, we constructed a firefly luciferase reporter plasmid containing the c-Myc 3′UTR in a pGL3-promoter (pGL3-c-Myc-3′UTR) and ARE mutations plasmids. Insertion and mutations were confirmed by Sanger Sequencing. Co-transfection of reporter plasmid with MDM2 and HuD (Positive control which binds to 3′UTR AREs) plasmids increased luciferase activity from the c-Myc 3′UTR reporter (Figure 5H). Conversely, cells transfected with ARE mutant plasmid did not show an increased luciferase intensity after co-transfection with MDM2 or HuD plasmid (Figure 5H). Silencing MDM2 reduced the luciferase activity from the c-Myc 3′UTR reporter in MM cells transfected with the pGL3-c-Myc-3′UTR plasmid (Supplemental Figure S8C). Next, we evaluated whether MX69 abrogates MDM2/c-Myc 3′UTR physical interaction to downregulate c-Myc. MX69 reduced luciferase activity from the c-Myc 3′UTR reporter in a dose-dependent manner (Figure 5I). Finally, we sought to rule out other potential mechanisms by which MDM2 regulates c-Myc. We performed a pulse-chase assay to rule out a post-translational mechanism whereby MDM2 regulates c-Myc protein stability. The half-life of the c-Myc protein in siMDM2-transfected cells was comparable to that of control cells (Supplemental Figure S8D). Collectively, these results indicate that MDM2 regulates c-Myc expression through stabilization of the 3′UTR of c-Myc mRNA, and MX69 inhibits MDM2 mediated c-Myc downregulation.

### 3.5. MDM2 Is a Direct Transcriptional Target of c-Myc in MM

Since modulation of c-Myc expression levels resulted in reciprocal change in MDM2 expression levels, we hypothesized that MDM2 is a direct transcriptional target of c-Myc. Bioinformatics studies identified DNase I hypersensitive sites (open chromatic regions of DNA) and high levels of H3K27Ac in the promoter region of MDM2 at predicted c-Myc binding sites, suggesting that c-Myc activates transcription of MDM2 (Supplemental Figure S9A). ENCODE also confirmed MDM2 is recruited to the c-Myc promoter (Supplemental Figure S9B). For further confirmation of c-Myc binding to the MDM2 promoter, in silico phylogenetic foot printing was used to identify conserved motifs in those orthologous regions [29]. We identified four c-Myc binding motifs (motifs 1–4) on the MDM2 promoter region that contained canonical enhancer box (E-box) sequences (CACGTG) (Supplemental Figure S10A), which can be bound by Myc, Myc/Max and other transcription factors [30,31].

**Figure 6:**
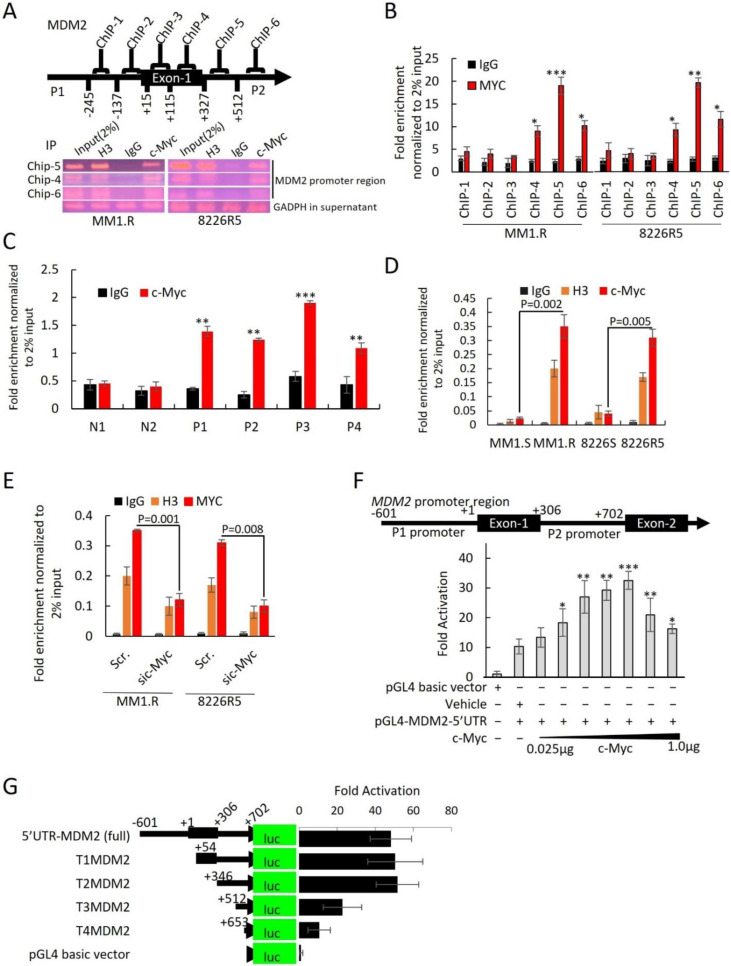
MDM2 is a direct transcriptional target of c-Myc. (**A**) Schematic diagram of the ChIP primers position on MDM2 promoter. Agarose gel image illustrating the ChIP-5, ChIP-4, and ChIP-6P regions of the promoter for 8226R5 and MM1.R cell lines that binds c-Myc after immunoprecipitation with specific anti–c-Myc Ab, negative control IgG, and positive control H3. Input represents 2% of total cross-linked, reversed chromatin before immunoprecipitation. GAPDH in the supernatant was used as loading control. (**B**) ChIP by c-Myc or IgG antibodies. The ChIP-qPCR was performed using six primer pairs for MDM2. The values were normalized to DNA level of 2% input in each sample. Mean ± SEM values of precipitation triplicates are shown. (**C**) Primary MM cells were cultured and subjected to ChIP using c-Myc antibody or normal rabbit IgG antibody (control). The precipitated chromatin was analyzed by qPCR for abundance of ChIP-5 region upstream of the MDM2 gene. Values were normalized to chromatin levels in 2% input samples. Results are presented as mean ± SEM of three independent experiments. (**D**) Parental drug-sensitive and drug-resistant MM cells were cultured and subjected to ChIP using c-Myc antibody, normal rabbit IgG (control), or H3 (control). The precipitated chromatin was analyzed by qPCR for abundance of ChIP-5 region upstream of the MDM2 gene. Values were normalized to chromatin levels in 2% input samples (MM1.S vs. MM1.R, *p* = 0.0002, 8226S vs. 8226R5 *p* = 0.0004). Results are presented as mean ± SEM of three independent experiments. (**E**) 8226R5 and MM1.R cells were transfected with 50 nM si-c-Myc for 48 h. After crosslinking the DNA/Protein complex, c-Myc, H3, or IgG antibodies were added to cell lysates. The enriched chromatin was quantified by qPCR for abundance of ChIP-5 regions of the MDM2 promoter. Values were normalized to chromatin levels in 2% input in each sample. Results are presented as mean ± SEM of three independent experiments. (**F**) The MDM2 promoter (~1300 bp in length-including P1 and P2 promoter regions) was cloned into the pGL4 vector, upstream of the luciferase reporter gene (MDM2 5′UTR (full)). Transfections were carried out using increasing amounts of c-Myc (0.025, 0.05, 0.1, 0.2, 0.25, 0.5, and 1 µg). Luciferase activity fold activation over the empty vector control is presented as mean ± SEM of three independent experiments. (**G**) Schematic representation of the luciferase reporter gene driven by various lengths of the MDM2 promoter. Five luciferase reporter constructs containing 1.3 kb (5′UTRMDM2(full)), 0.707 kb (T1MDM2 5′UTR), 0.415 kb (T2MDM2 5′UTR), 0.239 kb (T3 MDM2 5′UTR), and 0.172 kb (T4 MDM2 5′UTR) of the MDM2 promoter were PCR amplified. The products were cloned into pGL4 vectors and transfected into 293T cell lines (T3MDM2 vs. 5′UTR-MDM2 *p* = 0.04; T4MDM2 vs. 5′UTR-MDM2 *p* = 0.03). Values shown are fold activation over the empty vector control (mean + SD for three replicate experiments: *n* = 9). *, *p* < 0.05; **, *p* < 0.01, ***, *p* < 0.001.

To identify potential c-Myc regulatory sites, we performed ChIP assay. We focused on a ~1300 bp region on the *MDM2* promoter that covered all four predicted c-Myc binding motifs. Six primer ChIP pairs (ChIP-1 to ChIP-6) were designed to cover this region (Supplementary Table S1). After immunoprecipitation of c-Myc-bound DNA in MM1.R and 8226R5, we found that ChIP5, which contained c-Myc binding motif 3, was enriched with c-Myc (Figure 6A). ChIP-qRT-PCR of c-Myc bound DNA found that only ChIP-5 was significantly more enriched with c-Myc compared to control (Figure 6B). To confirm that c-Myc binds to the MDM2 promoter in MM cells, we re-performed ChIP assay on four primary MM patient samples and two normal donor samples and found that c-Myc was enriched on the MDM2 promoter of MM patient samples but not normal donor samples, suggesting that c-Myc is involved in the upregulation of MDM2 in MM pathogenesis (Figure 6C). Furthermore, ChIP-qRT-PCR on paired drug-sensitive and drug-resistant MM cells revealed that the binding of c-Myc on the MDM2 promoter was significantly increased in drug-resistant MM cells, suggesting that c-Myc is involved in the upregulation of MDM2 in drug resistance (Figure 6D). Moreover, silencing of c-Myc in drug-resistant MM cells showed less c-Myc enrichment on the MDM2 promoter in comparison to control (Figure 6E). These results indicate that c-Myc binds to the MDM2 promoter in MM cells to regulate the expression ofMDM2. To confirm that MDM2 is a direct transcriptional target of c-Myc, we cloned the MDM2 promoter region (covering promoter regions 1 and 2) into the null promoter pGL4-basic vector and performed gene transfection and reporter assays. Co-transfection with various concentrations of c-Myc expression plasmid stimulated the MDM2 promoter-mediated luciferase activity in 293T cells, suggesting that c-Myc regulates MDM2 transcription (Figure 6F).

To identify the c-Myc binding motifs in the MDM2 promoter responsible for c-Myc regulation of MDM2 expression, we carried out sequential truncation of the ~1300 bp upstream region and generated luciferase vectors. Vectors with deletions of c-Myc binding motifs 1 (T1MDM2) and 2 (T2MDM2) but with intact binding motifs 3 and 4 produced the same luciferase intensity as the vector containing the entire ~1300 bp region (5′UTR-MDM2) (Figure 6G). Conversely, we found that sequential removal of c-Myc binding motifs 3 (T3MDM2) and 4 (T4MDM2) significantly reduced luciferase intensity compared to full construct (Figure 6G). Further reporter assays demonstrated that ectopic overexpression of c-Myc resulted in an increase in MDM2 promoter activity with intact motifs as evidenced by increased luciferase activity from T1MDM2 (containing motifs 2, 3, and 4) and T2MDM2 (containing motifs 3 and 4) (Supplemental Figure S10B). Conversely, overexpression of c-Myc could not increase the MDM2 promoter activation from T3MDM2 (containing motif 4) or T4MDM2 (containing no motifs) (Supplemental Figure S10C). To further implicate c-Myc as a transcription factor forMDM2, we performed reporter assays using a T2MDM2 construct with mutated c-Myc binding motif 3. The mutated T2MDM2 construct had significantly reduced MDM2 promoter activity (~78% reduction) compared to full construct (Supplemental Figure S10D). Of note, we did not observe an increase in the activation of the mutated MDM2 promoter in the context of c-Myc ectopic overexpression (Supplemental Figure S10D). Altogether, these results indicate that c-Myc binds to the MDM2 promoter, specifically at E-box binding motifs, in MM cells to regulate the expression ofMDM2.

### 3.6. MX69 Inhibits Tumorigenesis in a MM Xenograft Model

**Figure 7:**
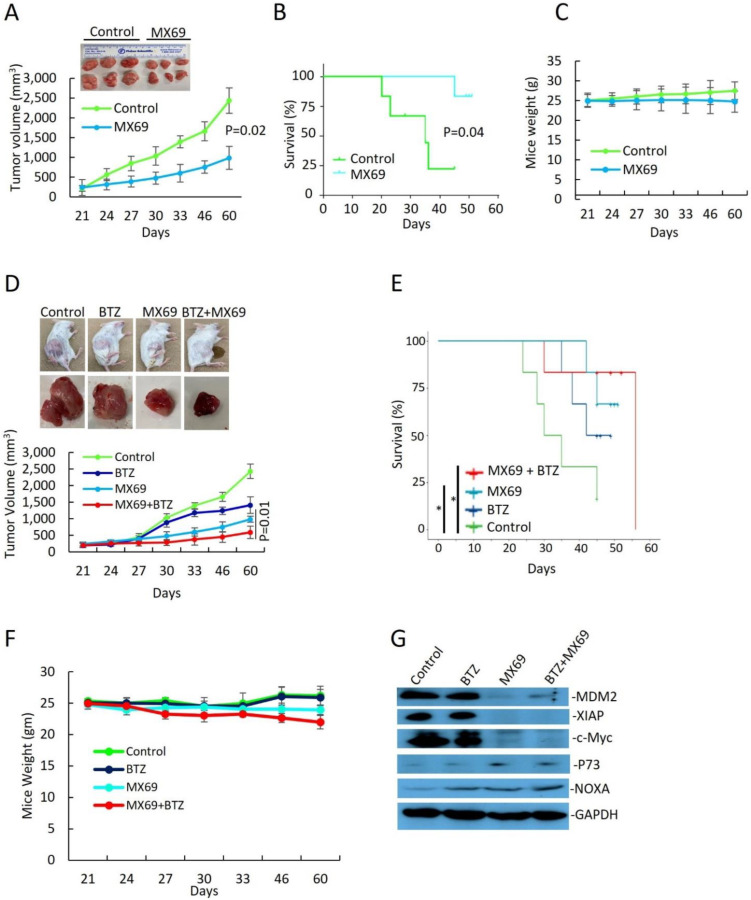
MX69 inhibits MM growth and prolongs survival in xenograft MM model. SCID mice (*n* = 6 per group) were inoculated s.c. with 1 × 10^7^ 8226R5 cells in RPMI medium along with Matrigel matrix. Tumor-bearing mice were randomly assigned into two cohorts receiving daily i.p. injection of MX69 twice a week with 0.5 mg/kg BTZ alone, three times a week with 50 mg/kg MX69 alone or combined with 0.5 mg/kg BTZ, or an equal volume of vehicle for 21 days. (**A**) Tumor volume in xenograft mice receiving drug vehicle or MX69 at different time points, as indicated. (Top) Representative images of engrafted MM tumors at mice death. (**B**) Kaplan–Meier curve indicating overall survival of xenograft mice receiving drug vehicle or MX69, as indicated. Survival analysis was performed using the Kaplan–Meier product limit method. Overall survival was calculated from the first day of tumor cell injection until death or occurrence of an event. (**C**) Body weight of xenograft mice receiving drug vehicle or MX69 at different time points, as indicated. (**D**) Tumor volume in xenograft mice receiving drug vehicle or single/combination treatment with MX69 and BTZ at different time points, as indicated. (Top) Representative images of engrafted MM tumors at mice death. (**E**) Body weight of xenograft mice receiving drug vehicle or MX69 at different time points, as indicated. (**F**) Kaplan–Meier curve indicating overall survival of xenograft mice receiving drug vehicle or single/combination treatment with MX69 and BTZ, as indicated. Survival analysis was performed using the Kaplan–Meier product limit method. Overall survival was calculated from the first day of tumor cell injection until death or occurrence of an event. (**G**) Western blot of cell lysates from engrafted MM tumors of xenografted mice receiving drug vehicle or single/combination treatment with MX69 and BTZ, as indicated. The uncropped blot is in Supplemental Figure S17. *, *p* < 0.05.

To evaluate the in vivo effects of MDM2 inhibition by MX69 in MM, we generated a MM model using SCID mice xenografted with 8266R5 cells. Treatment three times a week with 50 mg/kg MX69 alone significantly reduced tumor growth compared to vehicle control (Figure 7A, *p* = 0.02). MX69 treatment also improved survival of mice, evidenced by first death at day 24 in the control group versus day up to 60 in the treated group (Figure 7B; *p* = 0.041).

Finally, to examine if MX69 could further exert anti-myeloma effects with BTZ, we treated xenograft mice through intraperitoneal injection twice a week with 0.5 mg/kg BTZ alone, three times a week with 50 mg/kg MX69 alone or combined with 0.5 mg/kg BTZ, or an equal volume of vehicle for 21 days. Combination treatment with MX69 and BTZ was most effective at inhibiting tumor growth compared and resulted in prolonged survival compared to control or single agent treated animals (Figure 7D; on day 22 *p* = 0.012). (Figure 7E; MX69 + BTZ vs. vehicle control *p* = 0.027). When we examined isolated tumor tissues, immunoblotting demonstrated that MX69-treated groups had reduced expression ofMDM2, XIAP, and c-Myc and increased expression of pro-apoptotic factor NOXA (Figure 7G). In addition, treatment with MX69 and/or BTZ did not affect body weight, indicating the doses used for the treatment were tolerable to the mice (Figure 7C,F).

IHC staining analysis of tumor sections showed that combination treatment with MX69 and BTZ resulted in a decrease in the Ki67 proliferation index and an increase in the TUNEL apoptotic index compared to single treatment (Supplemental Figure S11A–C). Collectively, these findings indicate that in vivo targeting of MDM2 by MX69 sensitizes chemo-resistant MM cells to BTZ treatment, induces apoptosis in MM cells and suppresses MM tumor growth.

## 4. Discussion

Despite therapeutic advances in recent years, MM remains incurable and is characterized by multiple relapses, refractory disease and reduced benefit from subsequent treatments. These clinical features are especially most pronounced for patients with *TP53* inactivation [16,32]. Therefore, there is a need to identify novel targets for MM, especially in the context of TP53 inactivation. The current study presents a comprehensive investigation of MDM2 confers drug resistance in MM. Previous investigations have demonstrated correlations between MDM2 overexpression, aggressive tumor behavior, and poor prognosis in numerous cancers [5,9,33,34]. Here, we report that MDM2 overexpression is associated with progressive staging, poor prognosis, and relapse MM. To the best of our knowledge, this is the first study to demonstrate that MDM2 overexpression is associated with unfavorable MM patient outcomes.

Although MDM2 is most known for negatively regulating p53, a growing body of research suggests that MDM2 regulates many other oncogenic pathways. As such, targeting these pathways may hold therapeutic potential even in the context of TP53 inactivation. Here, we further implicate MDM2 as a multi-functional oncogenic factor with novel evidence that MDM2 stabilizes c-Myc mRNA to promote c-Myc translation [18]. c-Myc is a central oncogene in MM development responsible for the progression from precursor stages to overt MM [35]. c-Myc is constitutively and aberrantly expressed in over 50% of cancers including MM [26,36,37]. In the present study, we found a positive correlation between MDM2 and c-Myc expression in MM cells (Figure 5A,C). In contrast to the prior study from Tran et al. (2020) which reports that MDM2 does not regulate c-Myc expression in neuroendocrine cancers [38], we found that manipulation of MDM2 expression level by siRNA or ectopic overexpression resulted in reciprocal changes in c-Myc expression level in MM cells. Our bioinformatic analysis revealed that c-Myc mRNA contains 3′UTR AU-rich elements (AREs) which have been shown to mediate rapid mRNA turnover, attenuation of translation and mRNA instability [39]. In neuroblastoma, it has been reported that the 3′UTRs of VEGF and MYCN, a homologue of c-Myc, contain AREs that can be bound to, and stabilized by MDM2 [19,20,21,40,41]. Similarly, we found that c-Myc 3′UTR contains AREs that can be bound and stabilized by MDM2. Our RNA-protein binding assay (Figure 5D), RNA-Protein UV-crosslinking assay (Figure 5F) and luciferase assay (Figure 5H) revealed that MDM2 directly binds and stabilizes c-Myc 3′UTR to enhance c-Myc translation in MM cells.

c-Myc is a major transcription factor implicated in the transcriptional activation of many oncogenes. The c-Myc protein contains a C-terminal basic/helix-loop-helix/leucine-zipper (bHLHzip) domain and an N-terminal transactivation domain. The bHLHzip domain allows dimerization of Myc with the protein MAX, which is a prerequisite for specific binding to DNA at E-box sequences (CACGTG) in target gene promoters [42]. It has been shown that dimerization with MAX and specific binding to DNA are required for induction of cell cycle progression, apoptosis, and transformation by c-Myc [42,43]. Our bioinformatics analysis revealed that the MDM2 promoter contains E-box sequences, which are potential binding sites for c-Myc to bind and activate transcription. Our luciferase assays revealed that c-Myc drives transcription from the MDM2 promoter, which was abolished when key c-Myc binding motifs were deleted or mutated (Figure 6G). These findings are consistent with the prior report which revealed that manipulation of c-Myc expression level resulted in reciprocal changes in MDM2 expression in retinoblastoma cells [38].

**Figure 8:**
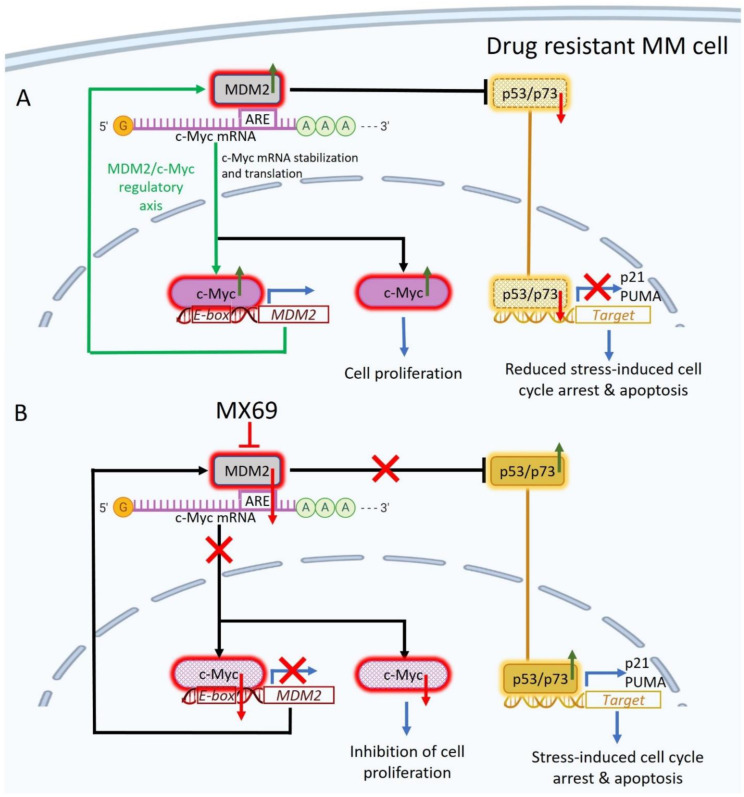
Targeting the MDM2/c-Myc regulatory axis in MM. (**A**) Proposed model of MDM2/c-Myc regulatory axis in MM. MDM2 binds c-Myc 3′UTR AU-rich elements (AREs) to promote c-Myc mRNA stabilization, and c-Myc drives transcription of MDM2 via binding E-box sequences in MDM2 promoter. As a result, MDM2 is upregulated and targets p53/p73 for proteasomal degradation, thereby preventing the induction of p21 and PUMA for stress-induced cell cycle arrest and apoptosis. In addition, c-Myc is upregulated and induces expression of cell proliferation genes. (**B**). MDM2 inhibitor MX69 disrupts the MDM2/c-Myc axis in MM. MX69-mediated downregulation of MDM2 results in the destabilization of c-Myc mRNA, leading to reduced expression of c-Myc protein and its transcriptional targets involved in cell proliferation. MX69-mediated downregulation of MDM2 also results in the accumulation of p53 and p73, leading to increased expression of its transcriptional targets such as p21 and PUMA for stress-induced cell cycle arrest and apoptosis.

In addition to uncovering a novel MDM2/c-Myc regulatory loop involved in oncogenesis, our study provides the rationale for the use of a newly discovered small molecule inhibitor MX69 for the treatment of MM. MDM2 inhibitors have demonstrated robust anti-cancer activity in clinical trials for glioblastoma, sarcoma and various hematological malignancies such as B-cell chronic lymphocytic leukemia [44]. However, the majority of these agents target MDM2/p53 interaction and have limited anti-cancer effects in the context of TP53 inactivation. In prior studies, MX69 was shown to be effective in neuroblastoma and leukemia cells leading to inhibition of cell proliferation and cell cycle progression as well as induction of apoptosis irrespective of p53 status [22]. Consistently, we found that MX69 treatment in MM cells efficiently inhibited MM cell proliferation, cell cycle progression, colony formation and migration, as well as induced apoptosis irrespective of p53 status by targeting the MDM2-c-Myc regulatory loop (Figure 8). Furthermore, our study suggests that inhibition of MDM2 could re-sensitize drug-resistant MM cells to current anti-myeloma drugs, providing the rationale for the use of MX69 in combination therapy. We also found that MX69 inhibited the MDM2/c-Myc regulatory axis in vivo resulting in significant inhibition of tumor growth and prolonged survival of MM xenografted mice. To the best of our knowledge, this is the first study to demonstrate in vivo toxicity of MDM2 inhibitors in drug-resistant MM xenograft models. We acknowledge that MX69 may have off-target effects, independent of MDM2, leading to further MM growth inhibition; future studies are warranted to fully elucidate such additional targets and possible other mechanisms underlying MX69′s anti-cancer properties.

## 5. Conclusions

In conclusion, we have uncovered a novel mechanism underlying cancer development. For the first time, we demonstrate that MDM2 and c-Myc reciprocally regulate each other via a positive feedback loop and MX69 is an effective anti-myeloma agent that re-sensitizes drug-resistant MM cells to conventional MM therapies.

## Data Availability

Not applicable.

## Supplementary Materials

The following supporting information can be downloaded at: https://www.mdpi.com/article/10.3390/cancers14061592/s1, Supplemental Table S1: ChIP Assay Primers for MDM2 promoter, Supplemental Table S2: c-Myc Primers, Supplemental Table S3: MDM2 primers; Supplemental Figure S1: MDM2 overexpression is associated with poor outcomes in MM patients, Supplemental Figure S2: Knockdown of MDM2 induces growth inhibition, apoptosis and cell cycle arrest in drug resistant MM cells, Supplemental Figure S3: MX69 inhibits MM cell growth, apoptosis and cell cycle arrest in drug-resistant MM cells, Supplemental Figure S4: MX69 induces apoptosis and inhibits colony formation and migration in MM, Supplemental Figure S5: Combination of MX69 with other anti-myeloma drugs show synergistic effects on resistant MM cells, Supplemental Figure S6: c-Myc and MDM2 expressions are positively correlated in resistant MM cells, Supplemental Figure S7: MDM2 overexpression reciprocally induces overexpression of c-Myc, Supplemental Figure S8: Effect of MDM2 KD on c-Myc expression, Supplemental Figure S9: c-Myc transcription factor binding site on the MDM2 promoter regions, Supplemental Figure S10: MDM2 is a direct transcriptional target of c-Myc, Supplemental Figure S11: Immunohistochemistry of tumor sections from mice treated with control, BTZ, MX69 and BTZ+MX69 and respective scoring, Supplemental Figure S12: Uncropped blots for Figure 1F, Supplemental Figure S13: Uncropped blots for Figure 2D, Supplemental Figure S14: Uncropped blots for Figure 3D, Supplemental Figure S15: Uncropped blots for Figure 4A, Supplemental Figure S16: Uncropped blots for Figure 5A, Supplemental Figure S17: Uncropped blots for Figure 7G, Supplemental Figure S18: Uncropped blots for Figure S4A, Supplemental Figure S19: Uncropped blots for Figure S4B, Supplemental Figure S20: Uncropped blots for Figure S5E, Supplemental Figure S21: Uncropped blots for Figure S7A, Supplemental Figure S22: Uncropped blots for Figure S7B, Supplemental Figure S23: Uncropped blots for Figure S7C, Supplemental Figure S24: Uncropped blots for Figure S7D, Supplemental Figure S25: Uncropped blots for Figure S8D.
