# Supplementary material for: Targeting an MDM2/MYC Axis to Overcome Drug Resistance in Multiple Myeloma
Source: Cancers (Basel). 2022 Mar 21;14(6):1592. doi: 10.3390/cancers14061592 (PMC8945937; doi:10.3390/cancers14061592)
Supplement: Supplementary file 1 [file cancers-14-01592-s001.zip › cancers-1610285-supplementary.pdf]

cancers

Targeting an MDM2/MYC Axis to Overcome Drug Resistance in Multiple Myeloma

Omar Faruq, Davidson Zhao, Mariusz Shrestha, Andrea Vecchione , Eldad Zacksenhaus and Hong Chang

Materials and Methods

1. Drug treatment

MX69 was purchased from Selleckchem, dissolved in DMSO to make a 50 mmol/L stock solution, and stored at −20°C until use. In some experiments, cells were simultaneously exposed to MX69 and Bortezomib (Selleck Chemicals, USA), Lenalidomide (Selleck Chemicals, USA); Dexamethasone Sodium Phosphate, (Selleck Chemicals, USA); Doxorubicin (Adriamycin), (Selleck Chemicals, USA). Cell lines were harvested in log-phase growth and exposed to drug for different time periods and at different concentrations, as indicated. In each experiment, the final DMSO concentration was kept constant and did not exceed 0.1% (v/v). After drug treatment, cells were harvested and subjected to further analysis as described below.

2. Cell viability assay

Cell viability was assessed by MTT colorimetric assay. Cells were seeded in 96-well plates (Sarstedt, Inc.) in 100 µL complete medium at a density of 10-20×10^4 cells per well and were incubated with drugs at different concentrations and durations as indicated. After incubation, MTT (0.3 mg/mL) was added to each well, and cells were further incubated for an additional 4 hours. This was followed by the addition of DMSO to the wells and 30 minutes incubation at 37°C. Following incubation, the absorbance of the cells was read with a microplate reader set at a test wavelength of 570 nm and a reference wavelength of 650 nm. Cell survival is expressed as the percentage of negative control cells.

3. Cell cycle and apoptosis assay

Flow cytometry was performed to assess cell cycle distribution and apoptosis. For cell cycle analysis, cells were collected, rinsed, and fixed in 70% ethanol, and then washed again and re-suspended in PBS solution containing propidium iodide (PI). The data from flow cytometry was analyzed using a FACScan. For quantitative detection of apoptosis, cells were washed with PBS and stained with APC annexin V and PI using an Annexin V Apoptosis Detection Kit 1 (Biolegend, USA). Annexin V is detected in both early and late stages of apoptosis, whereas PI is detected only in late apoptosis or necrosis. Early apoptotic cells were positive for Annexin V and negative for PI (lower right quadrant); late apoptotic cells were positive for both Annexin V and PI (upper right quadrant). The data from flow cytometry was analyzed using Express 7 software or Flowjo. The extent of apoptosis was assessed as the percentage of Annexin V+ cells.

4. Quantitative real-time PCR

RNA expression was determined by qRT-PCR. Briefly, total RNA was extracted using TRIzol reagent (Invitrogen, USA). cDNA synthesis was performed according to manufacturer's protocol (Bio-Rad, USA). Ct values were measured and normalization to GAPDH and other housekeeping genes. Fold changes in gene expression were determined using the $2^{-\Delta\Delta Ct}$ algorithm.

5. Immunoblotting

Whole-cell lysates were prepared by extraction of cell pellets, which were lysed for 30 minutes on ice in 1X RIPA buffer. Protein concentrations were measured using a spectrophotometer (Thermo Fisher Scientific, USA). Equal amounts of protein extracts were resolved using 10-12% SDS-PAGE and transferred to a polyvinylidene difluoride membrane (Bio-Rad, USA). After blocking for 1 hour at room temperature with PBS containing either 5% skim milk or 2.5% bovine serum albumin, the membranes were probed with primary antibodies. Next, the membrane was washed and incubated with horseradish peroxidase secondary antibodies for 1 hour, and the blots were developed using an ECL chemiluminescent detection system (Bio-Rad, USA).

6. Co-immunoprecipitation (Co-IP)

A modified Triton buffer (1% Triton X-100, 20mM Tris-HCl with pH 7.4, 2.5mM EDTA, 2.5mM ethylene glycol tetra-acetic acid, 140mM NaCl, and inhibitors) was used to lyse cells. Protein extracts were incubated overnight at 4°C with antibodies, followed by A-/G-protein beads (Amersham) for 24 hours. The non-immunoprecipitated, unbound fraction in the supernatant was recovered by centrifugation. The immunoprecipitated, bound fraction associated with A-/G-protein beads were washed with lysis buffer. Proteins were resolved on 10% SDS-PAGE. Membranes were probed with monoclonal and polyclonal antibodies, as indicated.

7. Chromatin immunoprecipitation (ChIP)

Cells were treated in culture with 1% formaldehyde for 10 minutes at 37°C. Cells were harvested by centrifugation, washed with 2 x 10 mL PBS, and chromatin was immunoprecipitated using the SimpleChIP Enzymatic Chromatin IP Kit (Magnetic Beads) (Cell Signaling Technology, USA). Universal positive control H3 used for CHIP assay was applied when chipping active genes (Histone H3 (D2B12) XP Rabbit mAb (ChIP Formulated) #4620). After immunoprecipitation, 2 ng DNA was used for each real-time PCR run. ChIP assay primers for MDM2 promoter are listed in Supplemental Table S1.

8. Soft agar colony formation assay and migration assay

Colony formation assay and migration assay were performed as previously described[24]. For colony formation assay, cells at a density of 5×10^4 cells/mL were plated onto methyl cellulose medium for 21 days. Cell clusters that consisted of >40 cells were counted as colonies upon examination by inverted microscope. For migration assay, cells at a density of 1×10^4 were placed into 24-well Transwell inset chambers (8μM inserts, Corning Inc., USA) according to manufacturer's protocol and as previously described [24]. Migration was quantified by counting the number of cells on the lower surface of the filters. Experiments were performed in triplicate.

9. Myeloma xenograft mouse model

Immunodeficient (SCID) mice (Female, 6–8 weeks old; OCI) were housed in the animal care facility, under a 12/12 hours light/dark cycle at 22°C, and received a standard diet and acidified water ad libitum. Using a protocol approved by the animal testing ethics committee of UHN, mice were inoculated subcutaneously at their lower dorsum with 1×10^7 8226R5 cells in matrigel basement membrane matrix (Becton Dickinson, USA). When tumors were palpable (approximately 21 days after injection), mice were randomly assigned into 4 groups (n=6 in each), receiving intraperitoneal injection twice a week with 0.5 mg/kg BTZ alone, three times a week with 50 mg/kg MX69 alone or combined with 0.5 mg/kg BTZ, or an equal volume of vehicle for 21 days. The shortest and longest diameters of the tumor were measured with external calipers every 3 days, and tumor volume (mm^3) was calculated using the following standard formula: V = 0.5a x b2, where "a" and "b" are the long and short diameter of the tumor. Survival was evaluated from the first day of

tumor injection until death or occurrence of an event. In accordance with institutional guidelines, mice were sacrificed when their tumors reached 1.5 cm in diameter or in the event of paralysis or major compromise in quality of life to prevent suffering.

10. Pulse–chase assay

The turnover of protein was assayed by a standard protein synthesis inhibitor cycloheximide (CHX) assay. Briefly, cells were treated with 50 μg/ml CHX for different durations before lysis in the presence of siMDM2, scrambled siRNA control, MX69 or DMSO control. Protein expression was detected by western blot analysis.

11. UV cross-linking, Gel-shift assay, and RNA-protein binding assays

UV cross-linking and immunoprecipitation assays were performed as previously described [18]. Briefly, DNA templates for synthesis of the C-Myc 3′UTR RNA probe (probe 1) and the C-Myc 5′UTR IRES RNA probe (probe 2) were generated by PCR using specific primers: Probe 1: forward: 5′TAATACGAGTCACTATAGGGACCTCACAACCTT-GGCTGAGT3′, reverse: 5′GGATTGAAATTCTGTGTAACTGC-3′. Probe 2: forward: 5′TAATACGAGTCACTATAGGGACCTCCCATATTCTCCCGTCT3′, reverse: 5′TGTGTCTGCCTGTTCCAGAG 3′. The forward primers incorporated the T7 promoter sequence (underlined). Probe 1 with mutations in ARE was also synthesized. Internally-labeled RNA probes were synthesized by *in vitro* transcription with T7 polymerase using MAXIscript T7 Transcription Kit (Ambion, USA) in the presence of biotinylated-UTP (Thermo Fisher Scientific, USA). The extracts from 8226R5 cells extracts were mixed with biotinylated labeled RNA probes. UV cross-linking of the RNA-protein complexes was performed using a 254-nm UV light set at 400,000μJ/cm^2. UV-irradiated RNA-protein complexes were treated with RNase T1. UV-irradiated RNA-protein complexes were pulled down by streptavidin-coupled Dynabeads (Thermo Fisher Scientific, USA), resolved by 10% SDS-PAGE gel and visualized by immunoblotting.

For gel shift assay, pre-designed unlabeled RNA probes were synthesized by *in vitro* transcription with T7 polymerase using MAXIscript T7 Transcription kit (Ambion, USA). For UV cross-linking assay, endogenous MDM2 protein was isolated and purified using direct immunoprecipitation (Abcam Immunoprecipitation (IP) Protocol) from 8226R5 cells extract. UV-irradiated RNA-protein complexes were resolved by 6% Native-PAGE gel and visualized by immunoblotting.

For protein-RNA binding assay, c-Myc mRNA was co-immunoprecipitated from whole-cell extracts as previously described [18]. Briefly, 8226R5 cells were harvested by low-speed centrifugation at 4°C. The cell pellets were resuspended in 100μL of RNA binding buffer (20 mM Tris [pH 7.5], 150 mM NaCl, 5 mM $MgCl_2$, 0.1% NP-40, 50 μM $ZnCl_2$, 2% glycerol, 1mM DTT) supplemented with 10U of RNase inhibitor. Cell extracts were further prepared by freeze-thaw method. Whole-cell extracts were incubated for 60 minutes at room temperature with 5μL of monoclonal anti-MDM2 antibody, anti-HuD antibody, or anti-Actin antibody and 20μL of protein A plus G agarose beads and 5U of RNase inhibitor. Next, the beads were washed extensively with RNA binding buffer supplemented with RNase inhibitor. The RNA that was associated with the antibody-antigen complexes was isolated by performing repeated phenol-chloroform extractions and then precipitating with 2M ammonium acetate and 3 volumes of cold ethanol. The resulting RNA was analyzed by traditional RT-PCR, using c-Myc-specific primers.

12. Discovery of c-Myc DNA binding motifs by MEME analysis:

5kb upstream sequences of human MDM2 gene and its orthologs (collected from ENSEMBLE) were analyzed by Multiple Expectation maximizations for Motif Elicitation (MEME), to identify potential binding sites conserved across species. Since PWMs (position weight matrix) for various transcription factors (TFs) have already been reported in

JASPAR, UniPROBE, Jolma et al and TRANSFAC public databases, based on a comparison of the similarity between the reported PWM of a TF to the footprinted PWM in the orthologous upstream regions, it is possible to predict the TFs which are most likely to bind to these predicted binding sites. Tomtom is a tool in the MEME-suite which compares DNA motifs to known motifs included in these databases. PWMs of various discovered motifs were used as input file for Tomtom and compared with already reported PWMs of TFs in the above-described databases to identify the potential c-Myc binding to the MDM2 upstream regions. c-Myc associations identified at a significance level of $p=1x10^{-3}$ were considered statistically significant.

13. Reporter construct

13.1. For c-Myc

To generate the pGL3-c-Myc 3′-UTR reporter plasmid, we generated a 498 bp segment of the c-Myc 3′UTR, from 1 bp downstream of the stop codon to 55 bp upstream of the end of the c-Myc mRNA. The insert was amplified by RT-PCR from 8226R5 cells genomic DNA using the forward primer 5′ CTAG **TCTAGA** GTCCTGAGCAATCACCTATGAACT 3′ and reverse primer 5′ CTAG **TCTAGA** AACAAACAGGGATGGTGGTG 3′**.** The PCR product was isolated by agarose gel electrophoresis and digested with ***XbaI*** (restriction site underlined in the primers). The final digested PCR product was subcloned into a downstream portion of the luciferase reporter gene in the pGL3.0-promoter vector (Promega, USA). Promoter region sequence of generated vectors was confirmed by restriction digestion and Sanger sequencing. The sequence of the c-Myc 3′UTR segment linked to the luciferase plasmid (pGL3P) corresponding to nucleotides 1764 to 2262 of the human c-Myc mRNA (GenBank accession no. NM_002467.6). The mutant 3′UTR clone was constructed by introducing several mutations (Supplemental Table S2) in ARE sites in the 3′UTR of c-Myc by using Quik Change II Site-Directed Mutagenesis Kit (Agilent Technologies, USA**)**.

13.2. For MDM2 5′UTR

According to Ensembl database ENSG00000135679 MIM:164785, the 1269bp MDM2 upstream promoter region (including promoter-1, exon-1 and promoter) was amplified by PCR from 8226R5 cell genomic DNA using the forward primer 5′ GCTCGG ***GGTACC*** ACCAGAACAGCTGTATGCAC 3′ and reverse primer 5′ CCGAGC ***CTCGAG*** ACGTGTCTGA ACTTGACCAG 3′**.** The PCR product was isolated by agarose gel electrophoresis and digested with ***Kpn*I and *XhoI*** (restriction sites underlined in the primers) to generate the sticky ends. The final digested PCR product was subcloned into an upstream portion of the luciferase reporter gene in the pGL4.2-basic vector (Promega, USA). Promoter region sequence of generated vectors was confirmed by restriction digestion and Sanger sequencing. The new constructed plasmid was named 5′UTR MDM2(full)**.** A series of reporter truncation/deletion plasmids containing various portions of the 1269 bp MDM2 promoter region were generated using 5′UTR MDM2(full) as a template. The forward PCR primer sequences used for each promoter reporter construct and name are listed in Supplemental Table S3. The reverse primer was the same as that used for the full length promoter region. The length shown in the row of each plasmid in Supplemental Table S3 represents the approximate distance from the translation start site in the MDM2. All constructs were verified by restriction enzyme digestion and Sanger sequencing. The mutant T2 MDM2 5′UTR clone was constructed by introducing several mutations in E-box/Motif-3 site in the 5′UTR of T2 MDM2 by using Quik Change II Site-Directed Mutagenesis Kit (Agilent Technologies, USA).

14. Gene transfection and reporter assay

To overexpress MDM2 or c-Myc in cells, the MDM2-WT, c-Myc or control plasmids were transiently transfected into MM1.S and 8226S cells, respectively, by Lipofectamine

3000 Transfection Reagent (Invitrogen, USA). Cells were collected and lysed after 48 hours post-transfection for Western blot and RT-PCR. For transient MDM2 or c-Myc siRNA or scramble siRNA transfection in MM1.R, 8226R5, MM1.S, 8226S we used Lipofectamine 3000 Transfection Reagent (Invitrogen, USA).

Transient transfection was performed to examine the effects of MDM2 on the c-Myc promoter, IRES and 3′UTR activities: 293T and 8226S cells were co-transfected with MDM2-WT expression plasmids and either pGL3-c-Myc-Pro, pGL3-c-Myc 5′UTR or pGL3-c-Myc 3′UTR reporter plasmids with Lipofectamine 3000 Transfection Reagent (Invitrogen, USA). The pIS2 vector (which was derived from PRL_SV40 vector) was co-transfected in the transfection of pGL3-c-Myc-Pro, pGL3-c-Myc 5′UTR and pGL3-c-Myc 3′UTR plasmids, to provide an internal control. For the 8226 cells, double transfection was performed. Cell extracts were prepared and collected after 48 hr transfection using 1× lysis buffer, then 20 µL aliquots of the supernatant were mixed first with 100 µL of Luciferase Assay Reagent II (Promega, USA) to measure the FL activity The RL activity was determined by adding Stop & Glo® Reagent to the same sample. Luciferase activities were analyzed via Microplate Instrumentation (BioTek, USA).

Transient transfection was performed to examine the effects of c-Myc on the MDM2 promoter. 293T cells were co-transfected with c-Myc expression plasmids and either 5′UTR MDM2(full), T1MDM2 5′UTR, T2 MDM2 5′UTR, T3 MDM2 5′UTR and T4 MDM2 5′UTR plasmids with Lipofectamine 3000 Transfection Reagent (Invitrogen, USA). The pIS2 vector (which was derived from PRL_SV40 vector) was co-transfected in the transfection of 5′UTR MDM2(full), T1MDM2 5′UTR, T2 MDM2 5′UTR, T3 MDM2 5′UTR and T4 MDM2 5′UTR plasmids, to provide an internal control. Cell extracts were prepared and collected after 48 hr transfection using 1 × lysis buffer, then 20 µL aliquots of the supernatant were mixed first with 100 µL of Luciferase Assay Reagent II (Promega, USA) to measure the FL activity. The RL activity was determined by adding Stop & Glo® Reagent to the same sample. Luciferase activities were analyzed via Microplate Instrumentation (BioTek, USA).

15. Statistical analysis

Synergistic effect (combination index(CI)<1.0) of drug combinations was analyzed using CompuSyn software (Chou and Martin). Cell viability assay was performed for each drug alone at varying concentrations to generate dose-effect curves for each drug. Cell viability assay was performed for combination treatment using non-constant drug ratios, holding one drug concentration constant and changing the other drug concentrations. CI was calculated using the Chou-Talalay equation: CI = (D)1/(Dx)1 + (D)2/(Dx)2, where (D)1 and (D)2 are concentrations of drug 1 or 2 that give x fraction affected (Fa) when used in combination and (Dx)1 and (Dx)2 are concentrations of drug 1 or 2 that give the same x Fa when used alone.

All quantified data represent a mean of triplicate experiments ± SD. Student's t-test was used to evaluate differences in means between groups. Correlation between MDM2 and c-Myc expression was determined using Pearson's coefficient test. All p-values were 2-sided, and all statistical tests were performed in GraphPad Prism 8 (GraphPad Software, La Jolla, CA). *, P < 0.05; **, P < 0.01, ***, P<0.001.

16. Drugs and reagents

- Bortezomib, Selleck Chemicals, Houston, TX, USA
- Lenalidomide (CC-5013), Selleck Chemicals, Houston, TX, USA
- Dexamethasone Sodium Phosphate, Selleck Chemicals, Houston, TX, USA
- Doxorubicin (Adriamycin), Selleck Chemicals, Houston, TX, USA
- MX69, Selleck Chemicals, Houston, TX, USA
- Cycloheximide (CHX), BioShop Canada Inc., Burlington, ON, CAN
- MTT, BioShop Canada Inc., Burlington, ON, CAN

All chemical agents were dissolved in dimethyl sulfoxide except special indication and further diluted in RPMI1640 medium.

17. Antibodies

- Anti-MDM2, Santa Cruz Biotechnology, Dallas, TX, USA and SAB Signalway Antibody, Inc. Danvers, MA USA
- Anti-MDM2, SAB Signalway Antibody, Inc. Danvers, MA, USA
- Anti-c-Myc, Santa Cruz Biotechnology, Dallas, TX, USA
- Anti-c-Myc, Cell Signaling Technology, Inc. Danvers, MA, USA
- Anti-XIAP, Santa Cruz Biotechnology, Dallas, TX, USA
- Anti-p53, Santa Cruz Biotechnology, Dallas, TX, USA
- Anti-p53, EMD Millipore, Billerica, MA, USA
- Anti-p73, Santa Cruz Biotechnology, Dallas, TX, USA
- Anti-p21, Waf1/Cip1 (12D1), Cell Signaling Technology, Inc. Danvers, MA, USA
- Anti-PUMA, SAB Signalway Antibody, Inc. Danvers, MA, USA
- Anti-PARP, Santa Cruz Biotechnology, Dallas, TX, USA
- Anti-Caspase-3, Cell Signaling Technology, Inc. Danvers, MA, USA
- Anti-Cleaved Caspase-3, Cell Signaling Technology, Inc. Danvers, MA, USA
- Anti-NOXA, Santa Cruz Biotechnology, Dallas, TX, USA
- Anti-H3, Cell Signaling Technology, Inc. Danvers, MA, USA
- Anti-Caspase-3, Cell Signaling Technology, Inc. Danvers, MA, USA
- Anti-Cleaved Caspase-3 (Asp330), Cell Signaling Technology, Inc. Danvers, MA, USA
- Anti-p21, Cell Signaling Technology, Inc. Danvers, MA, USA
- Anti-HuD, Invitrogen, Thermo Fisher Scientific, Waltham, MA, USA
- Anti-GAPDH, Cell Signaling Technology, Inc. Danvers, MA, USA
- Anti-β-Actin, Sigma-Aldrich, St. Louis, MO, USA

18. List of plasmids

1. pcDNA3 MDM2 WT was a gift from Mien-Chie Hung (Addgene plasmid # 16233 ; http://n2t.net/addgene:16233 ; RRID:Addgene_16233)
2. pcDNA3.2/EV was a gift from Jan Rehwinkel (Addgene plasmid # 120833 ; http://n2t.net/addgene:120833 ; RRID:Addgene_120833)
3. pCDH-Flag-c-Myc was a gift from Hening Lin (Addgene plasmid # 102626 ; http://n2t.net/addgene:102626 ; RRID:Addgene_102626)
4. pFRT-TODestGFP_HuD was a gift from Thomas Tuschl (Addgene plasmid # 65763 ; http://n2t.net/addgene:65763 ; RRID:Addgene_65763)
5. pIS2 was a gift from David Bartel (Addgene plasmid # 12177 ; http://n2t.net/addgene:12177 ; RRID:Addgene_12177)

Table S1. ChIP Assay Primers for MDM2 promoter

Primer Name	Forward primer	Reverse primer	Position	Amplicon Length(bp)
ChiP-1	5'CGCCAAACCTTGCTGGCT 3'	5'ACCTCCGGGATGATGGAGTG3'	-245 to -119	126
ChiP-2	5'CACTCCATCATCCCGGAGGT3'	5'CCAGAAGCAGCCAAGCTC3'	-137 to+32	169
ChiP-3	5'GAGCTTGGCTGCTTCTGG3'	5'TGGCTGCGAAAGCAGCAG3'	+15 to +132	117
ChiP-4	5'CTGCTGCTTTCGCAGCCA3'	5'AGCCCAGACCCAAAAGTGAC3'	+115 to +347	323
ChiP-5	5'GTCACTTTTGGGTCTGGGCT3'	5'CGAGAGCCGTCCGAAATC3'	+327 to +531	224

ChiP-6	5’GATTTCGGACGGCTCTCG3’	5’TACGCGCAGCGTTCACAC3’	+513 to +678	165

Table S2. c-Myc Primers.

Name of Primers	Primer Sequences
c-Myc P.F	5’GCAGGTGAGAAGGTGAGAGG3’
c-Myc R.P	5’ACAGAATGGGTCCAGATTGC3’
FP_Mut1_Myc3utr	5’CTTGAGACTGAAAGCGCGCGCCATAATGTAAAC3’
RP_Mut1_Myc3utr	5’GTTTACATTATGGCGCGCGCTTTCAGTCTCAAG3’
RP_Mut2_Myc3utr	5’ACAGATTTGTCGCGCAGAATTGTTT3’
RP_Mut2_Myc3utr	5’AAACAATTCTGCGCGACAAATCTGT3’
FP_Mut3_Myc3utr	5’AAAATTTTAAGCGCGGCACAATGTTTCTC3’
RP_Mut3_Myc3utr	5’GAGAAACATTGTGCCGCGCTTAAAATTTT3’
FP_Mut4_Myc3utr	5’CTAATTTTTTTTGCGGCAGTACATTTTGC3’
RP_Mut4_Myc3utr	5’GCAAAATGTACTGCCGCAAAAAAAATTAG3’

Table S3. MDM2 primers.

Name of primer	Primers sequences
MDM2 F.P	5′-TGGGCAGCTTGAAGCAGTTG-3’
MDM2 R.P	5′-CAGGCTGCCATGTGACCTAAGA-3’
T-1.MDM2 F.P	5’GCTCGG *GGTACC* CGGAAAGATGGAGCAAGAAG3’
T-2.MDM2 F.P	5’GCTCGG *GGTACC* TCTGACGGTGTCCCCTCTATC3’
T-3.MDM2 F.P	5’GCTCGG *GGTACC* GGATTTCGGACGGCTCTC3’
T-4.MDM2 F.P	5’GCTCGG *GGTACC* TCGGGTCACTAGTGTGAACG3’
T2.MDM2_Mut F.P	5’GCGCGGGGCATCGGGGtactacaGCTTTGCGGAGGTTTTG3’
T2.MDM2_Mut R.P	5’CAAAACCTCCGCAAAGCtgtagtaCCCCGATGCCCCGCGC3’

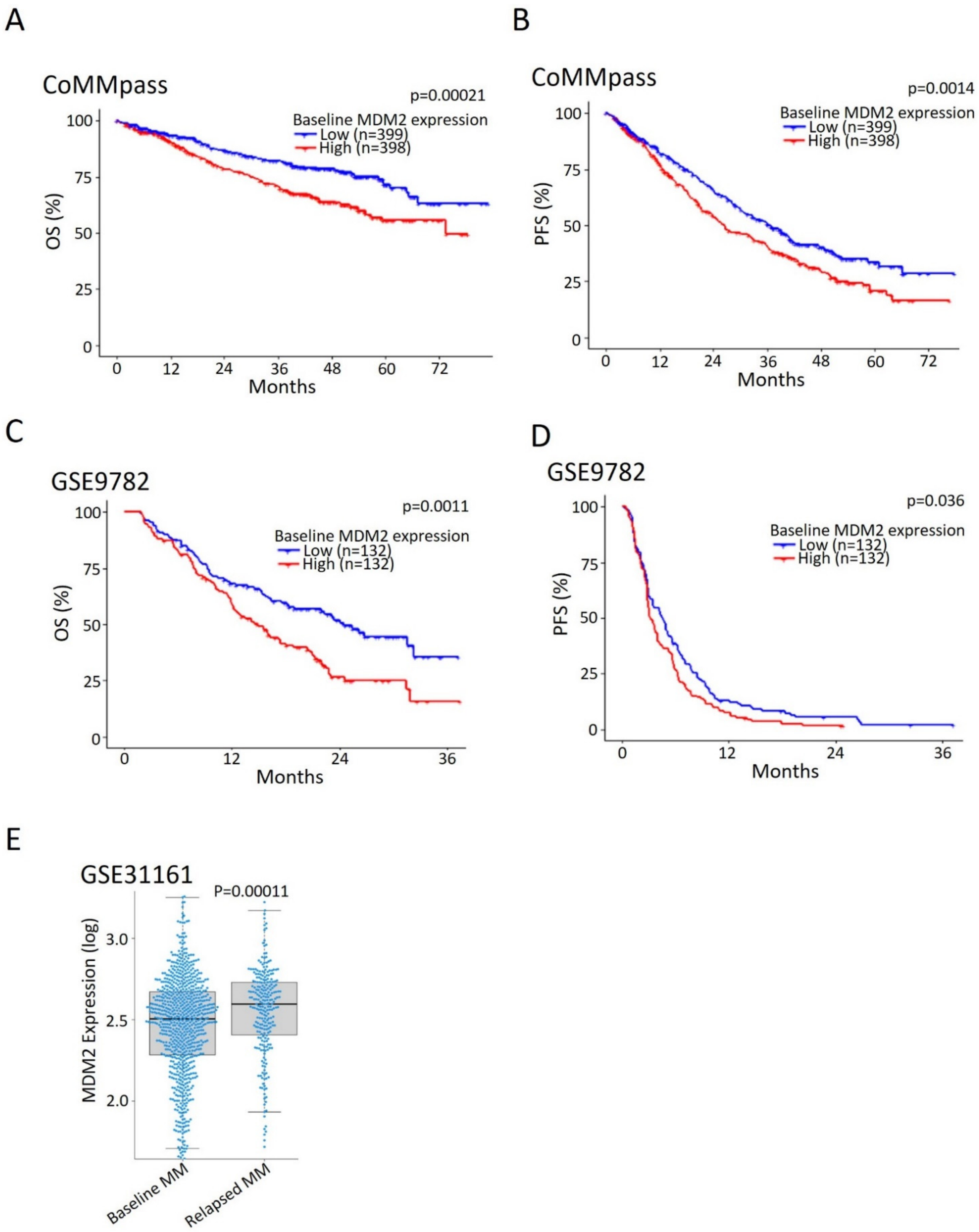

Figure S1. MDM2 overexpression is associated with poor outcomes in MM patients. A–D. Kaplan-Meier plots indicate the overall survival (OS) (**A**,**B**) and progression free survival (PFS) (**C**,**D**) for MM patients stratified by MDM2 expression (CoMMpass N = 797; GSE9782 N = 264). (**E**) MDM2 expression before and after relapse in MM (GSE31161; baseline MM n = 780 and relapsed MM n = 255).

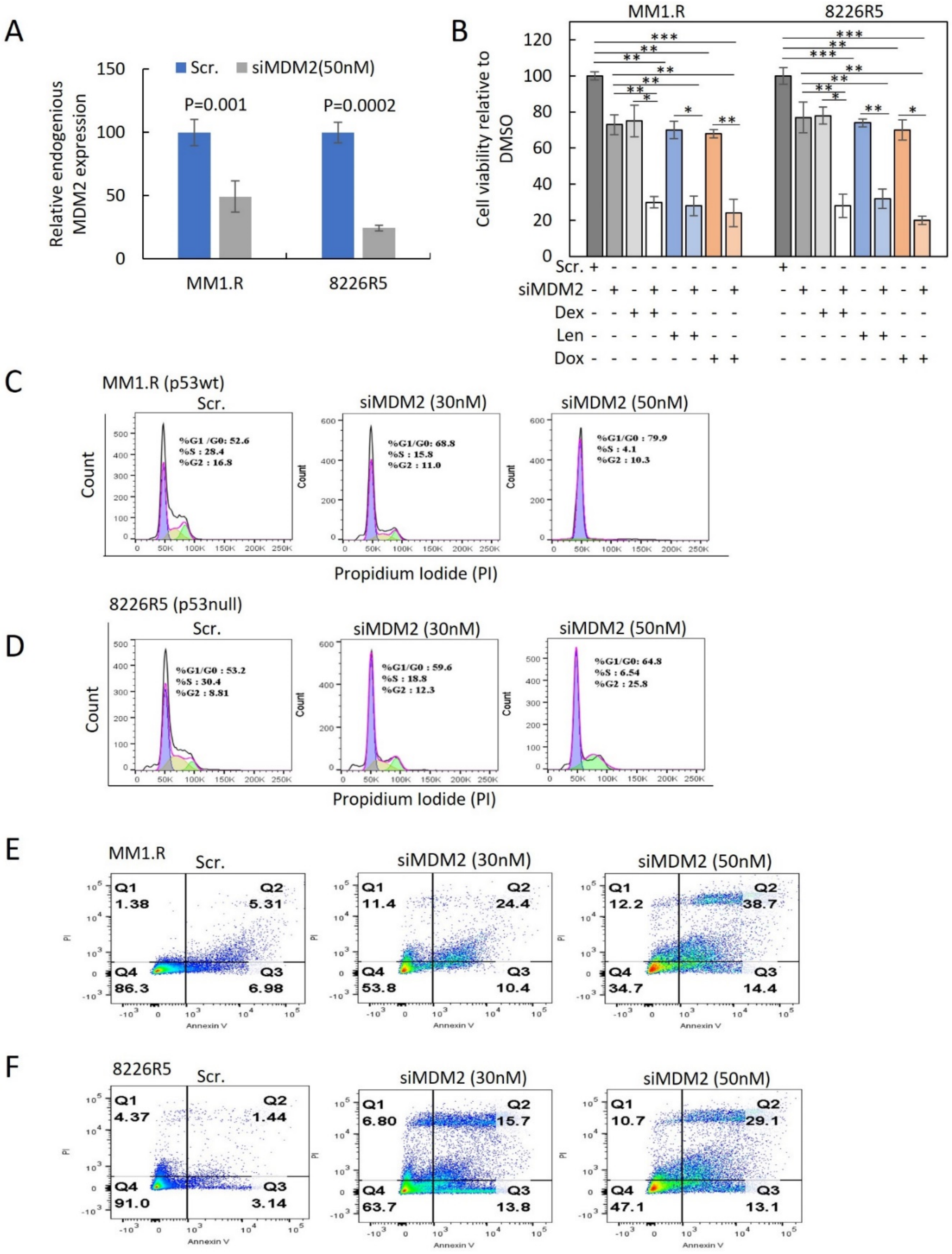

Figure S2. Knockdown of MDM2 induces growth inhibition, apoptosis and cell cycle arrest in drug resistant MM cells. (**A**) MDM2 mRNA was quantified from MM1.R and 8226R5 cells after 48 hr transfection with scrambled siRNA (Scr) or 50nM siMDM2 using qRT-PCR (**B**) Cell viability of MM1.R and 8226R5 cells after 24 hr transfection with scrambled siRNA (Scr) or 10 nM siMDM2 followed by 24 hr treatment with drug (10 µM Dex/5 µM Len/1 µM Dox) or drug vehicle. Cell viability is presented as mean±SD for at least three separate experiments. (**C**,**D**) Representative cell-cycle distribution of MM1.R (**C**) and 8226R5 (**D**) cells after 48 hr transfection with scrambled siRNA (Scr) or different concentrations of siMDM2, as indicated. (**E**,**F**) Apoptosis assay using flow cytometry after staining with Annexin V-FITC and propidium iodide (PI). Annexin V can be detected in both early and late stages of apoptosis, whereas PI can only be detected in late apoptosis

or necrosis. Early apoptotic cells were positive for Annexin V and negative for PI (lower right quadrant); late apoptotic cells were stained for both Annexin V and PI (upper right quadrant). Representative apoptosis analysis of MM1.R (**E**) and 8226R5 (**F**) cells after 48 hr transfection with scrambled siRNA (Scr) or different concentrations of siMDM2, as indicated. *, $p < 0.05$; **, $p < 0.01$, ***, $p < 0.001$.

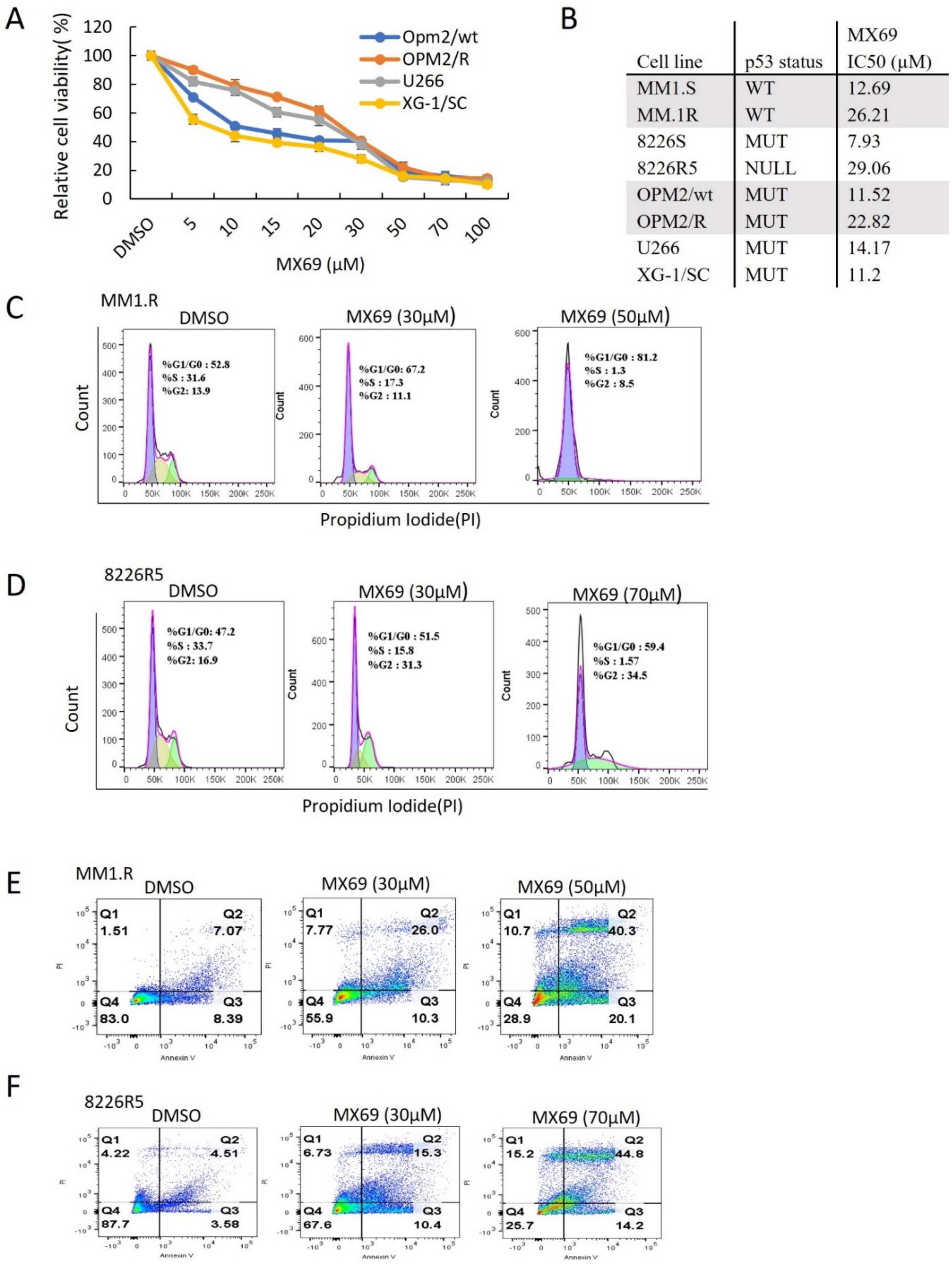

Figure S3. MX69 inhibits MM cell growth, apoptosis and cell cycle arrest in drug-resistant MM cells. (**A**,**B**) Cell viability of MM cell lines after 48 hr treatment with DMSO or different concentrations of MX69, as indicated. Insert - IC50 of MX69 in different MM cell lines, as indicated. Results are represented as the mean±SD of three independent experiments. (**C**,**D**) Representative cell-cycle distribution of MM1.R (**C**) and 8226R5 (**D**) cells after 48 hr treatment with DMSO or different concentrations of MX69. (**E**,**F**) Apoptosis assay using flow cytometry after staining with Annexin V-FITC/propidium iodide. Representative apoptosis analysis of MM1.R (**E**) and 8226R5 (**F**) cells after 48 hr treatment with DMSO or different concentrations of MX69, as indicated.

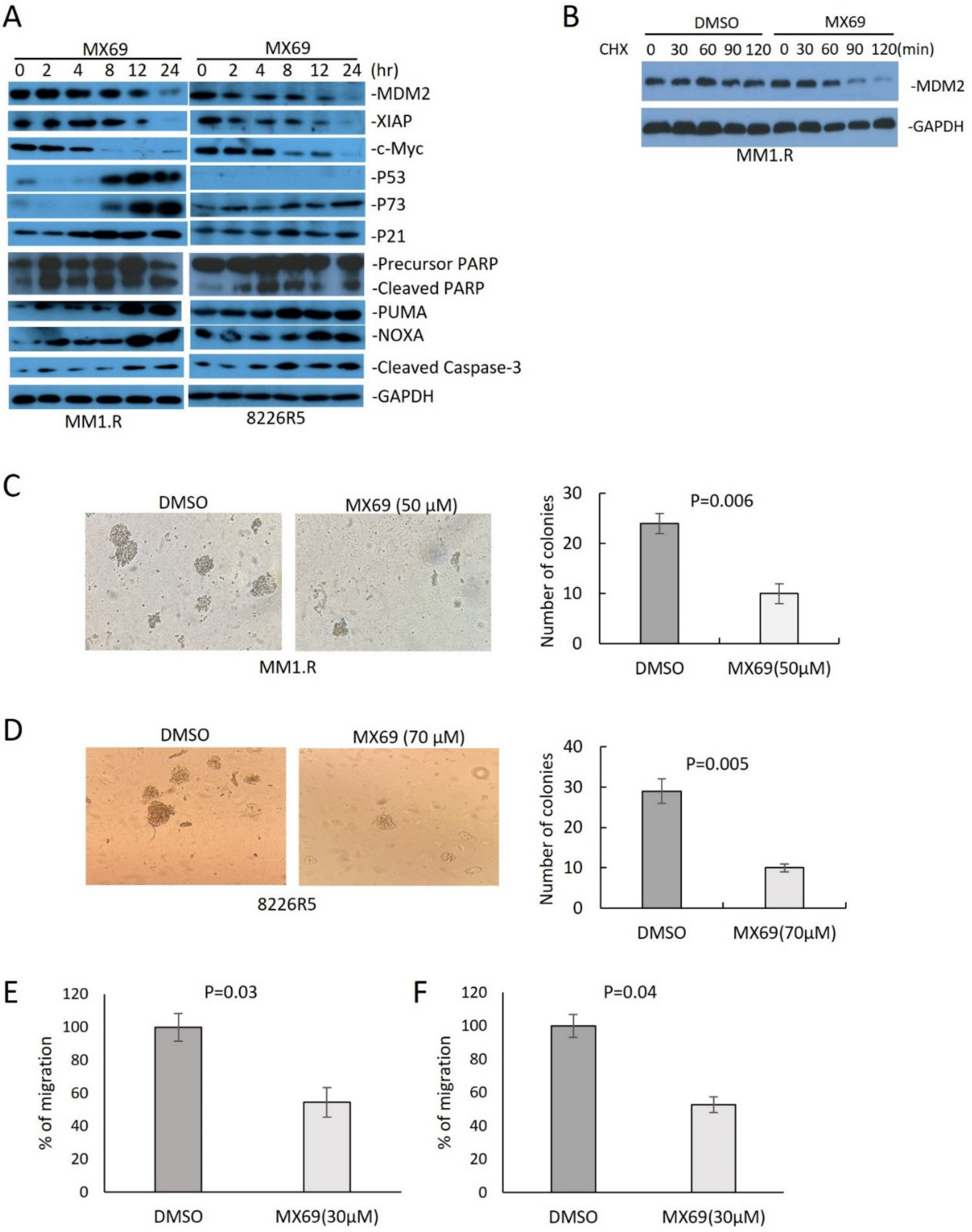

Figure S4. MX69 induces apoptosis and inhibits colony formation and migration in MM. (**A**) Western blot of cell lysates from MM1.R and 8226R5 cells treated with MX69 in time-dependent manner, as indicated. The uncropped blot is in Supplemental Figure S18. (**B**) Western blot of cell lysates from CHX pulse-chase assay for protein turnover in MM1.R cells treated with DMSO or 50 μM MX69 for 4 hr. At the time points indicated after CHX treatment, cell lysates were prepared for

western blot assay. The uncropped blot is in Supplemental Figure S19. (**C,D**) Representative images of soft agar colony-formation assay of MM1.R (**C-left**) and 8226R5 (**D-left**) cells after treatment with DMSO or MX69, as indicated. The image was taken using a microscope set with a 200x magnification (20x objective, 10x eyepiece). Barplots indicate the colony count from different microscopic fields for MM1.R (**C-right**) and 8226R5 (**D-right**). Results are represented as the mean±SD of three independent experiments. (**E,F**) Representative bar graphs quantifying data from migration assay of MM1.R (**E**) and 8226R5 (**F**) cells after treatment with DMSO or MX69, as indicated. Results are represented as the mean±SD of three independent experiments.

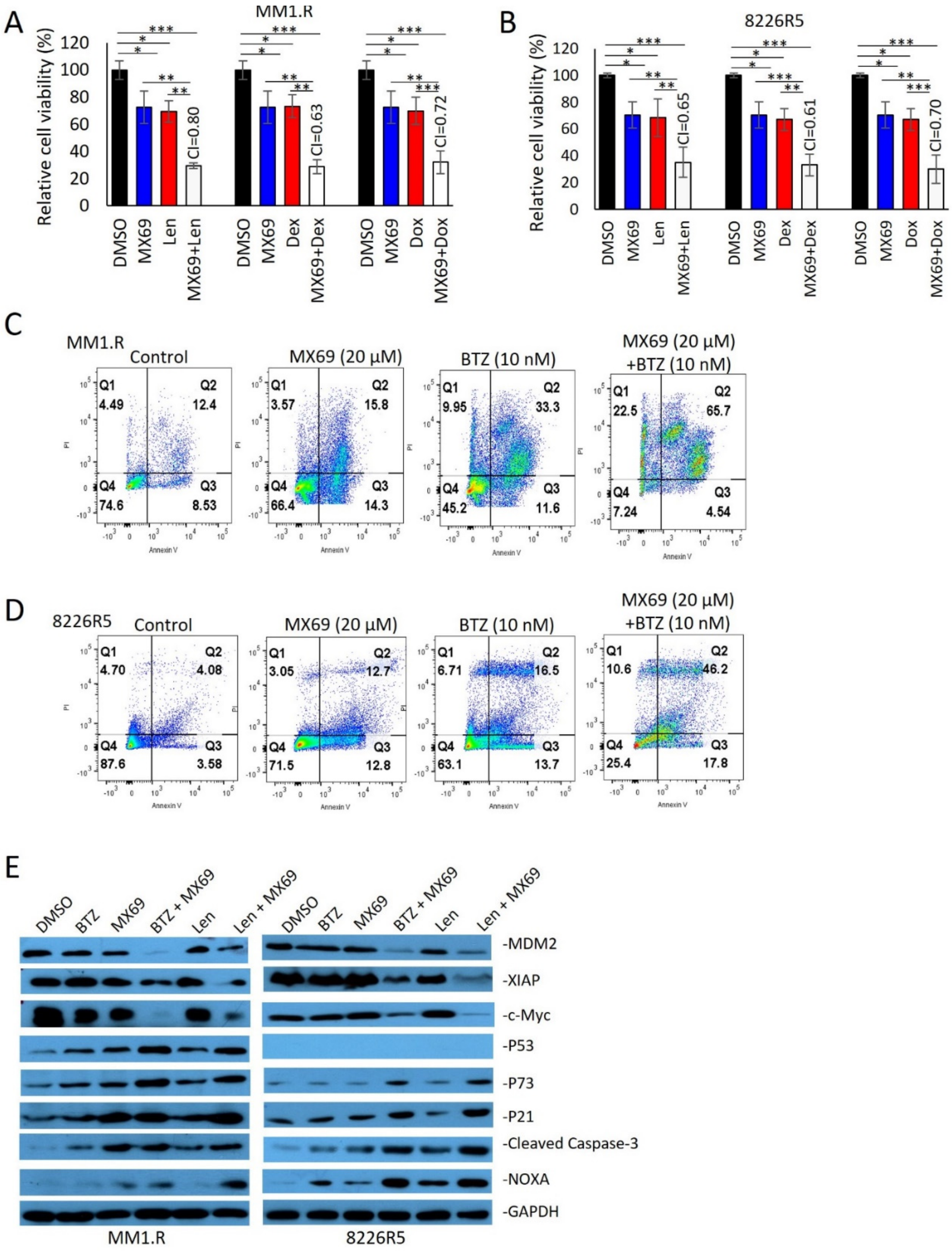

Figure S5. Combination of MX69 with other anti-myeloma drugs show synergistic effects on resistant MM cells. (**A,B**) Cell viability of MM1.R and 8226R5 after 48 hr treatment with DMSO or single/combination treatment with 20 µM MX69, 2.5 µM Dex, 5 µM Len or 1 µM Dox. Results are

represented as the mean±SD of three independent experiments. (**C**,**D**) Apoptosis assay of MM1.R and 8226R5 cells after staining with Annexin V-FITC/propidium iodide. Representative apoptosis analysis of MM1.R (**C**) and 8226R5 (**D**) cells after 48 hr treatment with DMSO or single/combination treatment with MX69 and BTZ, as indicated. (**E**) Western blot of cell lysates from MM1.R (left) and 8226R5 (right) cells after 24 hr treatment with DMSO (NC) or single/combination treatment with 20μM MX69, 5nM BTZ, 5 μM Len, as indicated. The uncropped blot is in Supplemental Figure S20. *, $p < 0.05$; **, $p < 0.01$, ***, $p < 0.001$.

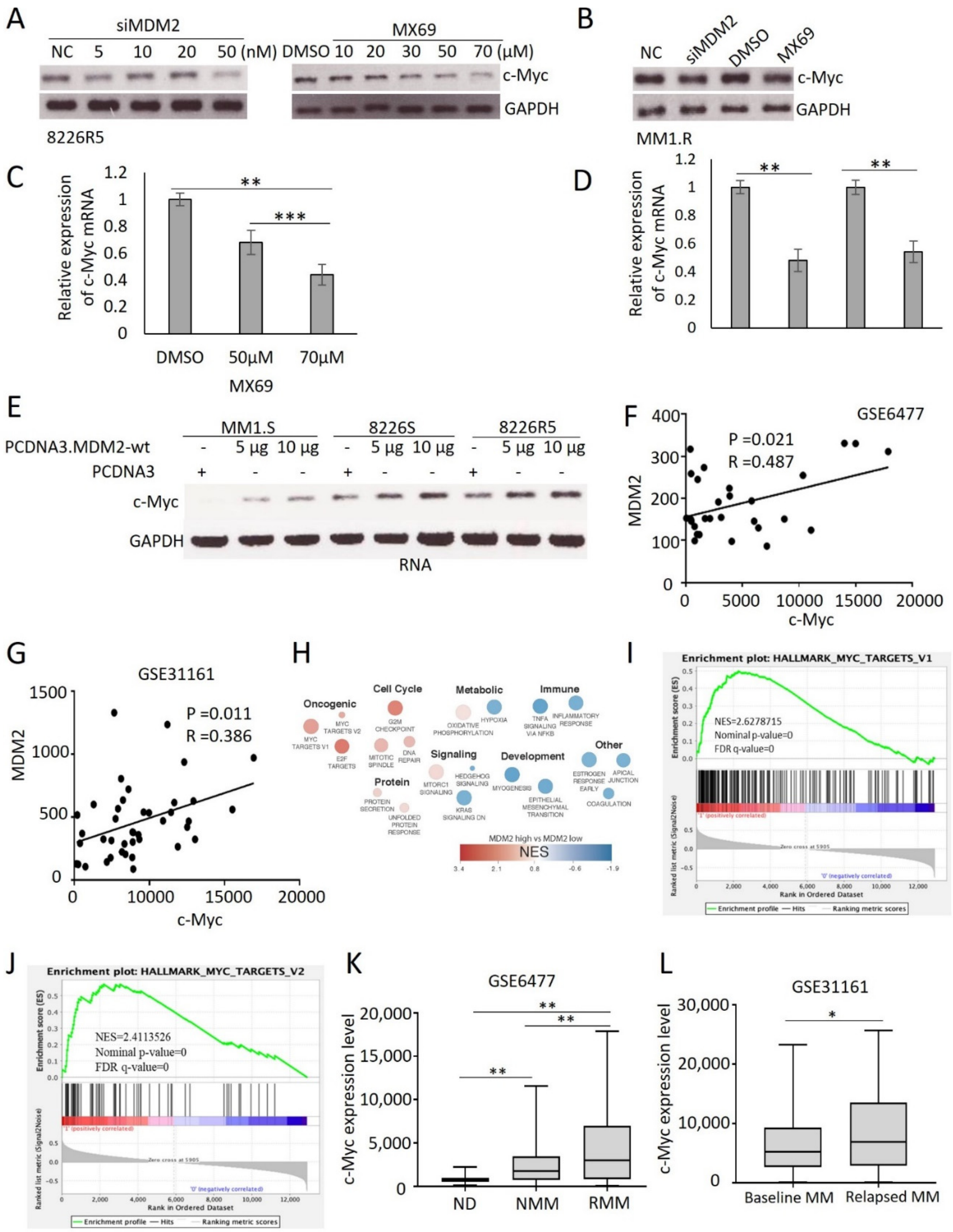

Figure S6. c-Myc and MDM2 expressions are positively correlated in resistant MM cells. (**A**–**D**) Traditional PCR and quantitative PCR results indicating the levels of c-Myc and GAPDH mRNA expression in (**A**,**C**) 8226R5 or (**B**,**D**) MM1R cells after 48 hr transfection with siMDM2 and control siRNA(Scr.) or DMSO and MX69 with indicated concentrations. Results are represented as the

mean±SD of three independent experiments. (**E**) Traditional PCR results indicating the levels of c-Myc mRNA in MM1.S, 8226S and 8226R5 cells after transfection with MDM2-WT or control plasmid for 48 hr. (**F**,**G**) Scatter plot of MDM2 and c-Myc expression in relapsed MM patients (GSE6477, n=28; GSE31161, n=100). Linear regression with Pearson's correlation coefficients (r) and p-values are indicated. (**H**) Gene set enrichment analysis (GSEA) according to MDM2 mRNA expression in relapsed MM (GSE6477). (**I**,**J**) Enrichment plots of MYC_TARGETS_V1 pathway and MYC_TARGETS_V2 pathways. (**K**,**L**) c-Myc expression in different stages of MM (GSE6477, ND n=15, newly diagnosed MM (NMM) n=73, relapsed MM (RMM); GSE31161, baseline MM n=100, relapsed MM n=100). *, $p < 0.05$; **, $p < 0.01$, ***, $p < 0.001$.

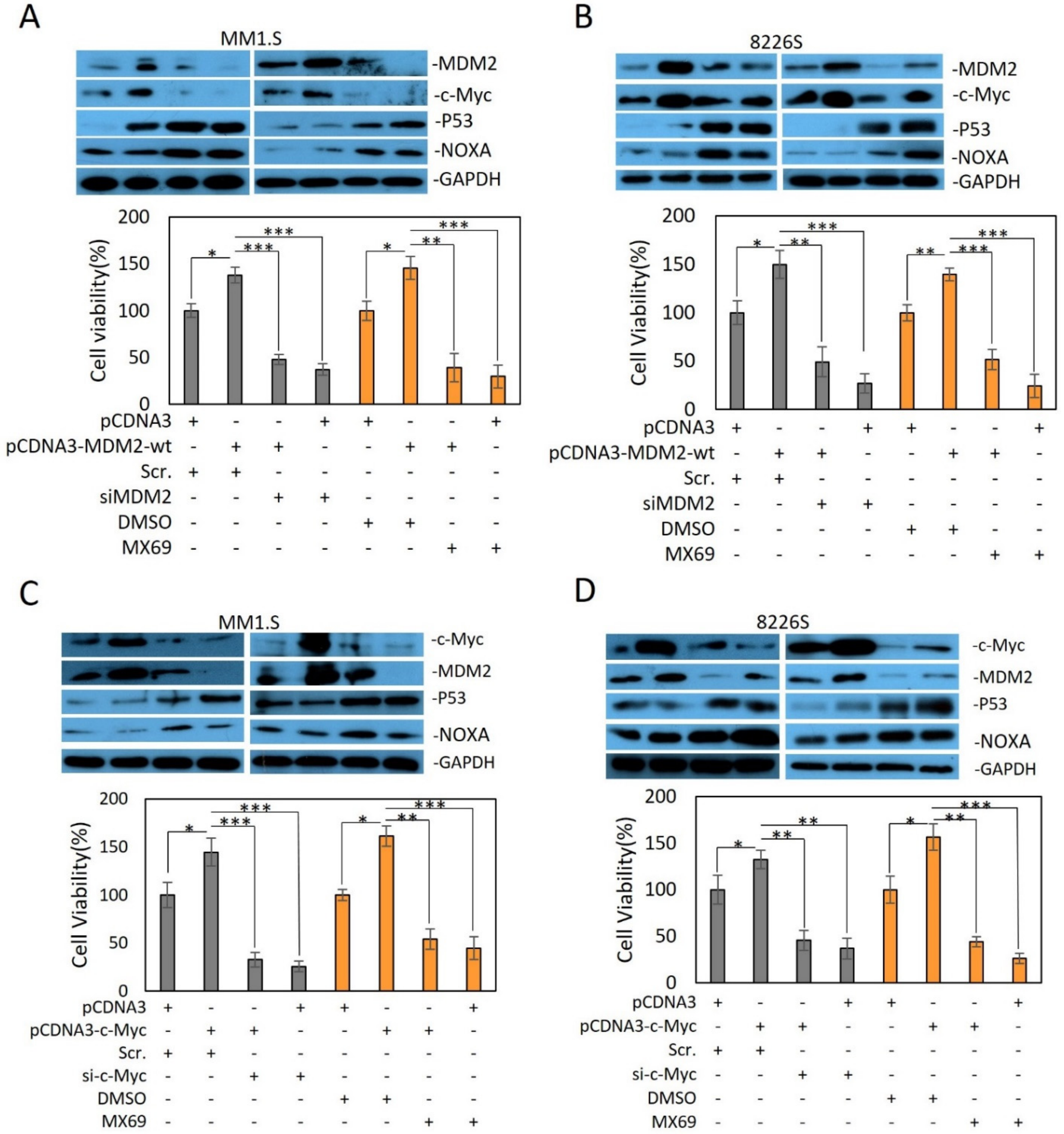

Figure S7. MDM2 overexpression reciprocally induces overexpression of c-Myc. (**A**,**B**) Western blot of cell lysates and cell viability of MM1.S and 8226S cells after 48 hr transient transfection with 5 µg MDM2-wt plasmid or control plasmid with 20 nM siMDM2 or scrambled siRNA (**A**) and after 24 hr transient transfection with 5 µg MDM2-wt plasmid or control plasmid followed by 24 hr treatment with 20 µM MX69 or drug vehicle (**B**). Cell viability results are represented as mean±SD for at least three independent experiments. (**C**,**D**) Western blot of cell lysates and cell

viability of MM1.S and 8226S cells after 48 hr transient transfection with 5 µg c-Myc plasmid or control plasmid with 20 nM si-cMyc or scrambled siRNA (**C**) and after 24 hr transient transfection with 5 µg c-Myc plasmid or control plasmid followed by 24 hr treatment with 20 µM MX69 or drug vehicle (**D**). Cell viability results are represented as mean±SD for at least three independent experiments. The uncropped blot is in Supplemental Figures S21–24. *, $p < 0.05$; **, $p < 0.01$, ***, $p < 0.001$.

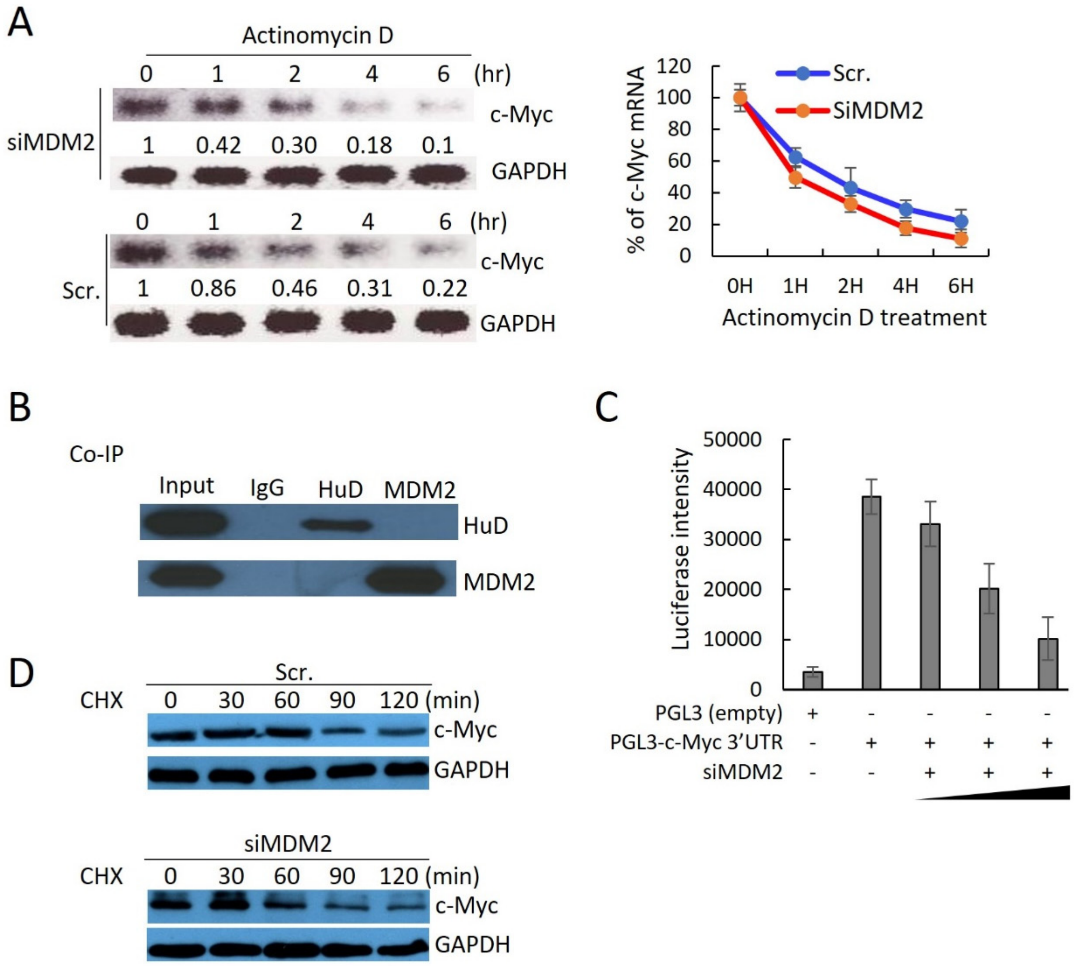

Figure S8. Effect of MDM2 KD on c-Myc expression. (**A**) 8226R5 cells were treated either with MDM2 siRNA or a control siRNA, then were treated with 5 mg/mL actinomycin-D. At different time points after addition of actinomycin-D the cells were harvested, and then total RNA was isolated. The amount of c-Myc mRNA in 8226R5 cells was determined by PCR and quantified by densitometric analysis. The quantified c-Myc mRNA levels were normalized to GAPDH, and the normalized c-Myc mRNA levels were compared to the samples corresponding to 0 hours. Numerical labels under the bands in the blot represent the ratio of normalized c-Myc mRNA levels. Line graphs represent the qRT-PCR of the same experiment. Line graphs represent the mean±SD of three independent experiments. (**B**) Western blot of cell lysates from MM1.R were immunoprecipitated (IP) using indicated antibodies. (**C**) 8226R5 cells were transfected with 5ug of pGL3-c-Myc 3′UTR plasmids with or without increasing amounts (20, 30, 50 nM) of siMDM2. After 48 hr, cell extracts were prepared and firefly luciferase activity (pGL3-c-Myc 3′UTR) was detected with the Dual Luciferase Reporter System. Controls included transfection of pRL empty vector alone. Quantitative renila luciferase and firefly luciferase activities were detected using the Dual Luciferase Reporter System. Results are represented as the mean±SD of firefly luciferase in three independent experiments, normalized to renila luciferase activity. (**D**) Western blot of cell lysates from CHX pulse-chase assay for protein turnover in MM1.R cells after 48 hr transfection with siMDM2 or control siRNA. At the time points indicated after CHX treatment, cell lysates were prepared for western blot assay. The uncropped blot is in Supplemental Figure S25.

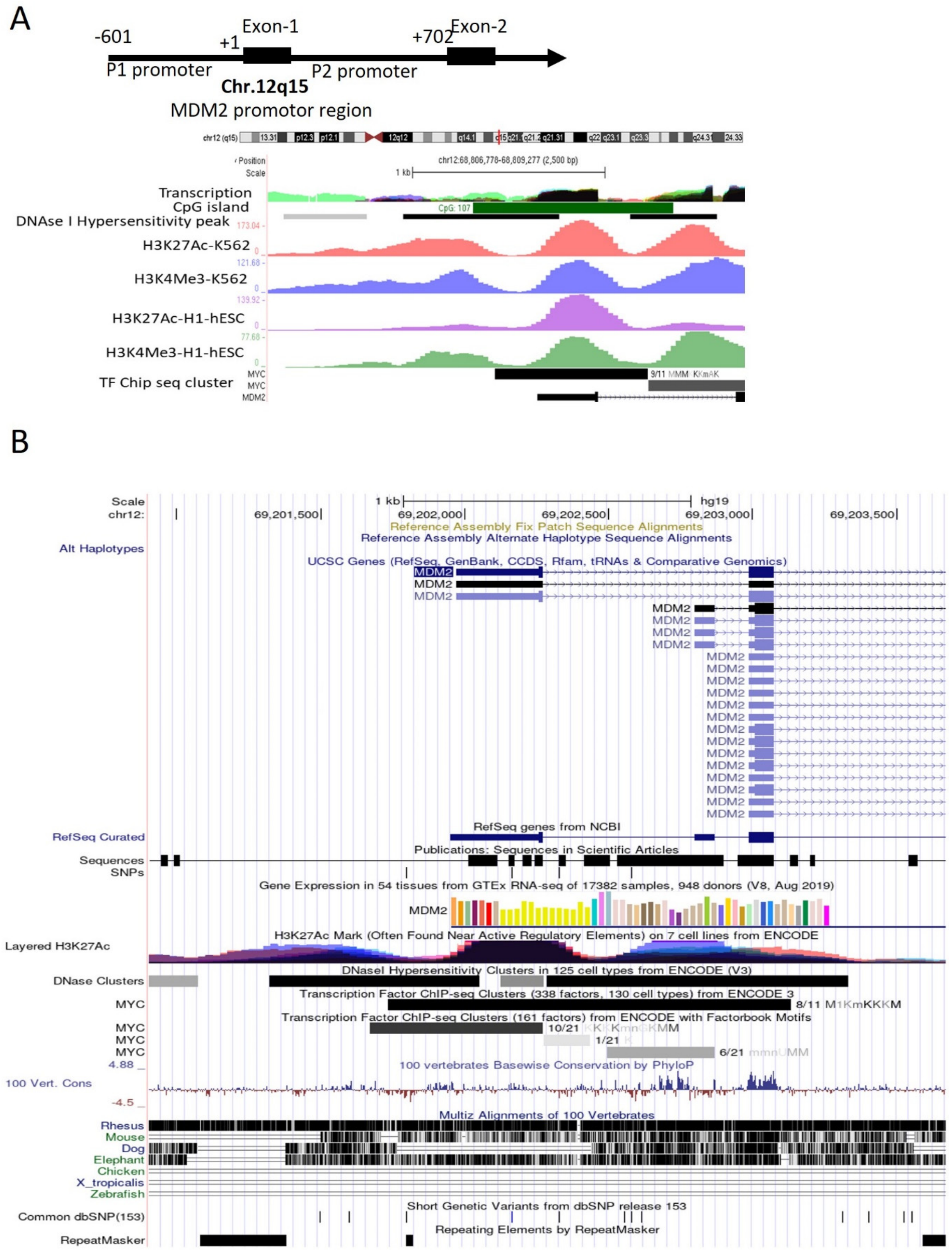

Figure S9. C-Myc transcription factor binding site on the MDM2 promoter regions. (**A**) Schematic diagram of the human MDM2 promoter region 1 and 2. c-Myc binding sties and histone modification around the MDM2 promoter identified by Chip-seq data from ENCODE. Genome-wide identification of transcription binding sites of c-Myc in the MDM2 promoter region was performed with UCSC data. Transcription levels assayed by RNA-seq and histone modification (H3K4me3 and H3K9ac) ChIP-seq on MDM2 in K562 and H1-hESC cells. Plots are aligned to the

transcript representations. CpG islands are indicated by green boxes. (**B**) Genome-wide identification of transcription binding sites of c-Myc in the MDM2 promoter region was performed with ENCODE.

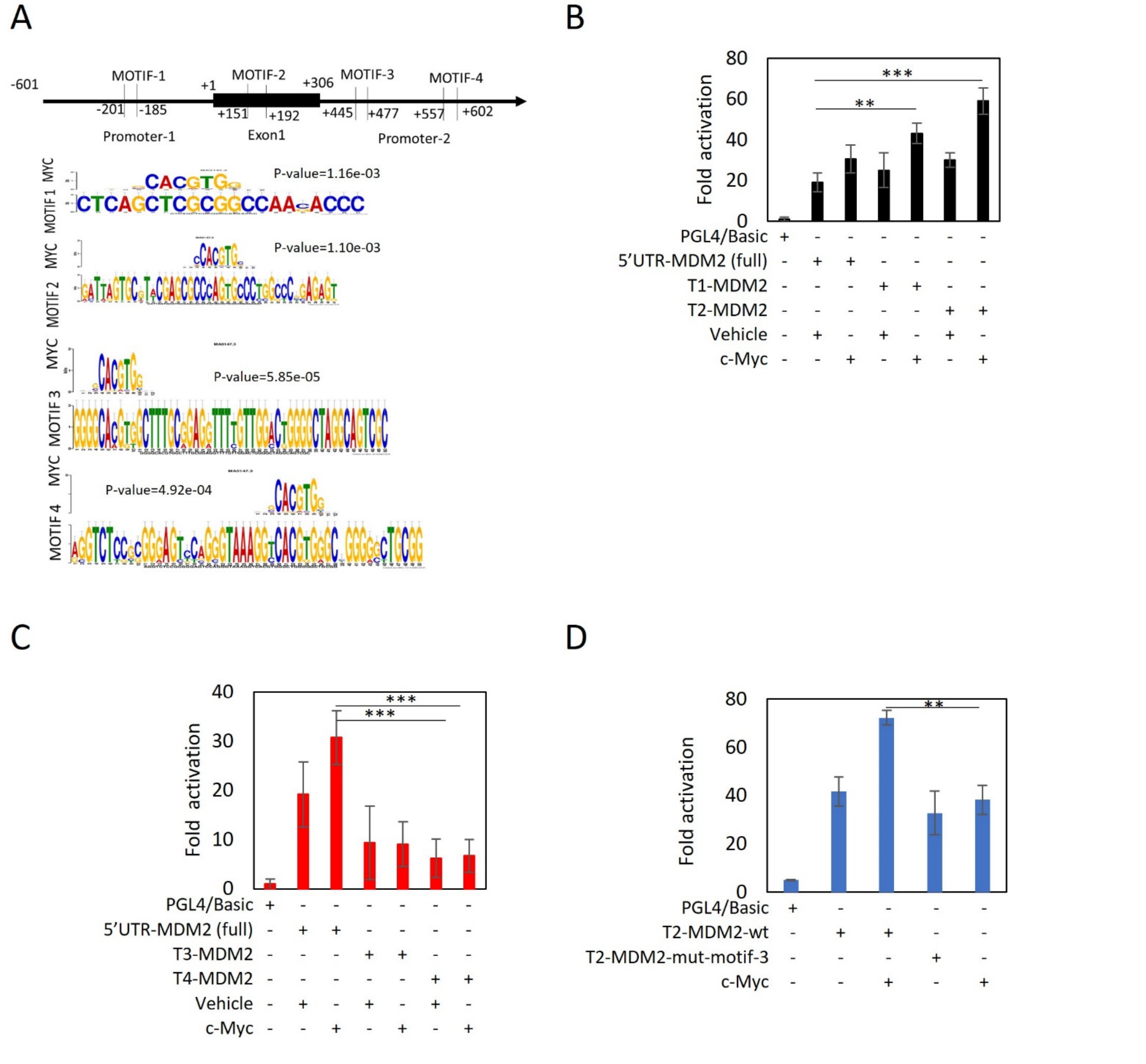

Figure S10. MDM2 is a direct transcriptional target of c-Myc. (**A**) Tomtom analysis results for conserved motifs for c-Myc binding prediction. c-Myc transcription factor predicted for four consensus sequences (as query motif) by Tomtom analysis out of 40 motifs. Selected set of DHS overlapped motif aligning with their TF's PWM (top) and query motif (bottom) with binding specificity indicated by p values. Schematic diagram of the motif positions on the human MDM2 promoter region 1 and 2. (**B**) Cells were transfected with T1MDM2 5′UTR and T2MDM2 5′UTR. Values shown are fold activation over the empty vector control (mean±SE for three independent experiments: n = 9). (**C**) Results of transfection experiments using T3MDM2 5′UTR and T4MDM2 5′UTR. Values shown are fold activation over the empty vector control (mean±SE for three independent experiments: n = 9). (**D**) 293T cells were transfected with 5 ug of T2MDM2 5′UTR (wt or mutant c-Myc E-Box sequence) plasmids promoter with or without increasing amounts 200ng of c-Myc or pcDNA 3.1-control plasmids. After 48 hr, cell extracts were prepared, and firefly luciferase activity was detected with the Dual Luciferase Reporter System. Values shown are fold activation

over the empty vector control (mean±SE for three independent experiments: n = 9). **, $p < 0.01$, ***, $p < 0.001$.

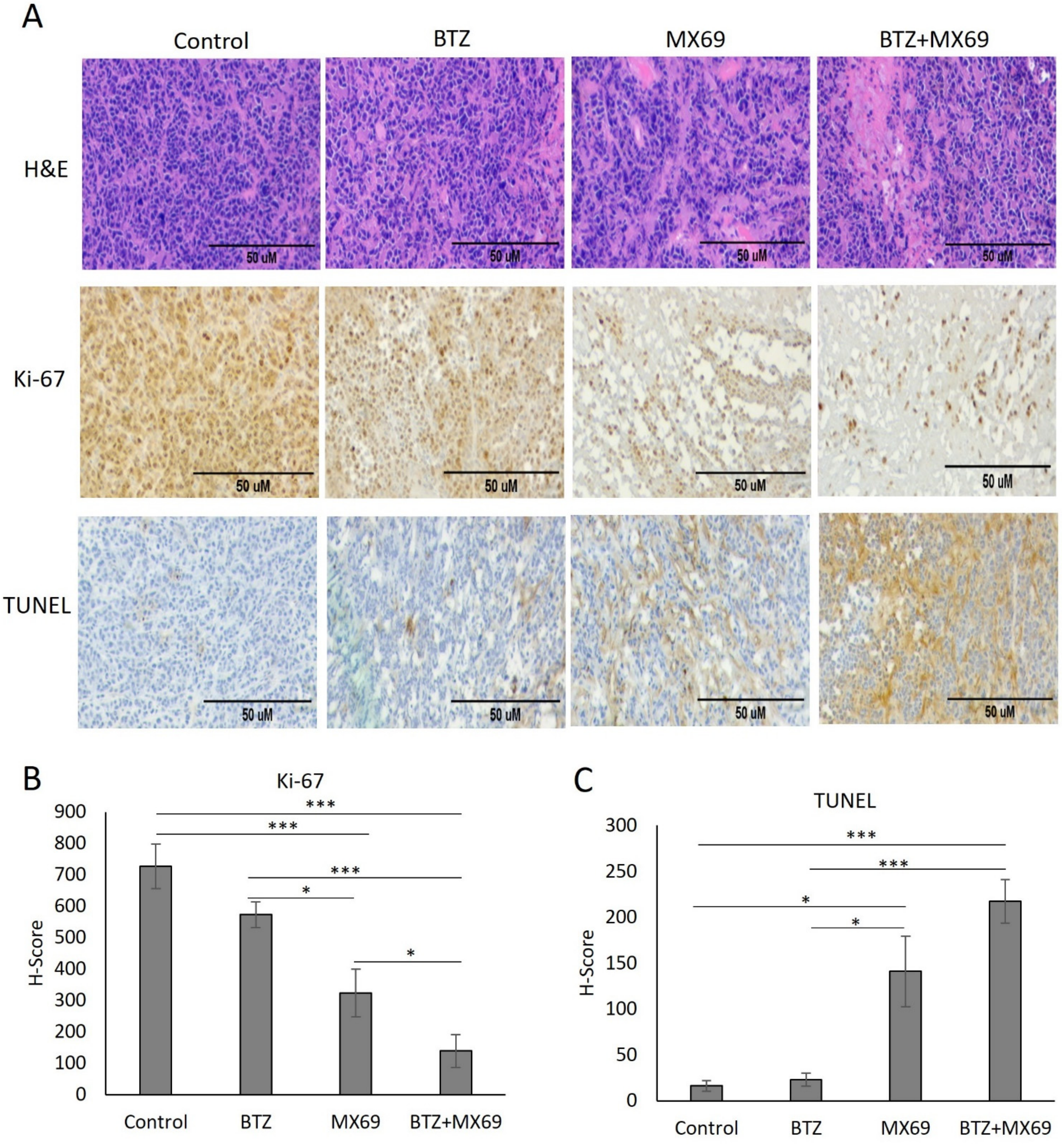

Figure S11. Immunohistochemistry of tumor sections from mice treated with control, BTZ, MX69 and BTZ+MX69 and respective scoring. (**A**) Representative microscopic image (Magnification, x50) of tumor sections for four treated groups analyzed for histology (H&E), proliferation (Ki-67) or apoptosis (TUNEL). (**B**,**C**) Representative expressions of Ki-67 and TUNEL markers studied by IHC H-score. In the bar graphs, plots show that spots marker as the H-score. *, $p < 0.05$; ***, $p < 0.001$.

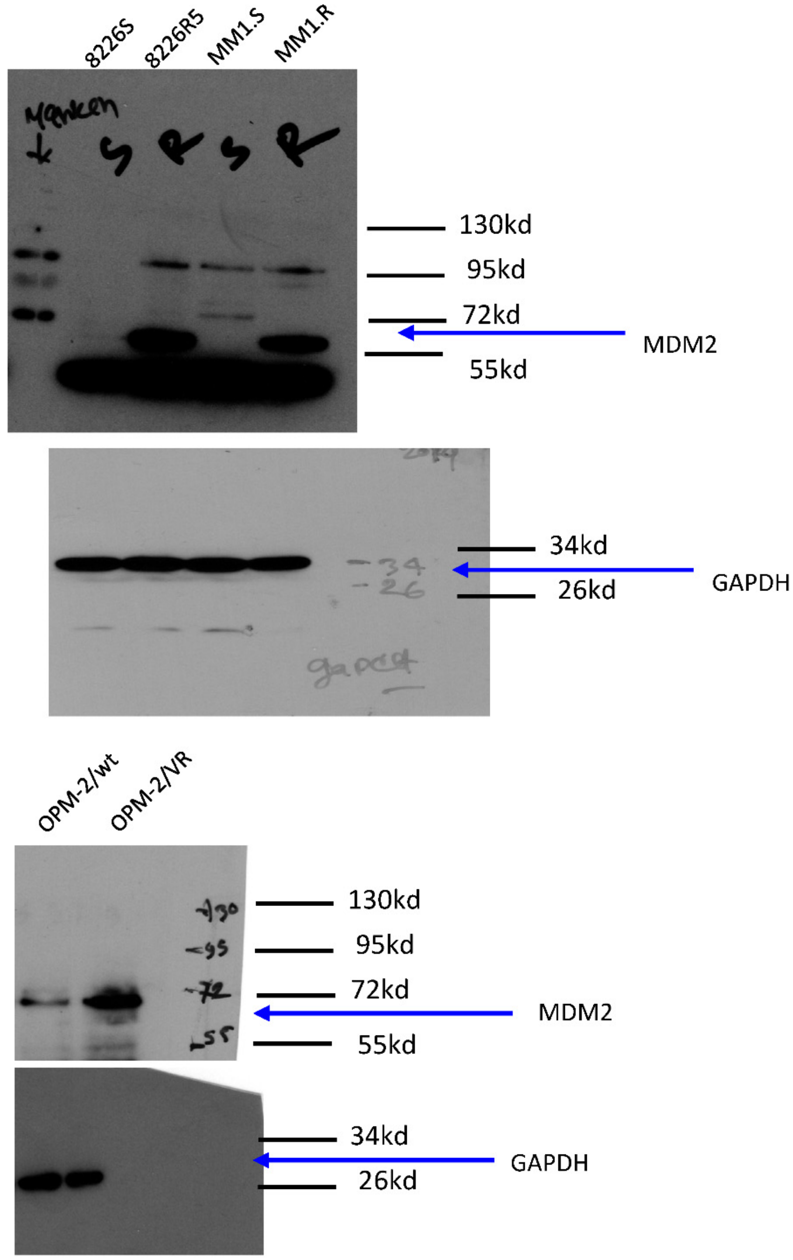

MDM2 protein Intensity ratio (GAPDH was used as normalization standard) was measured compare with resistance cells

Cells Name	8226S	8226R5	MM1.S	MM1.R	OPM-2/wt	OPM-2/VR
MDM2 Intensity ratio	0	1	0	1	0.19	1

Figure S12. Uncropped blots for Figure 1F.

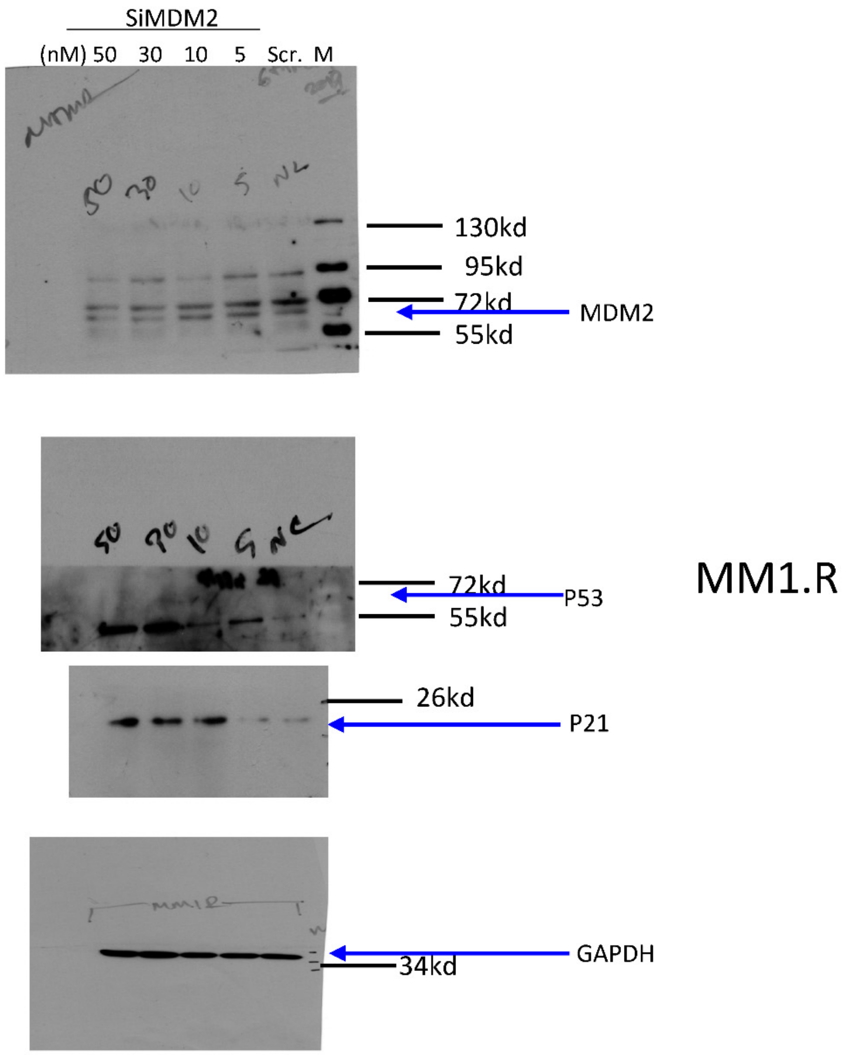

MDM2, and (P53 and P21) proteins Intensity ratio (GAPDH was used as normalization standard) were calculated respective treatment with Scramble siRNA and 50nM si-MDM2.

siMDM2	Scr.	5nM	10nM	30nM	50nM
MDM2	1	0.87	0.66	0.31	0.3
P53	0.3	0.35	0.31	1.1	1
P21	0.22	0.23	0.63	0.79	1

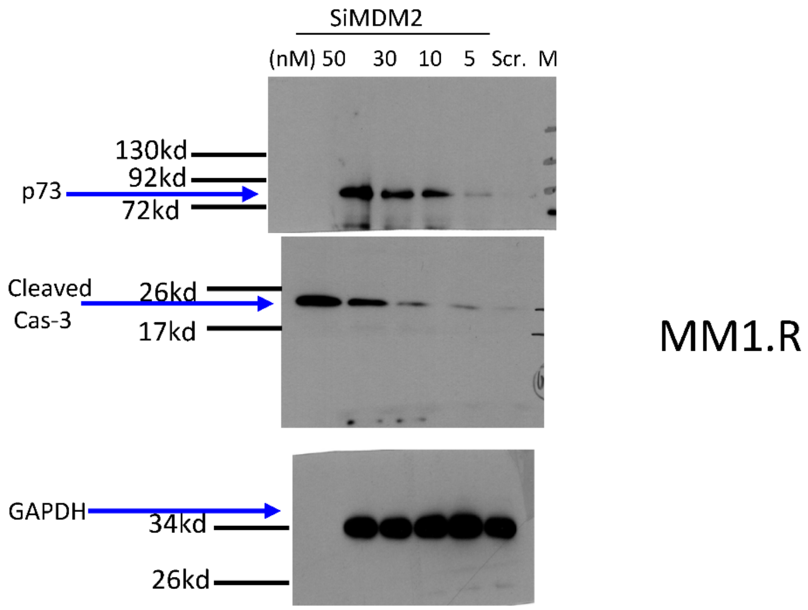

P73 and Cleaved Cas-3 proteins Intensity ratio (GAPDH was used as normalization standard) were calculated respective transfection with 50nM si-MDM2.

siMDM2	Scr.	5nM	10nM	30nM	50nM
P73	0.07	0.08	0.47	0.6	1
Cleaved Cas-3	0.15	0.16	0.36	0.67	1

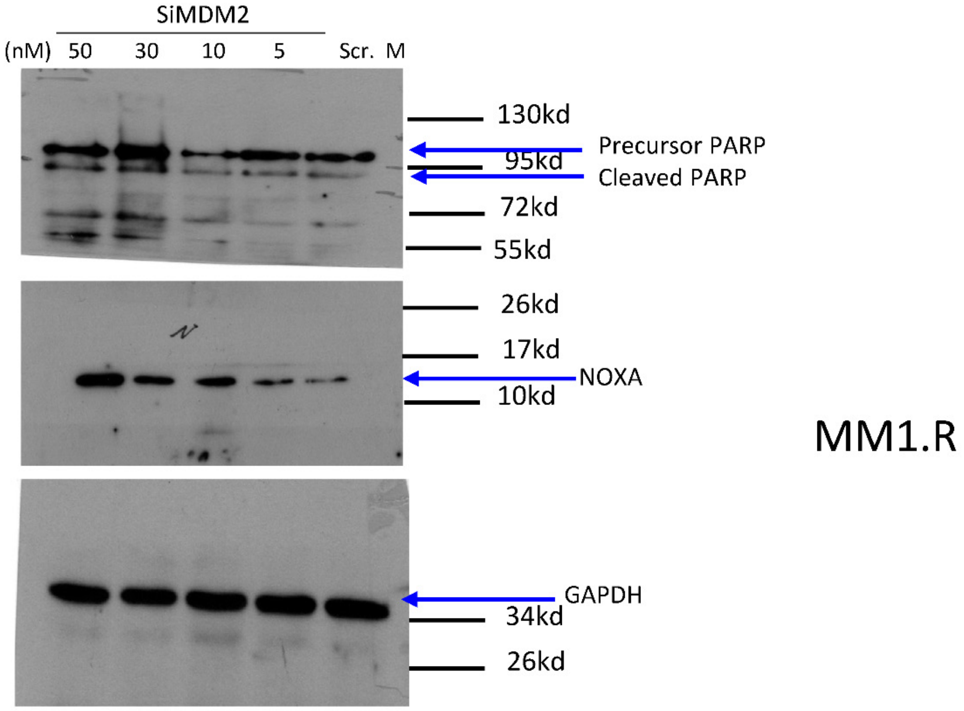

Cleaved PARP and NOXA proteins Intensity ratio (GAPDH was used as normalization standard) were calculated respective transfection with 50nM si-MDM2.

siMDM2	Scr.	5nM	10nM	30nM	50nM
Cleaved PARP	0.38	0.39	0.38	1.06	1
NOXA	0.07	0.08	0.47	0.63	1

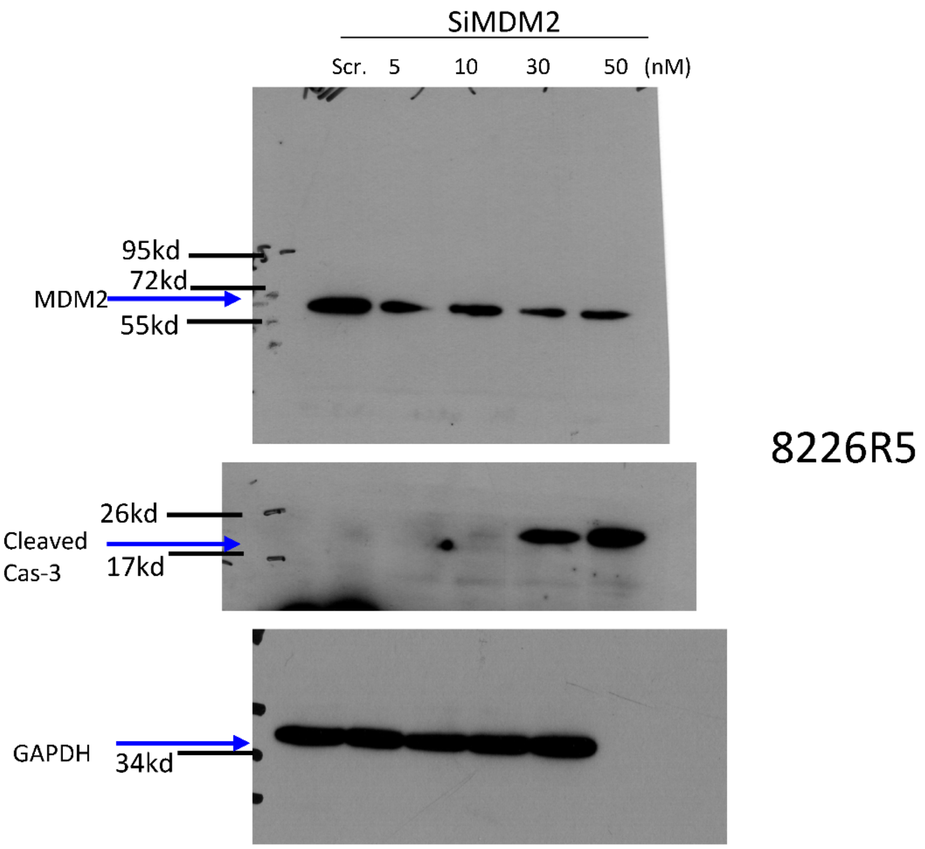

MDM2 and Cleaved Cas-3 proteins Intensity ratio (GAPDH was used as normalization standard) were calculated respective transfection with Scramble siRNA and 50nM si-MDM2 .

siMDM2	Scr.	5nM	10nM	30nM	50nM
MDM2	1	0.92	0.89	0.46	0.32
Cleaved Cas-3	0.22	0.24	0.26	0.96	1

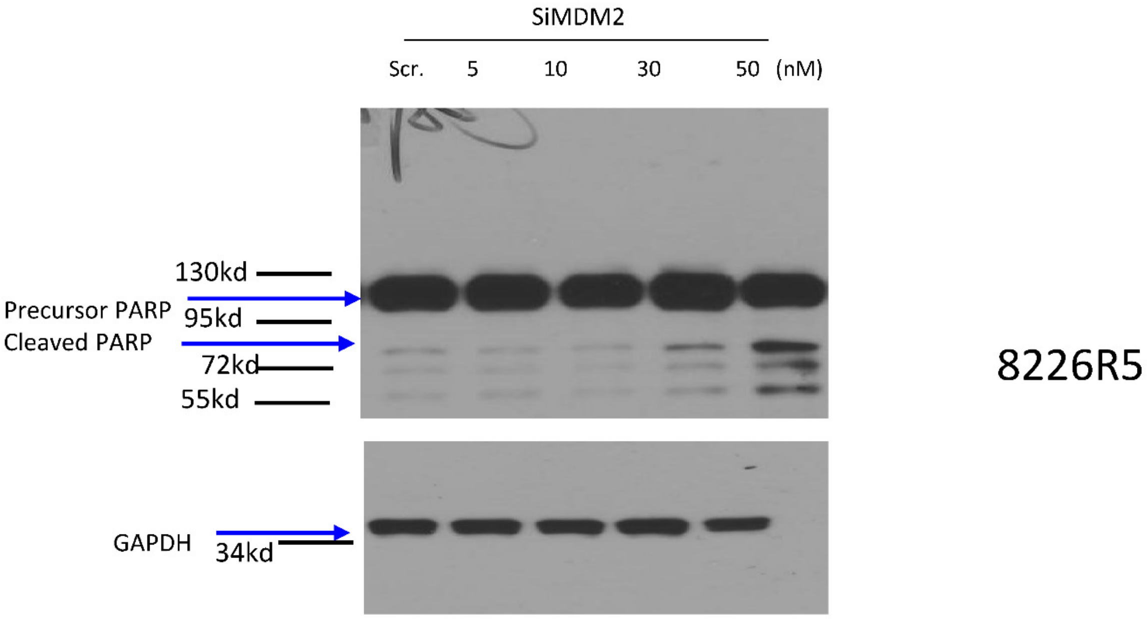

Cleaved PARP protein Intensity ratio (GAPDH was used as normalization standard) was calculated respective transfection with 50nM si-MDM2.

siMDM2	Scr.	5nM	10nM	30nM	50nM
Cleaved PARP	0.35	0.36	0.37	0.76	1

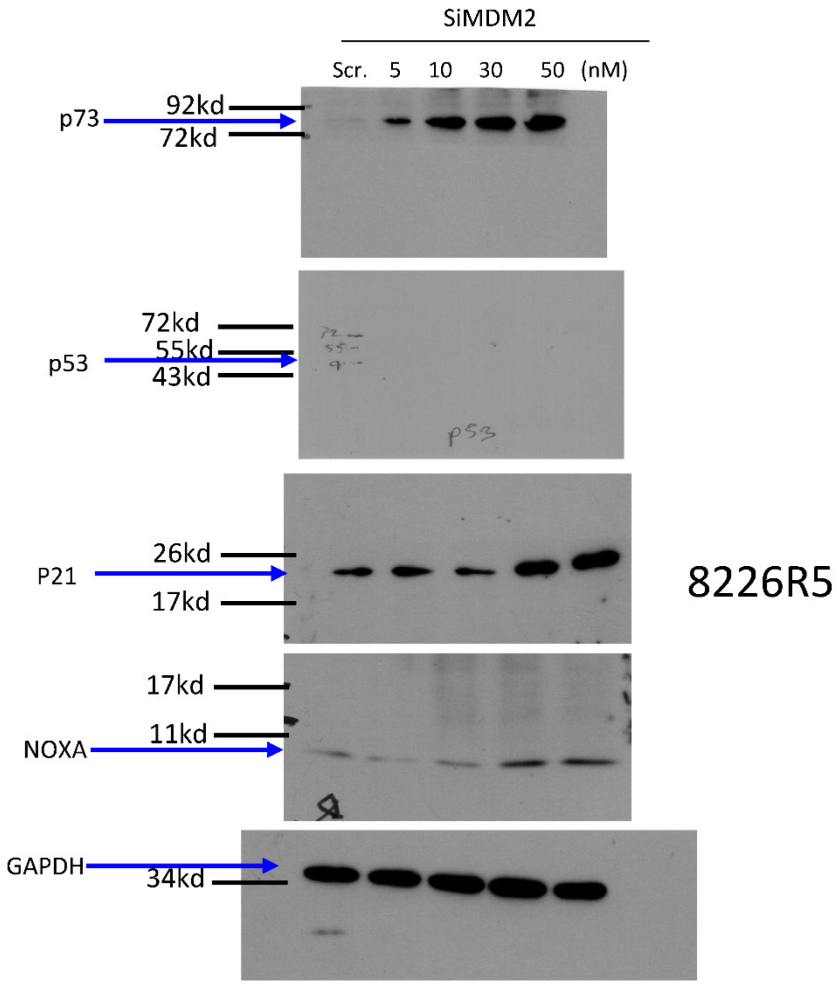

P53, P21 and NOXA proteins Intensity ratio (GAPDH was used as normalization standard) were calculated respective transfection with 50nM si-MDM2 .

siMDM2	Scr.	5nM	10nM	30nM	50nM
P73	0.13	0.38	0.81	0.9	1
P21	0.41	0.4	0.42	0.87	1
Noxa	0.35	0.36	0.41	0.89	1

Figure S13. Uncropped blots for Figure 2D.

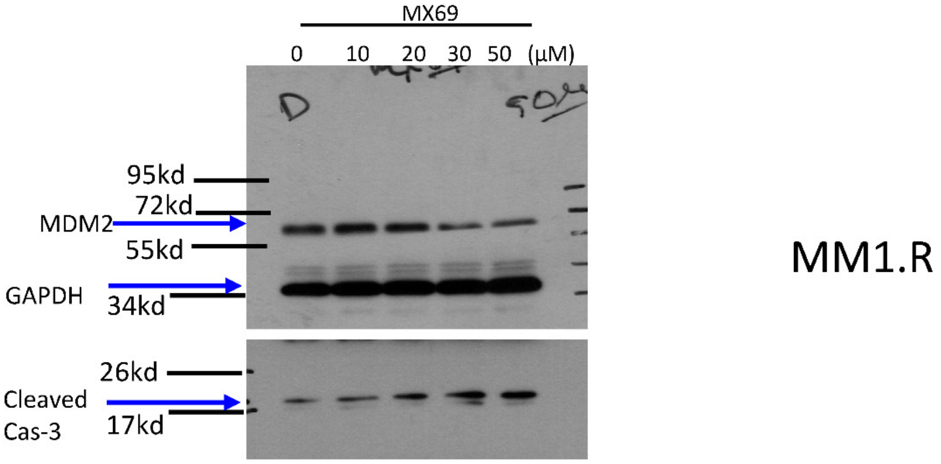

MDM2 and Cleaved Cas-3 Intensity ratio (GAPDH was used as normalization standard)calculated respective treatment with DMSO and 50 µM MX69.

MX69	DMSO	10µM	20 µM	30 µM	50 µM
MDM2	1	0.87	0.88	0.36	0.25
Cleaved Cas-3	0.32	0.31	0.47	0.72	1

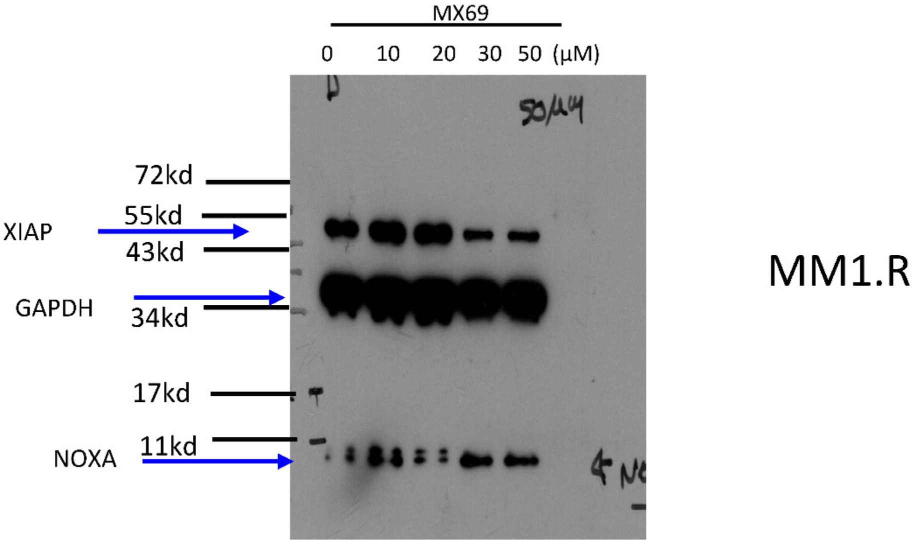

XIAP and NOXA proteins Intensity ratio (GAPDH was used as normalization standard) were calculated respective treatment with DMSO and 50 µM MX69..

MX69	DMSO	10µM	20 µM	30 µM	50 µM
XIAP	1	0.97	0.98	0.32	0.27
NOXA	0.34	0.6	0.62	1.04	1

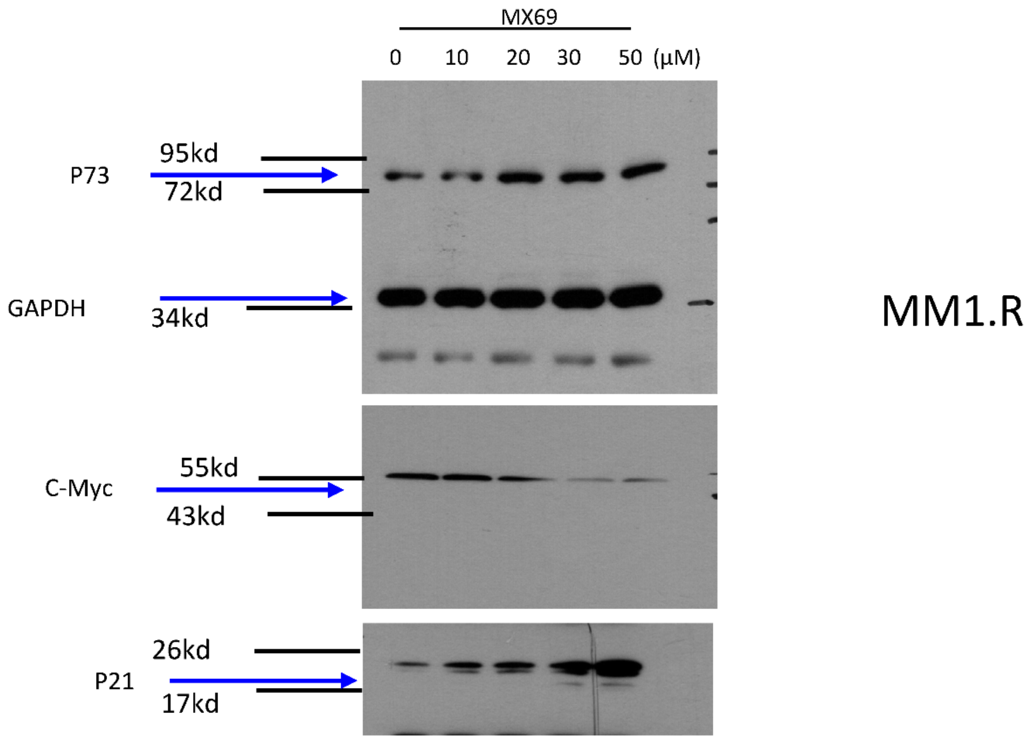

P73, P21 and C-Myc proteins Intensity ratio (GAPDH was used as normalization standard) were calculated respective treatment with 50 µM MX69 and DMSO.

MX69	DMSO	10µM	20 µM	30 µM	50 µM
P73	0.37	0.35	0.91	1.01	1
c-Myc	1	0.98	0.62	0.38	0.29
P21	0.11	0.13	0.21	0.56	1

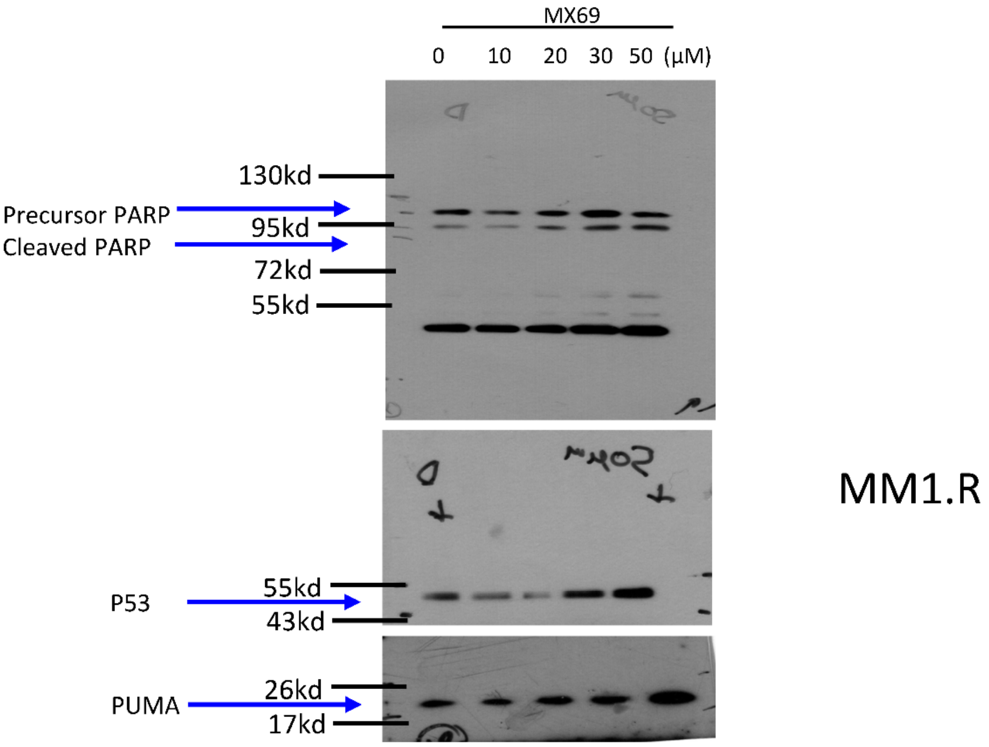

Cleaved PARP and P53 and PUMA proteins Intensity ratio (GAPDH was used as normalization standard) were calculated respective treatment with 50 µM MX69.

MX69	DMSO	10µM	20 µM	30 µM	50 µM
Cleaved PARP	0.39	0.35	0.63	0.95	1
P53	0.22	0.2	0.24	0.73	1
PUMA	0.39	0.41	0.5	0.77	1

8226R5

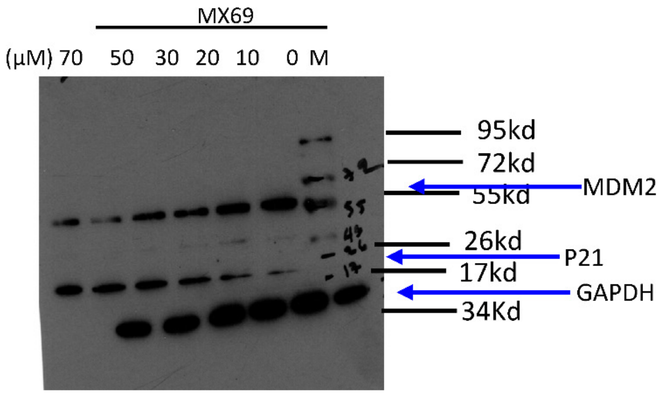

MDM2 and P21 proteins Intensity ratio (GAPDH was used as normalization standard) were calculated respective treatment with DMSO and 70 µM MX69

.

MX69	DMSO	10µM	20 µM	30 µM	50 µM	70 µM
MDM2	1	0.82	0.83	0.66	0.33	0.36
P21	0.25	0.22	0.32	0.74	0.97	1

8226R5

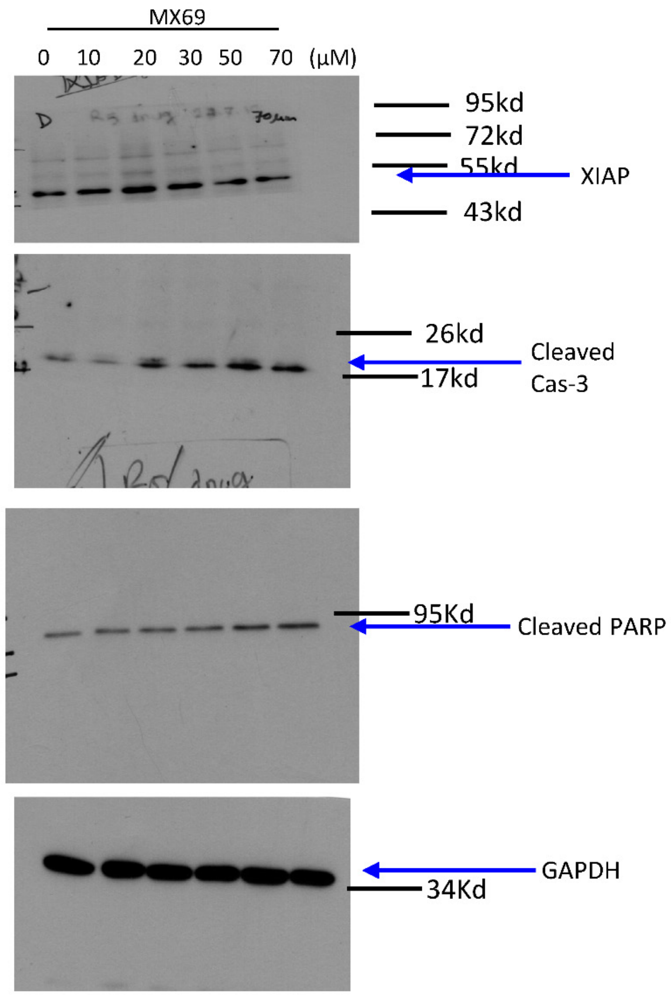

XIAP, Cleaved Cas-3 and Cleaved PARP proteins Intensity ratio (GAPDH was used as normalization standard) were calculated respective treatment with DMSO and 70 μM MX69.

MX69	DMSO	10μM	20 μM	30 μM	50 μM	70 μM
XIAP	1	0.97	1.07	1.04	0.45	0.4
Cleaved Cas-3	0.41	0.34	0.64	0.72	1.04	1
Cleaved PARP	0.5	0.56	0.63	0.66	0.93	1

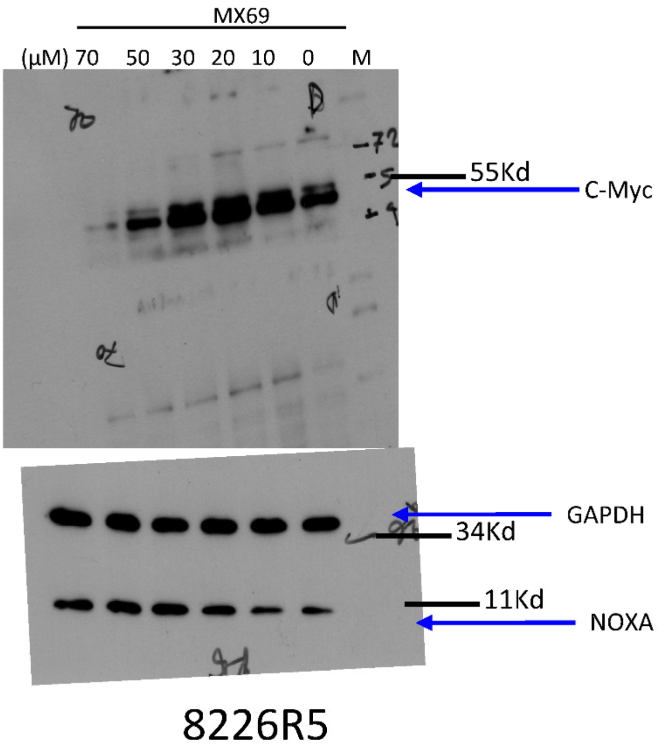

c-Myc and NOXA proteins Intensity ratio (GAPDH was used as normalization standard) were calculated respective treatment with DMSO and 70 µM MX69.

MX69	DMSO	10µM	20 µM	30 µM	50 µM	70 µM
C-Myc	1	1.06	1.14	1.05	0.5	0.26
NOXA	0.33	0.31	0.86	0.94	0.95	1

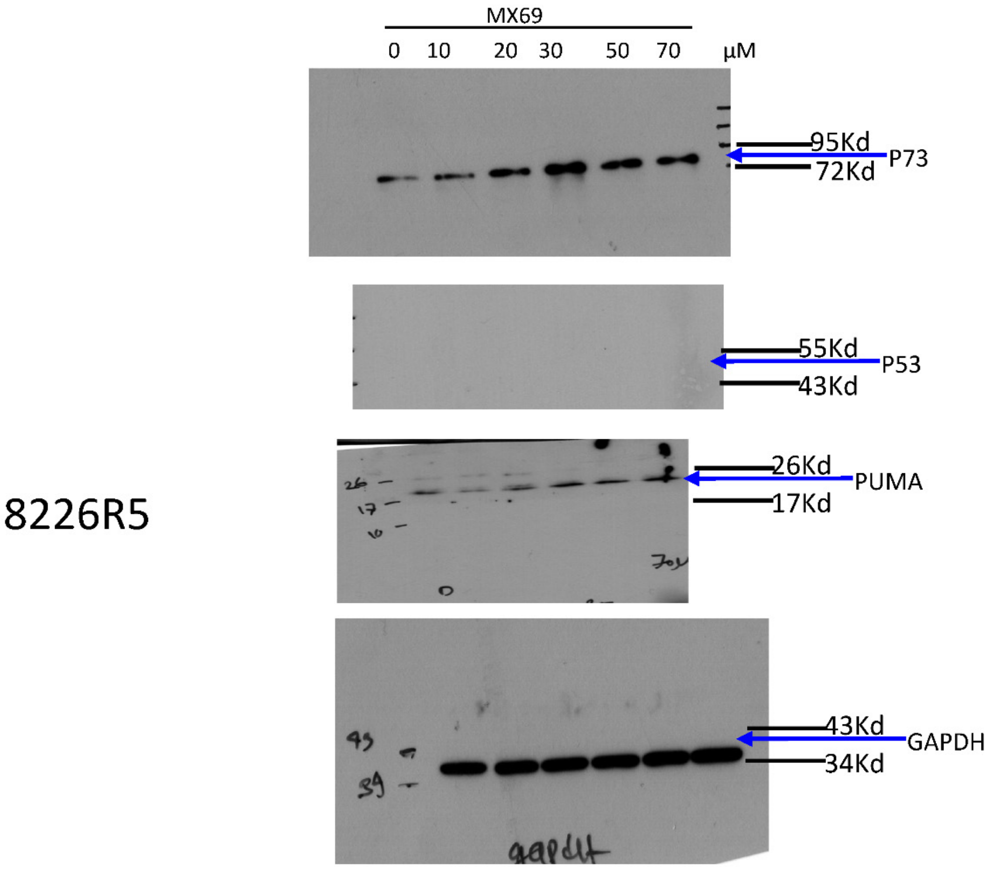

P73 and PUMA proteins Intensity ratio (GAPDH was used as normalization standard) were calculated respective treatment with 70 µM MX69.

MX69	DMSO	10µM	20 µM	30 µM	50 µM	70 µM
P73	0.35	0.44	0.63	1.19	1.05	1
PUMA	0.3	0.37	0.45	0.59	0.71	1

Figure S14. Uncropped blots for Figure 3D.

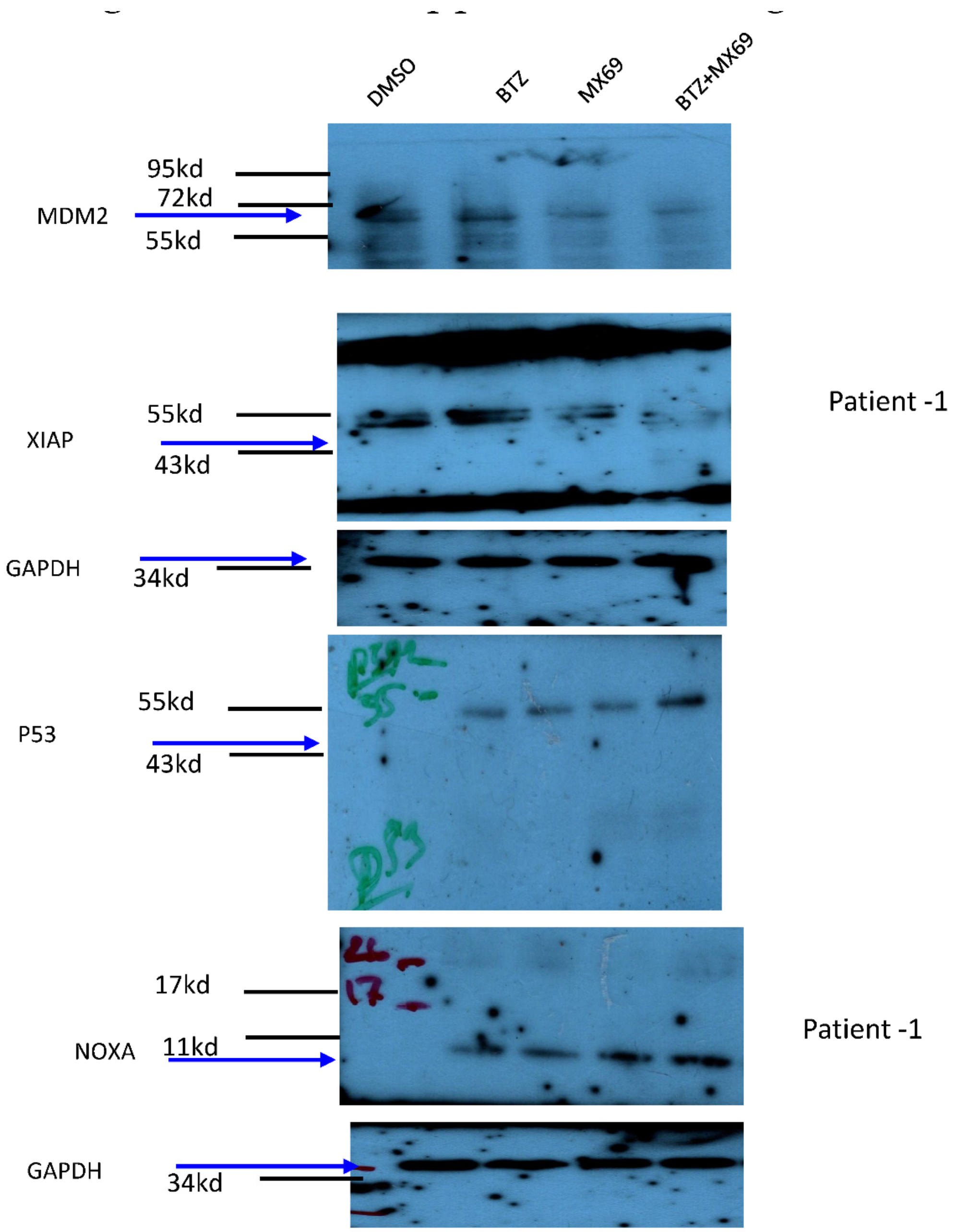

MDM2. XIAP, P53 and NOXA proteins Intensity ratio (GAPDH was used as normalization standard) were calculated respective treatment with DMSO and BTZ+MX69.

	DMSO	BTZ	MX69	BTZ+MX69
MDM 2	1	0.87	0.53	0.23
XIAP	1	1.21	0.56	0.21
P53	0.34	0.41	0.39	1
NOXA	0.32	0.43	0.85	1

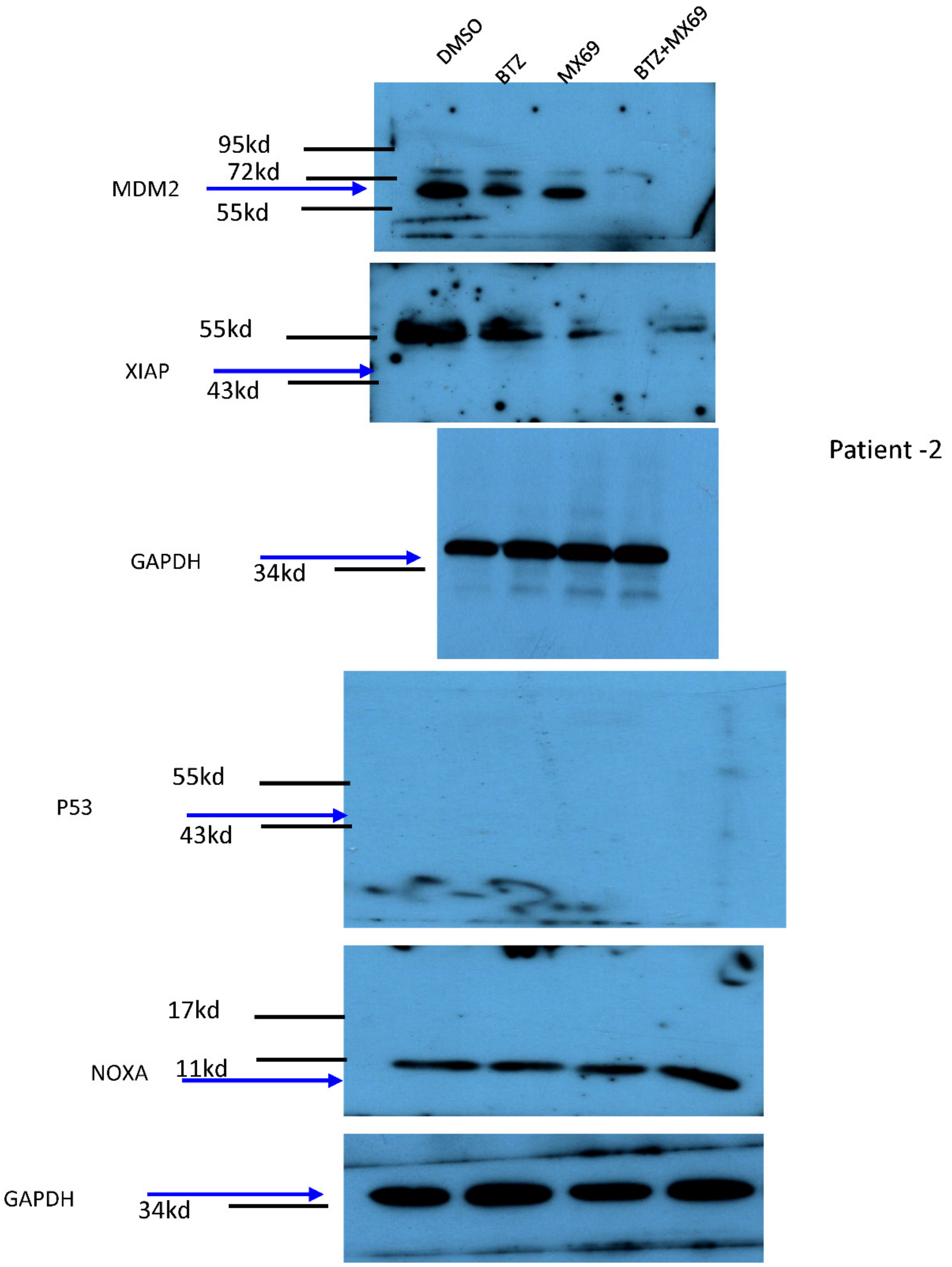

MDM2. XIAP and NOXA proteins Intensity ratio (GAPDH was used as normalization standard) were calculated respective treatment with DMSO and and BTZ+MX69.

	DMSO	BTZ	MX69	BTZ+MX69
MDM2	1	0.84	0.73	0.13
XIAP	1	0.78	0.43	0.22
NOXA	0.43	0.54	0.65	1

Figure S15. Uncropped blots for Figure 4A.

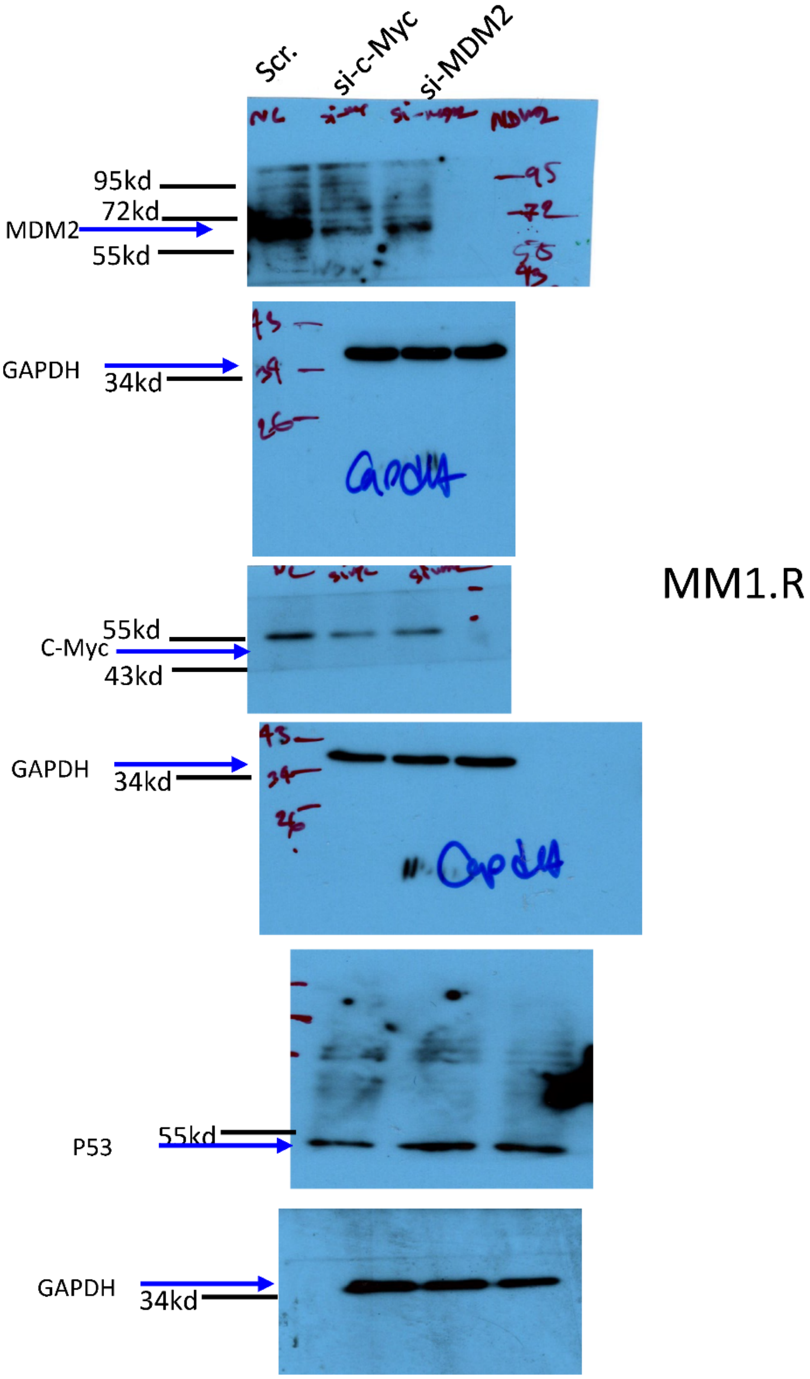

MDM2. C-Myc and P53 proteins Intensity ratio (GAPDH was used as normalization standard) were calculated respective transfection with Scramble and si-MDM2.

	Scr.	Si-c-Myc	Si-MDM2
MDM2	1	0.34	0.44
c-Myc	1	0.25	0.29
P53	0.44	1.05	1

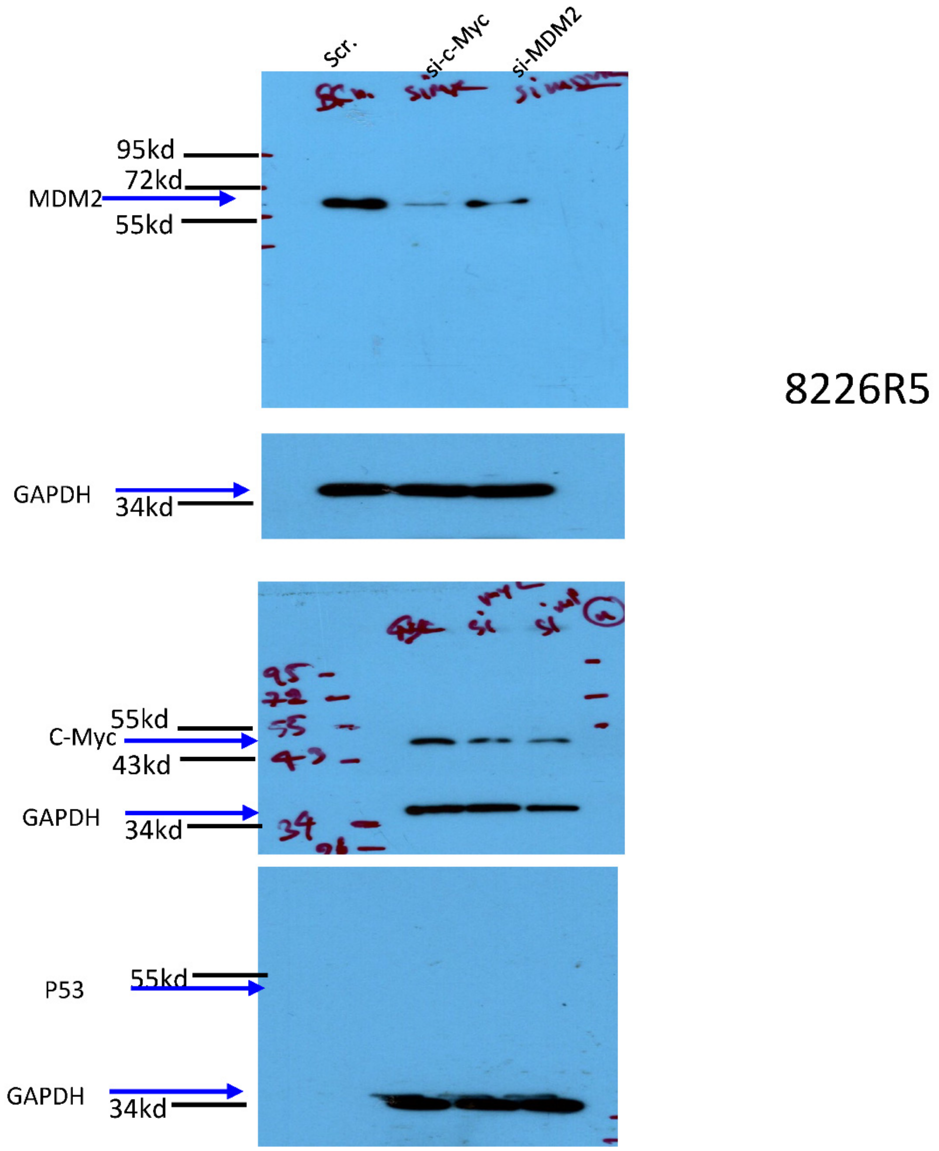

MDM2 and C-Myc proteins Intensity ratio (GAPDH was used as normalization standard) were calculated respective transfection with Scramble.

	Scr.	Si-c-Myc	Si-MDM2
MDM2	1	0.21	0.29
c-Myc	1	0.34	0.23

Figure S16. Uncropped blots for Figure 5A.

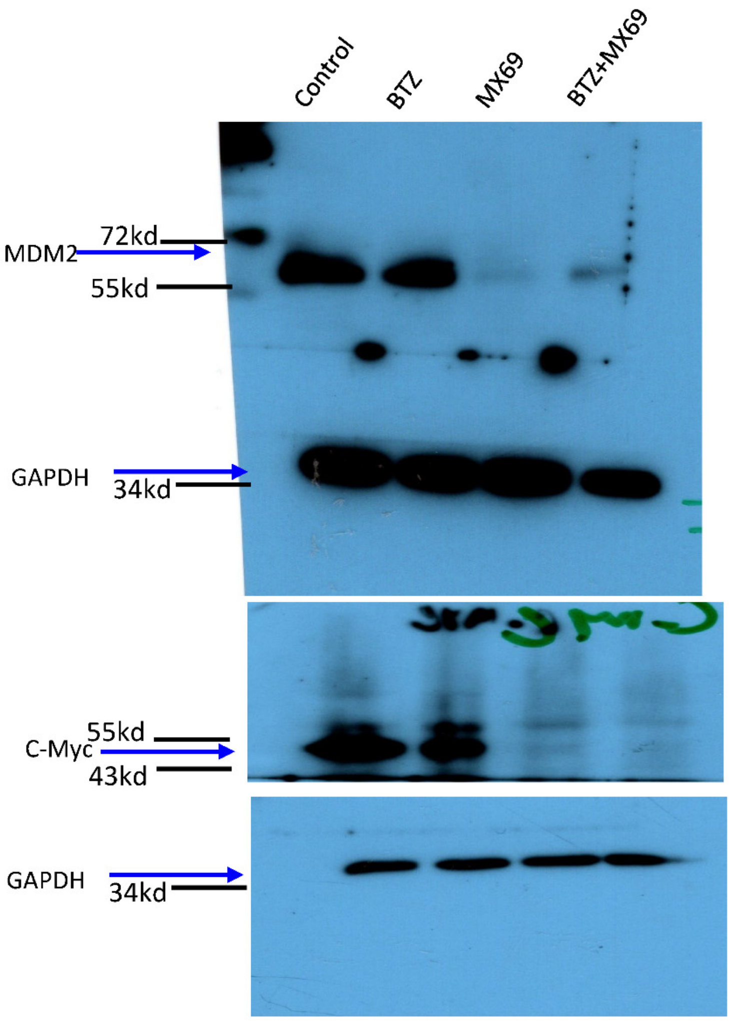

MDM2 and C-Myc proteins Intensity ratio (GAPDH was used as normalization standard) were calculated respective treatment with Control.

	Control	BTZ	MX69	BTZ+MX69
MDM2	1	0.93	0.23	0.34
c-Myc	1	0.91	0.36	0.26

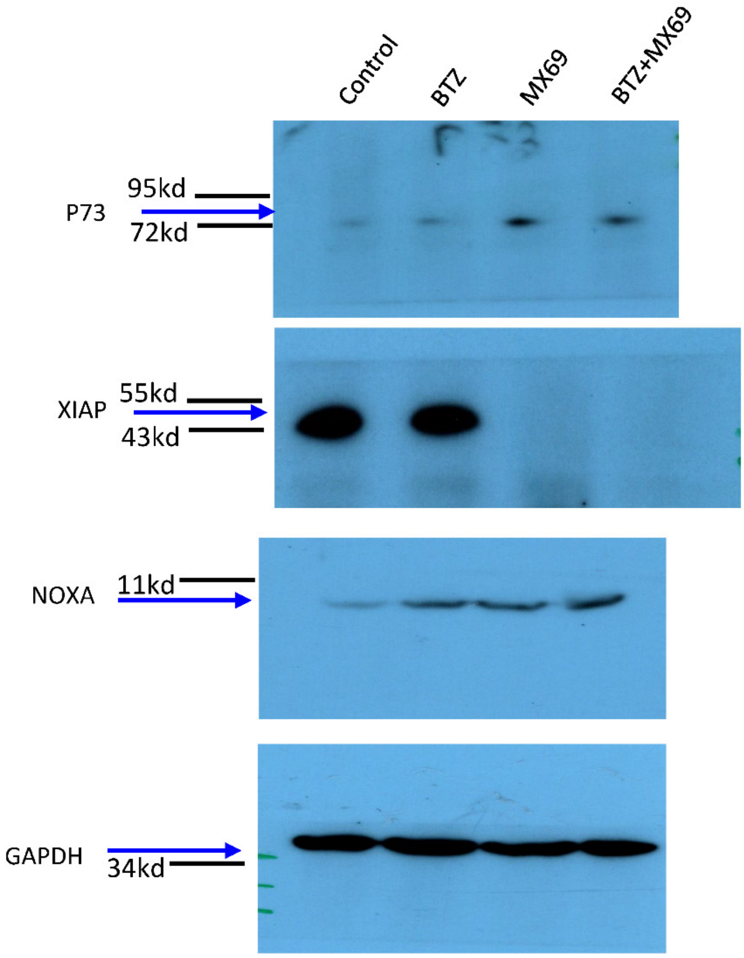

P73,NOXA and XIAPproteins Intensity ratio (GAPDH was used as normalization standard) were calculated respective treatment with BTZ+MX69 and Control.

	Control	BTZ	MX69	BTZ+MX69
P73	0.34	0.43	1.1	1
XIAP	1	1.04	0.11	0.1
NOXA	0.25	0.47	0.77	1

Figure S17. Uncropped blots for Figure 7G.

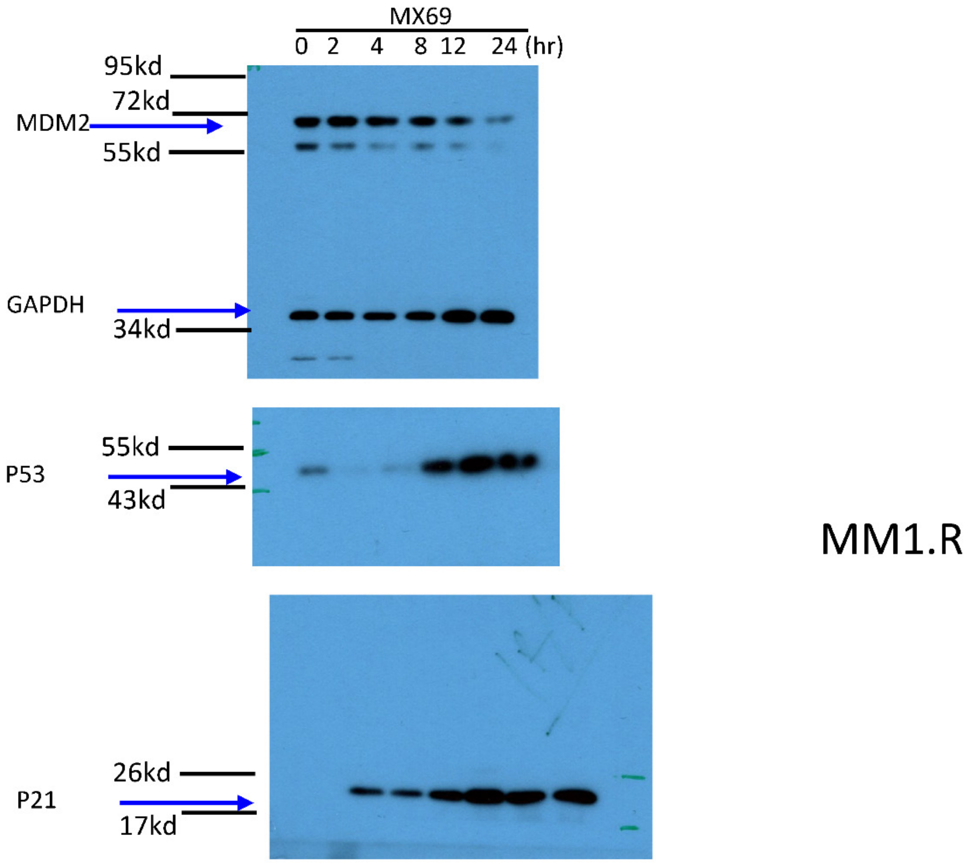

MDM2 and (P53 and P21) proteins Intensity ratio (GAPDH was used as normalization standard) were calculated respective treatment with zero hour and 24hour treatment .

	0hr	2hr	4hr	8hr	12hr	24hr
MDM2	1	1.05	0.93	0.72	0.51	0.24
P53	0.39	0.2	0.34	0.47	1.08	1
P21	0.35	0.38	0.45	1.34	0.92	1

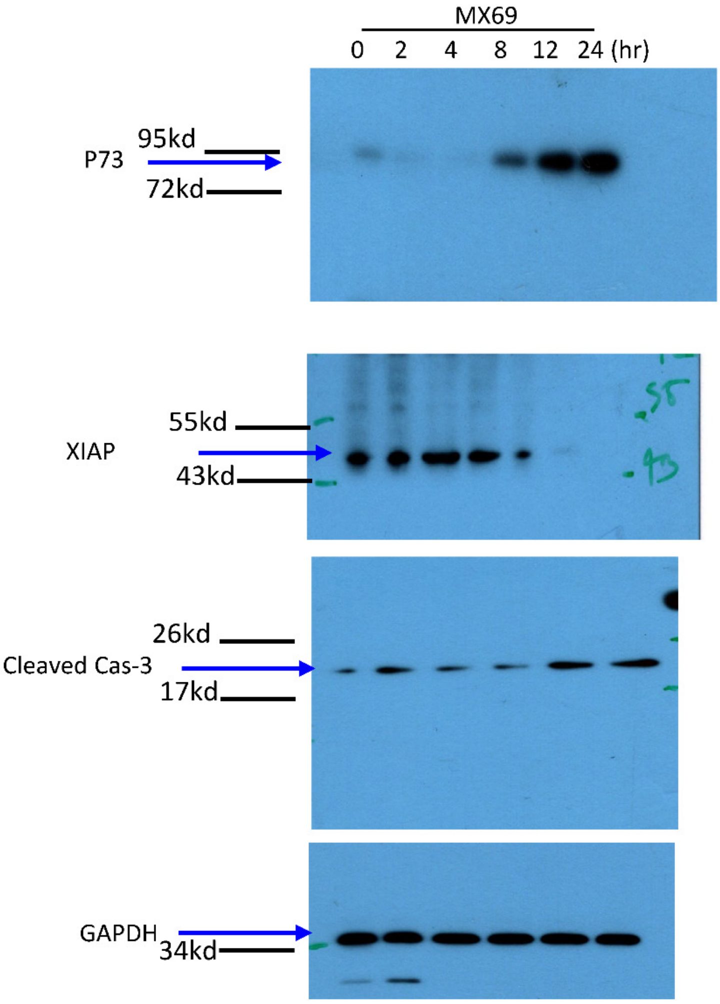

MM1.R

XIAP and (P73 and Cleaved Cas-3) proteins Intensity ratio (GAPDH was used as normalization standard) were calculated respective treatment with zero hour and 24hour treatment .

	0hr	2hr	4hr	8hr	12hr	24hr
P73	0.39	0.2	0.26	0.48	0.94	1
XIAP	1	0.93	1.1	0.84	0.41	0.21
Cleaved Cas-3	0.27	0.41	0.36	0.32	1.07	1

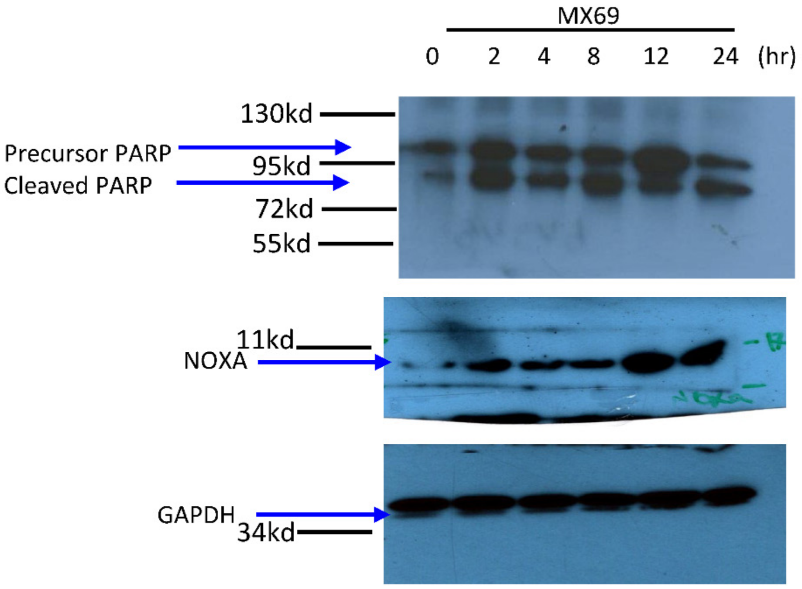

Cleaved PARP and NOXA proteins Intensity ratio (GAPDH was used as normalization standard) were calculated respective treatment with 24hour treatment.

	0hr	2hr	4hr	8hr	12hr	24hr
Cleaved PARP	0.34	0.68	0.88	1.03	0.79	1
NOXA	0.23	0.37	0.41	0.46	1.09	1

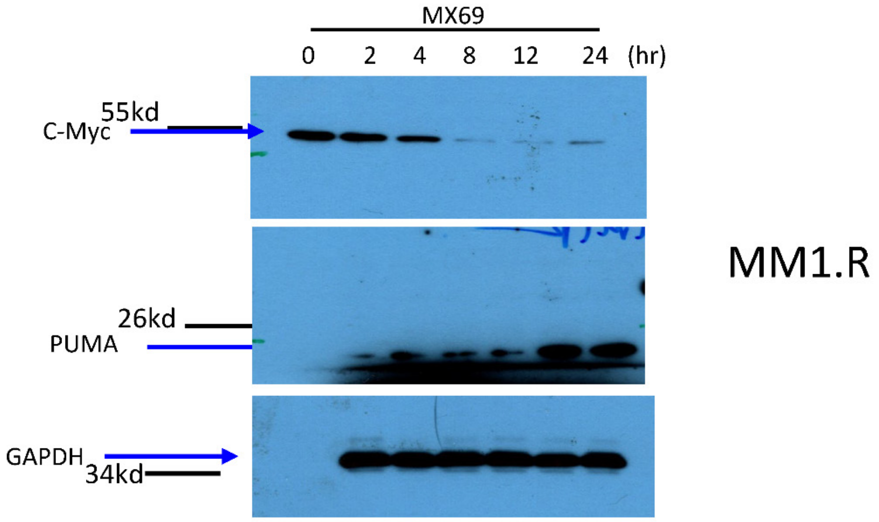

c-Myc and PUMA proteins Intensity ratio (GAPDH was used as normalization standard) were calculated respective treatment with zero hour and 24hour treatment.

	0hr	2hr	4hr	8hr	12hr	24hr
c-Myc	1	1.01	0.86	0.33	0.21	0.36
PUMA	0.28	0.39	0.42	0.49	0.93	1

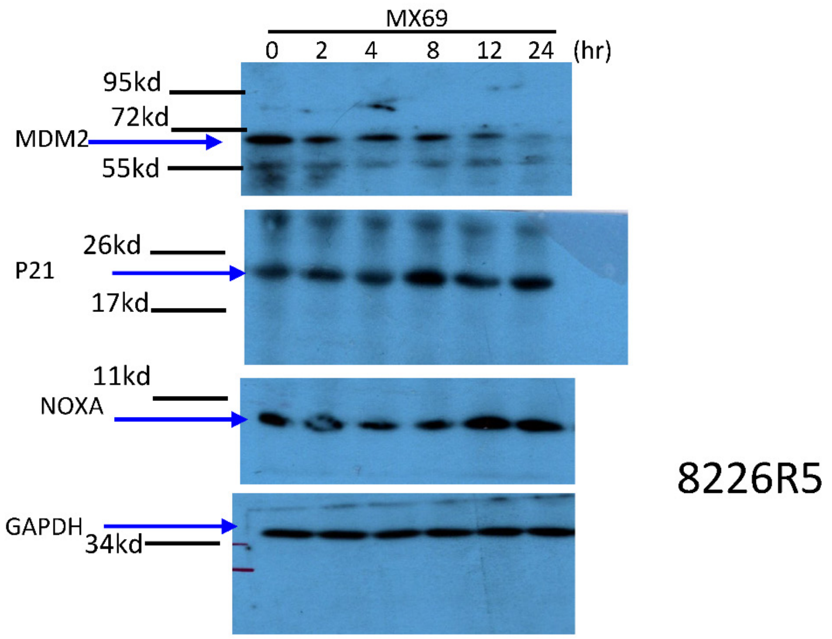

MDM2, P21 and NOXA proteins Intensity ratio (GAPDH was used as normalization standard) were calculated respective treatment with zero hour and 24hour.

	0hr	2hr	4hr	8hr	12hr	24hr
MDM2	1	0.78	0.64	0.69	0.38	0.22
P21	0.38	0.49	0.48	1.09	0.75	1
NOXA	0.49	0.47	0.61	0.69	1.09	1

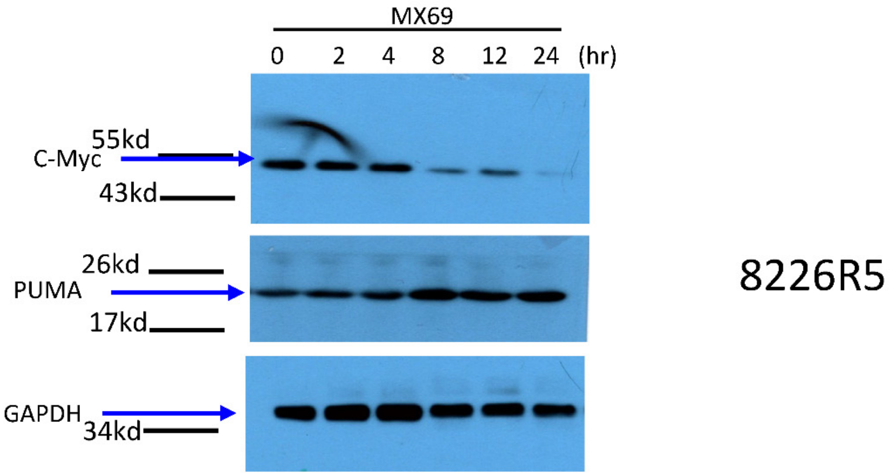

c-Myc and PUMA proteins Intensity ratio (GAPDH was used as normalization standard) were calculated respective treatment with zero hour and 24hour.

	0hr	2hr	4hr	8hr	12hr	24hr
c-Myc	1	0.83	0.87	0.43	0.48	0.32
PUMA	0.4	0.51	0.54	1.09	1.06	1

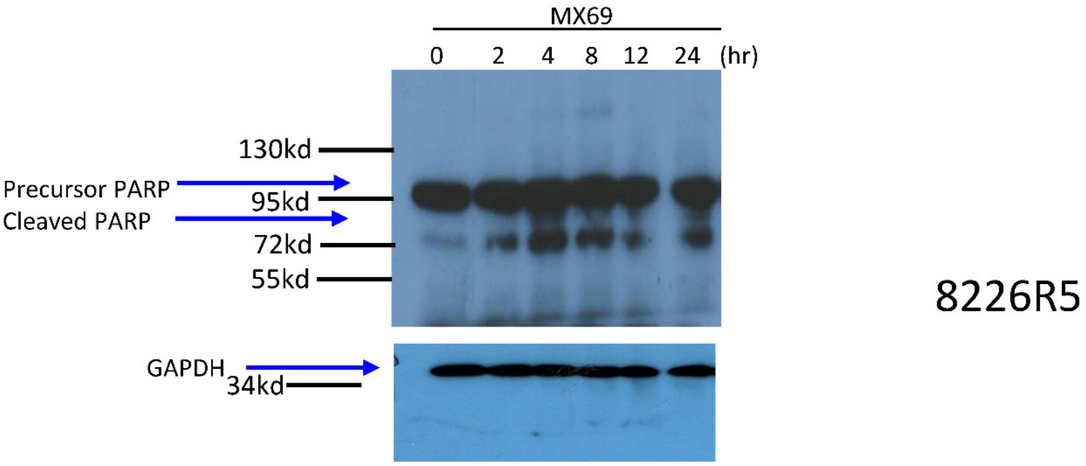

Cleaved PARP proteins Intensity ratio (GAPDH was used as normalization standard) were calculated respective treatment with zero hour treatment.

	0hr	2hr	4hr	8hr	12hr	24hr
Cleaved PARP	0.43	0.79	1.17	1.09	0.63	1

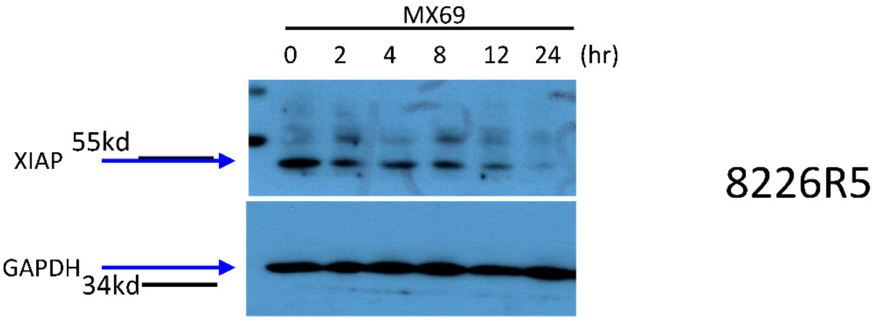

XIAP protein Intensity ratio (GAPDH was used as normalization standard) was calculated respective treatment with zero hour treatment.

	0hr	2hr	4hr	8hr	12hr	24hr
XIAP	1	0.77	0.63	0.58	0.5	0.27

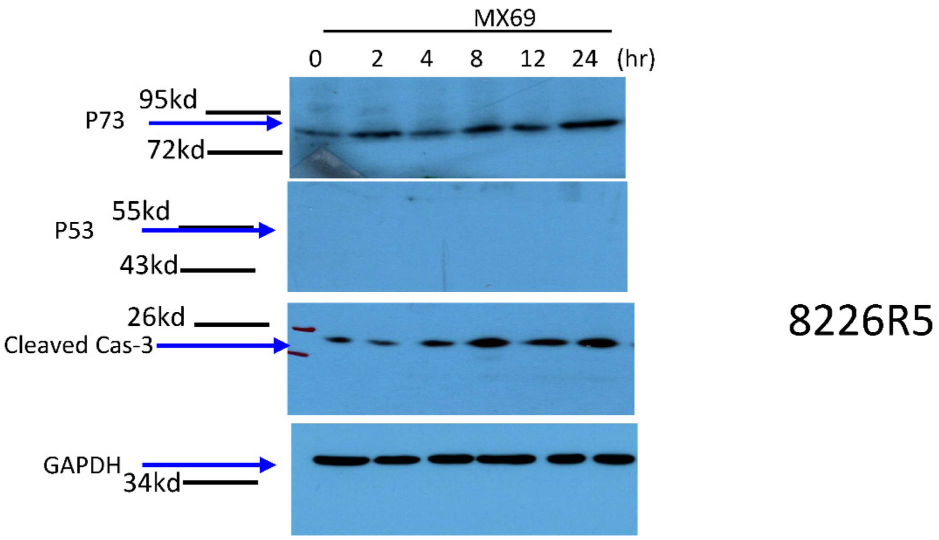

P73 and Cleaved Cas-3 proteins Intensity ratio (GAPDH was used as normalization standard) were calculated respective treatment with 24hour treatment.

	0hr	2hr	4hr	8hr	12hr	24hr
P73	0.32	0.5	0.44	0.79	0.56	1
Cleaved Cas-3	0.24	0.25	0.58	1.07	0.77	1

Figure S18. Uncropped blots for Figure S4A.

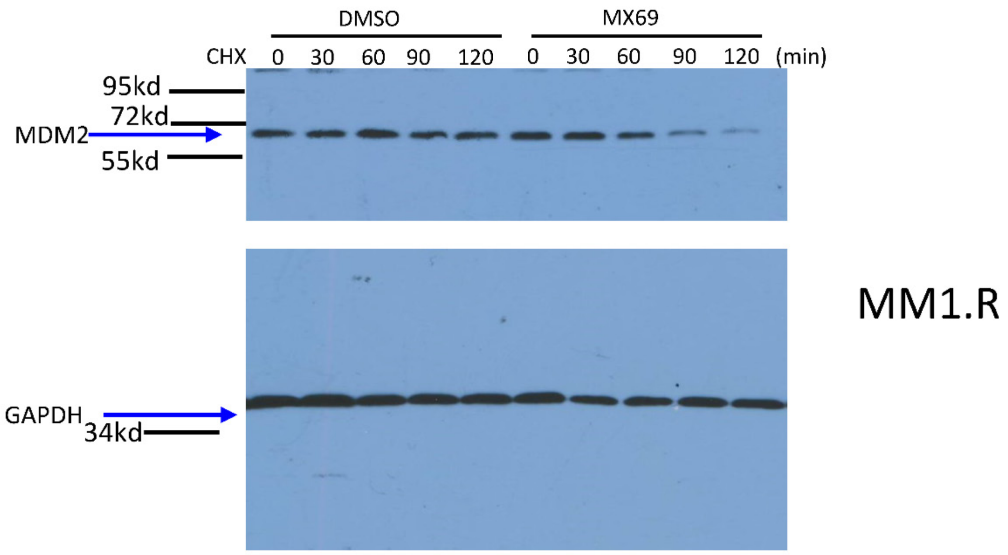

MDM2 protein Intensity ratio (GAPDH was used as normalization standard) was calculated respective treatment with zero minute treatment.

CHX										
	DMSO					MX69				
Min.	0	30	60	90	120	0	30	60	90	120
MDM 2	1	0.93	0.89	0.6	0.51	1	0.73	0.52	0.32	0.25

Figure S19. Uncropped blots for Figure S4B.

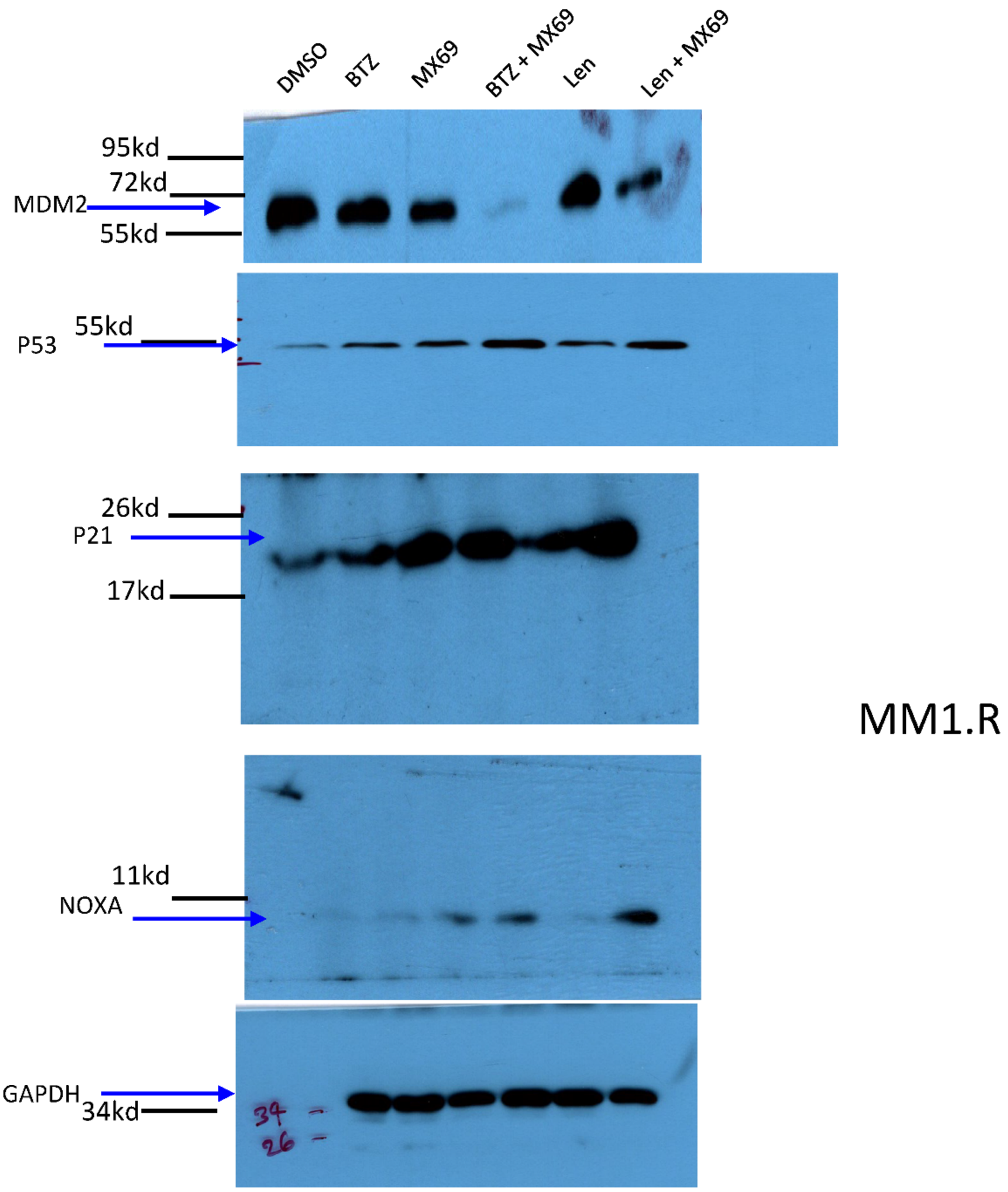

MDM2, P53, P21 and NOXA proteins Intensity ratio (GAPDH was used as normalization standard) were calculated respective treatment with DMSO and Len+MX69 treatment.

	DMSO	BTZ	MX69	BTZ+MX69	LEN	Len+MX69
MDM2	1	0.96	0.82	0.23	0.84	0.35
P53	0.3	0.46	0.56	1.14	0.53	1
P21	0.4	0.49	1.03	0.92	0.58	1
NOXA	0.37	0.43	0.78	0.93	0.44	1

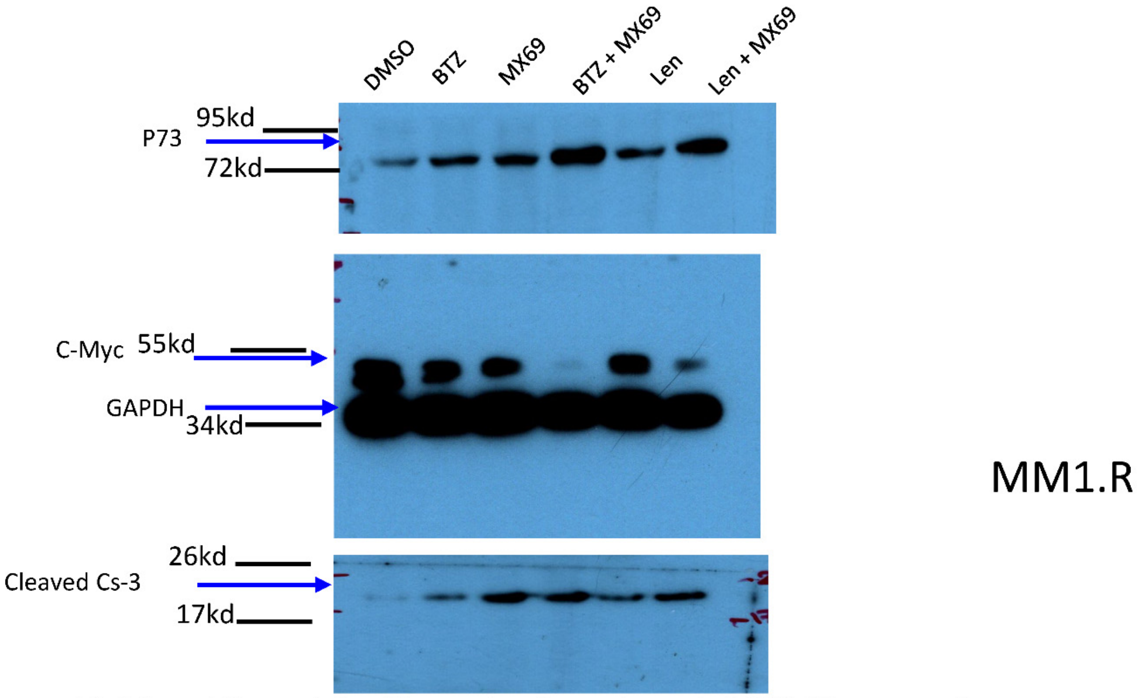

c-Myc and (P73 and Cleaved Cas-3) proteins Intensity ratio (GAPDH was used as normalization standard) were calculated respective treatment with DMSO Len+MX69 treatment.

	DMSO	BTZ	MX69	BTZ+MX69	LEN	Len+MX69
P73	0.34	0.56	0.71	1.1	0.44	1
c-Myc	1	0.88	0.72	0.32	0.87	0.48
Cleaved Cas-3	0.27	0.54	1.09	1.1	0.66	1

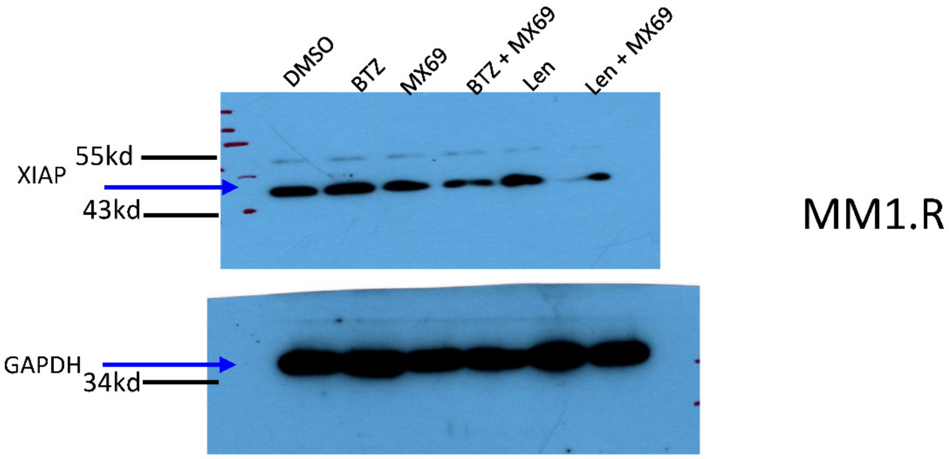

XIAP protein Intensity ratio (GAPDH was used as normalization standard) was calculated respective treatment with DMSO treatment.

	DMSO	BTZ	MX69	BTZ+MX69	LEN	Len+MX69
XIAP	1	1.01	0.95	0.46	0.75	0.21

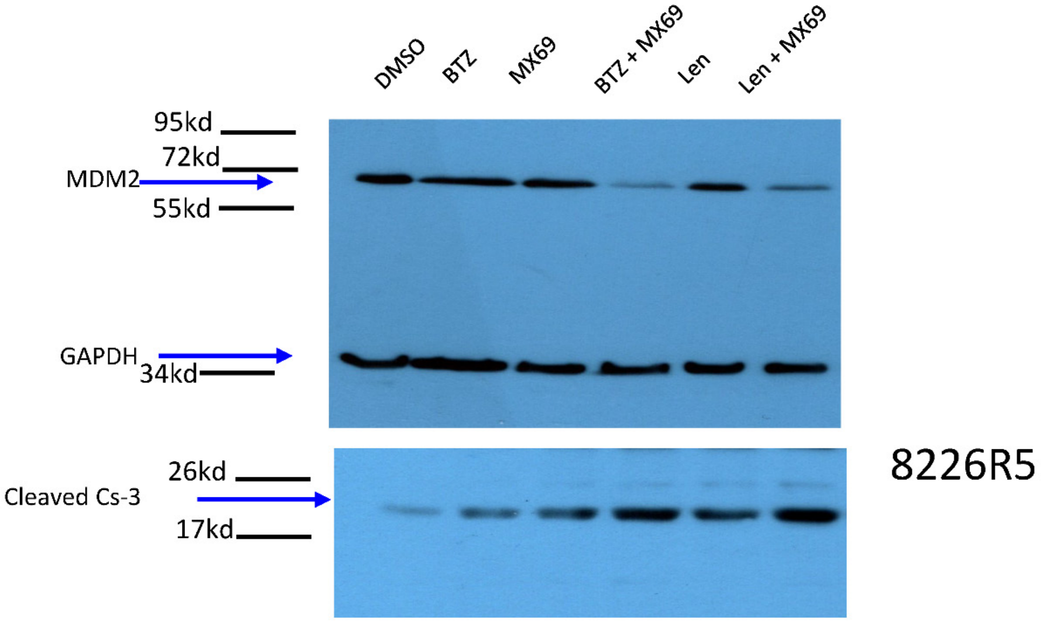

MDM2 and Cleaved Cas-3 proteins Intensity ratio (GAPDH was used as normalization standard) were calculated respective treatment with DMSO and Len+MX69 treatment.

	DMSO	BTZ	MX69	BTZ+MX69	LEN	Len+MX69
MDM2	1	1.02	0.86	0.33	0.77	0.27
Cleaved Cas-3	0.32	0.4	0.53	0.96	0.55	1

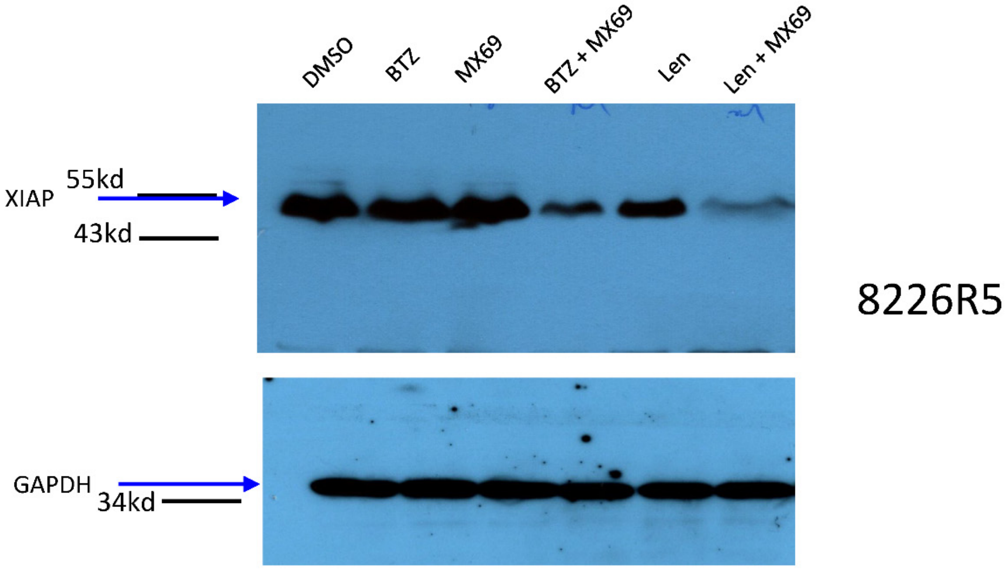

XIAP protein Intensity ratio (GAPDH was used as normalization standard) was calculated respective treatment with DMSO treatment.

	DMSO	BTZ	MX69	BTZ+MX69	LEN	Len+MX69
XIAP	1	1.01	1.06	0.41	0.79	0.33

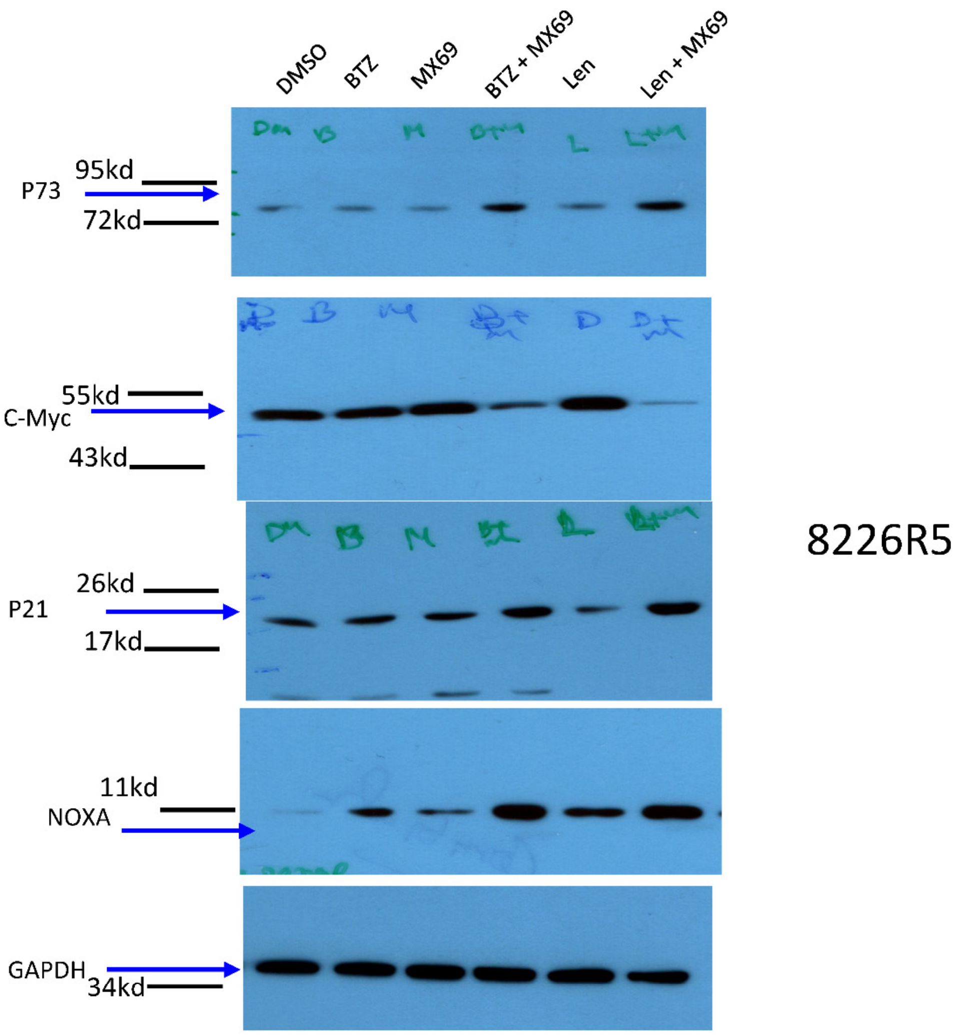

P53, c-Myc, P21 and NOXA proteins Intensity ratio (GAPDH was used as normalization standard) were calculated respective treatment with Len+MX69 treatment.

	DMSO	BTZ	MX69	BTZ+MX69	LEN	Len+MX69
P73	0.27	0.36	0.44	0.94	0.4	1
c-Myc	1	1.02	2.03	0.41	0.92	0.25
P21	0.43	0.55	0.58	0.91	0.39	1
NOXA	0.27	0.49	0.34	0.97	0.56	1

Figure S20. Uncropped blots for Figure S5E.

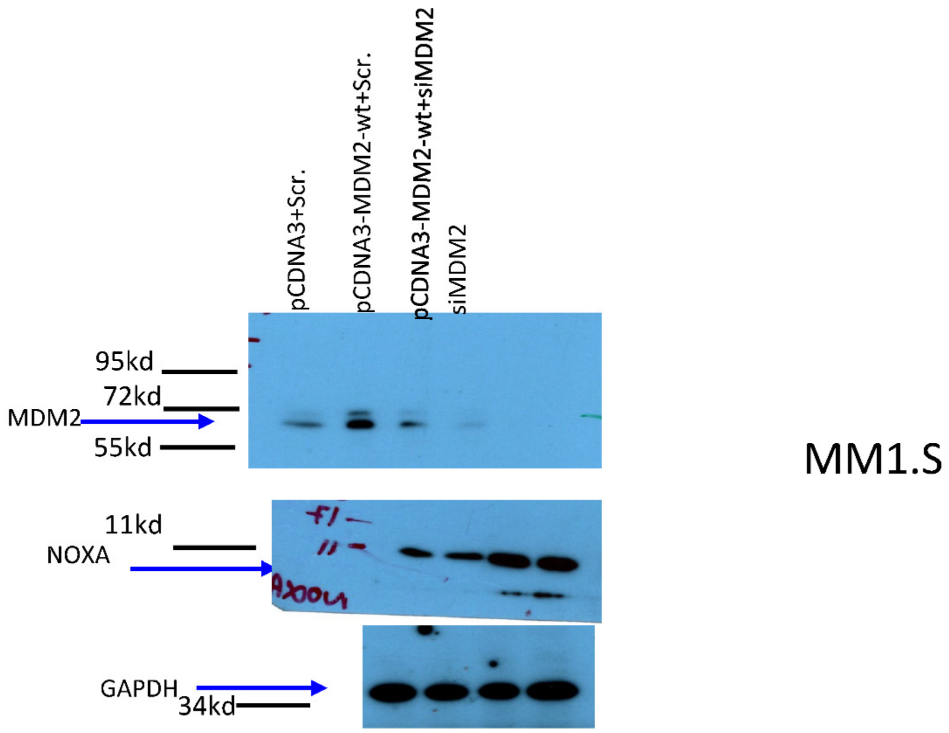

MDM2 and NOXA proteins Intensity ratio (GAPDH was used as normalization standard) were calculated respective transfect with pCDNA3+Scr and si-MDM2.

	pCDNA3+Scr.	pCDNA3-MDM2-wt+Scr.	pCDNA3-MDM2-wt+siMDM2	siMDM2
MDM2	1	4.5	1.05	0.54
NOXA	0.43	0.48	1.3	1

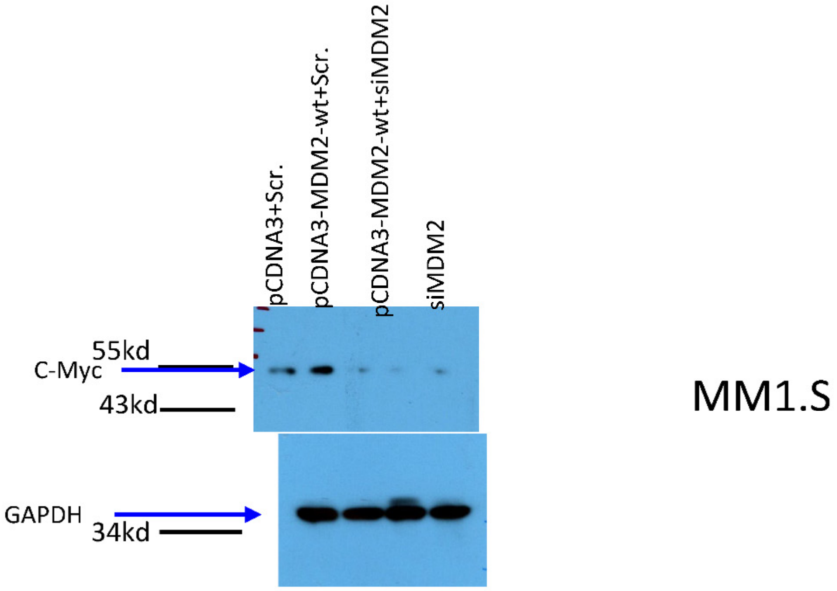

c-Myc proteins Intensity ratio (GAPDH was used as normalization standard) were calculated respective transfect with pCDNA3+Scr.

	pCDNA3+Scr.	pCDNA3-MDM2-wt+Scr.	pCDNA3-MDM2-wt+siMDM2	siMDM2
c-Myc	1	2.3	0.45	0.38

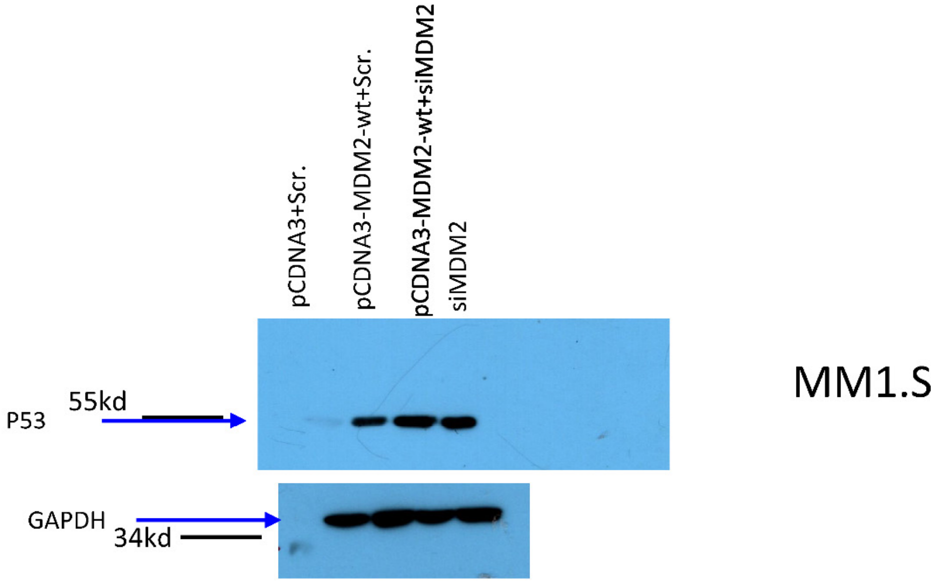

P53 proteins Intensity ratio (GAPDH was used as normalization standard) were calculated respective transfect with si-MDM2

	pCDNA3+Scr.	pCDNA3-MDM2-wt+Scr.	pCDNA3-MDM2-wt+siMDM2	siMDM2
P53	0.36	0.83	1.09	1

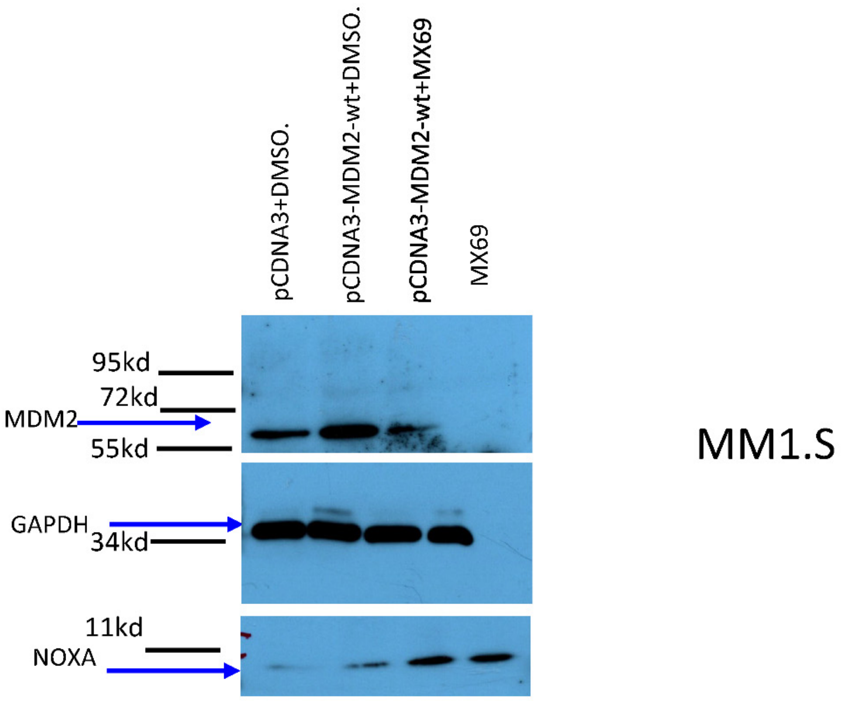

MDM2 and NOXA proteins Intensity ratio (GAPDH was used as normalization standard) were calculated respective transfect with pCDNA3+DMSO and MX69

	pCDNA3+DMSO	pCDNA3-MDM2-wt+DMSO	pCDNA3-MDM2-wt+MX69	MX69
MDM2	1	2.5	0.83	0.22
NOXA	0.22	0.34	1.01	1

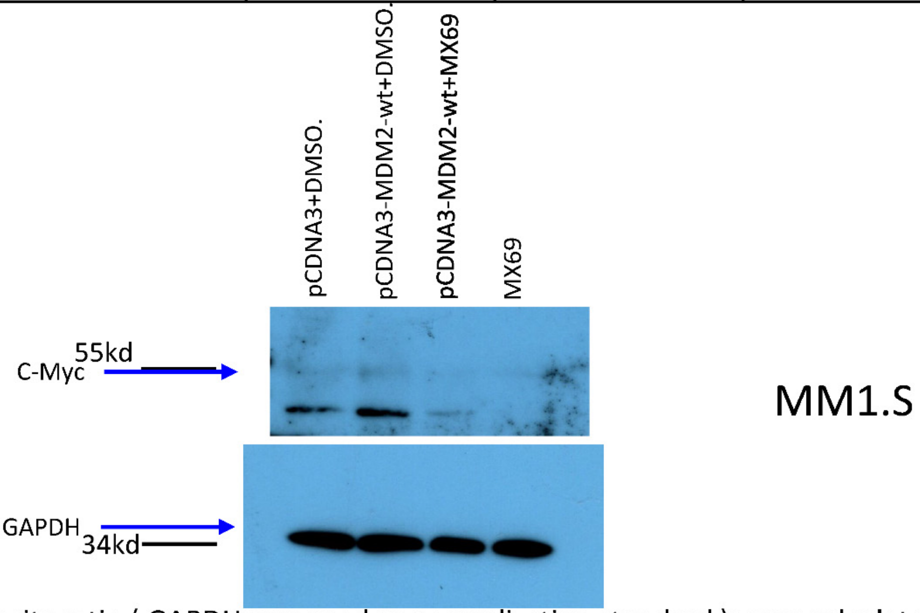

c-Myc proteins Intensity ratio (GAPDH was used as normalization standard) were calculated respective transfect with pCDNA3+DMSO

	pCDNA3+DMSO	pCDNA3-MDM2-wt+DMSO	pCDNA3-MDM2-wt+MX69	MX69
c-Myc	1	2.7	0.55	0.2

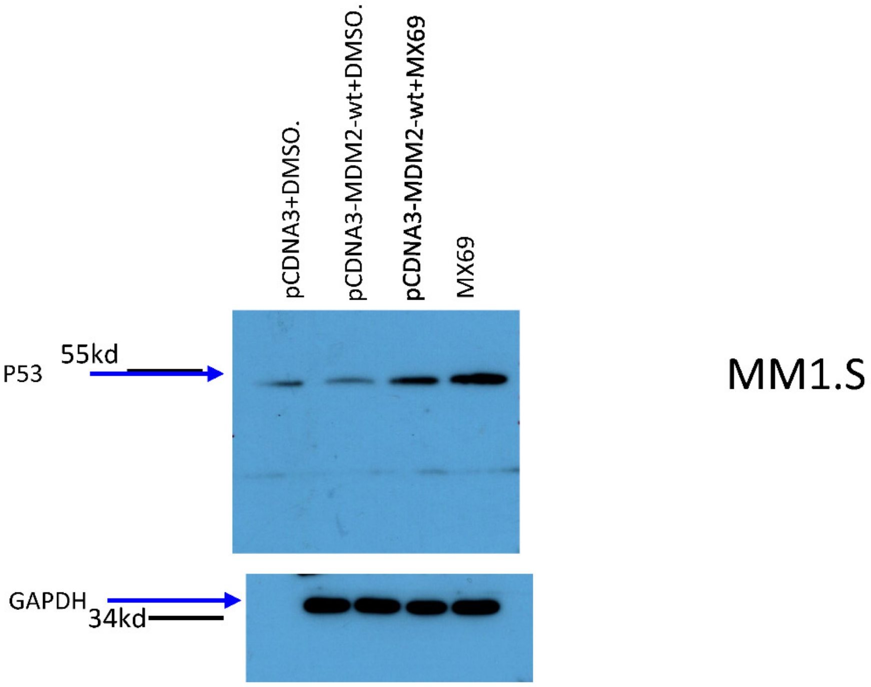

P53 proteins Intensity ratio (GAPDH was used as normalization standard) were calculated respective MX69

	pCDNA3+DMSO	pCDNA3-MDM2-wt+DMSO	pCDNA3-MDM2-wt+MX69	MX69
P53	0.33	0.41	0.83	1

Figure S21. Uncropped blots for Figure S7A.

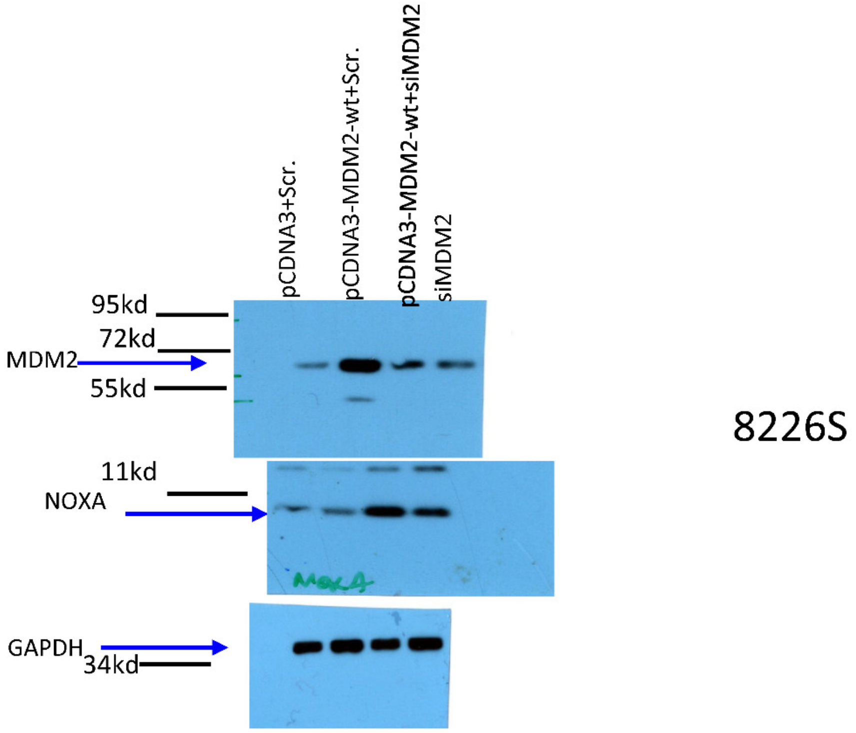

MDM2 and NOXA proteins Intensity ratio (GAPDH was used as normalization standard) were calculated respective transfect with pCDNA3+Scr and si-MDM2.

	pCDNA3+Scr.	pCDNA3-MDM2-wt+Scr.	pCDNA3-MDM2-wt+siMDM2	siMDM2
MDM2	1	5.5	2.01	1.1
NOXA	0.22	0.34	1.4	1

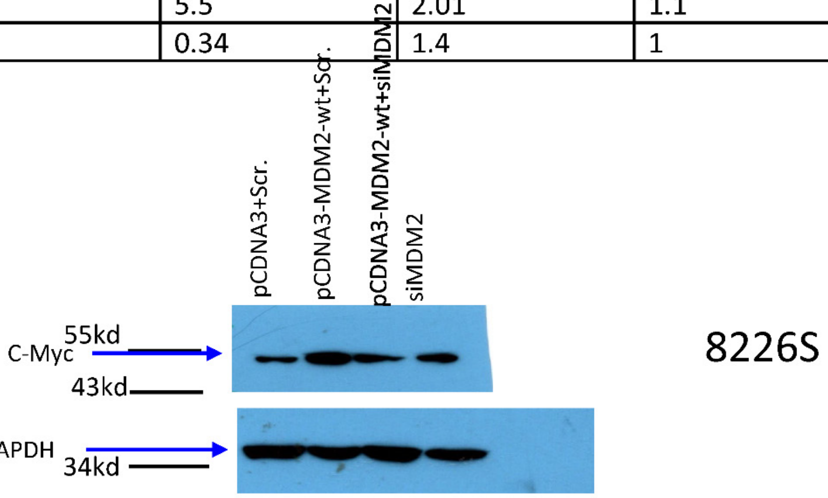

c-Myc proteins Intensity ratio (GAPDH was used as normalization standard) were calculated respective transfect with pCDNA3+Scr.

	pCDNA3+Scr.	pCDNA3-MDM2-wt+Scr.	pCDNA3-MDM2-wt+siMDM2	siMDM2
c-Myc	1	2.8	0.94	0.53

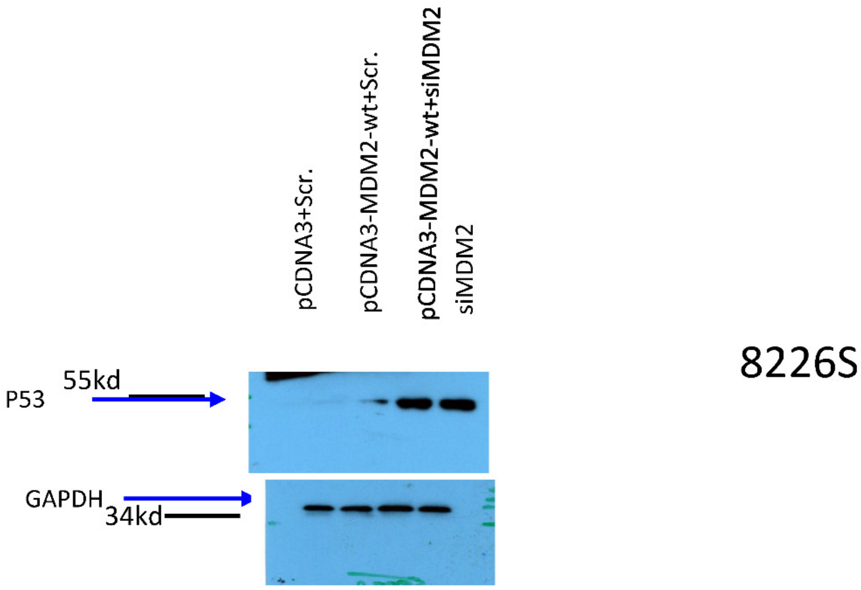

P53 proteins Intensity ratio (GAPDH was used as normalization standard) were calculated respective transfect with si-MDM2

	pCDNA3+Scr.	pCDNA3-MDM2-wt+Scr.	pCDNA3-MDM2-wt+siMDM2	siMDM2
P53	0.2	0.4	0.98	1

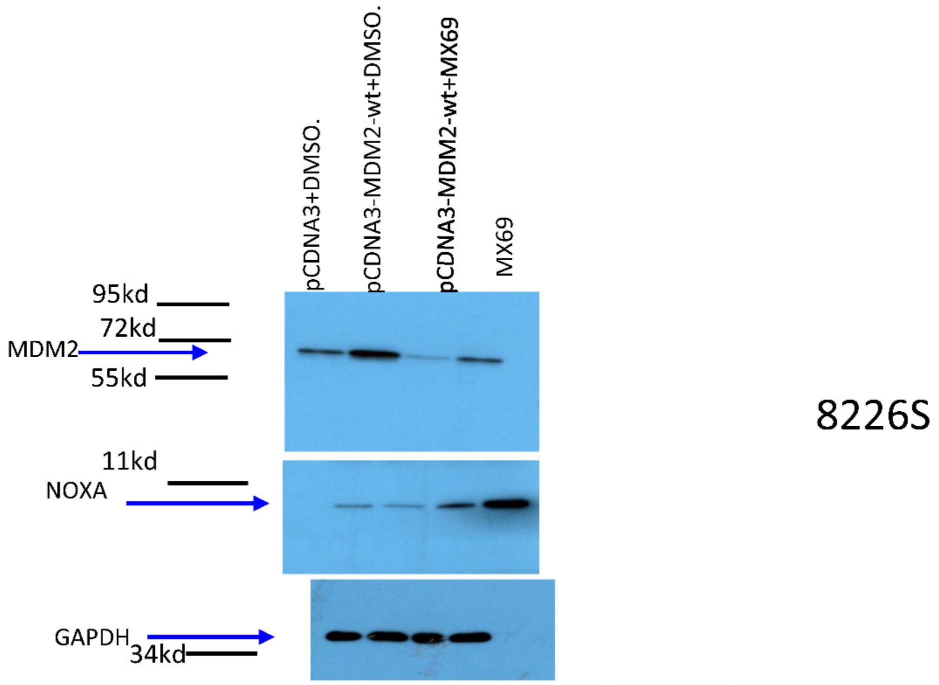

MDM2 and NOXA proteins Intensity ratio (GAPDH was used as normalization standard) were calculated respective transfect with pCDNA3+DMSO and MX69

	pCDNA3+DMSO	pCDNA3-MDM2-wt+DMSO	pCDNA3-MDM2-wt+MX69	MX69
MDM2	1	3.2	0.32	0.41
NOXA	0.22	0.1	0.65	1

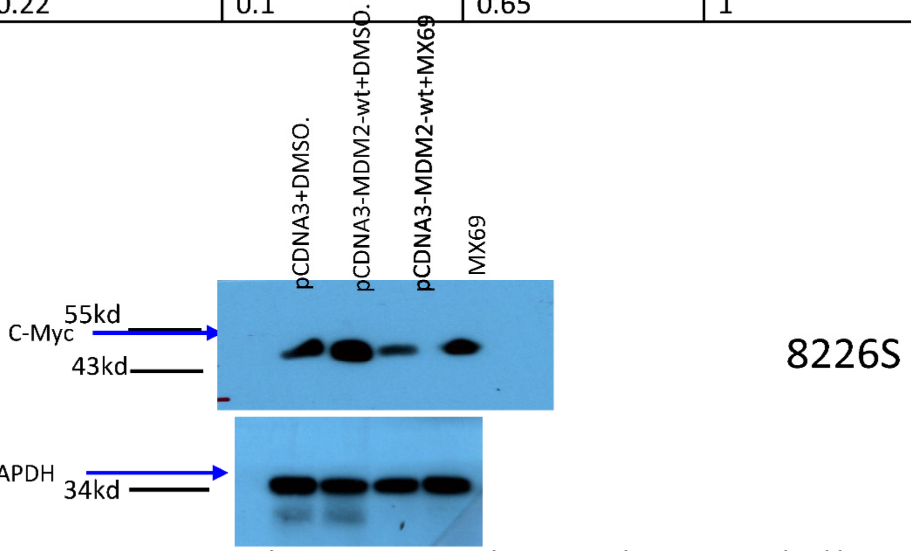

c-Myc proteins Intensity ratio (GAPDH was used as normalization standard) were calculated respective transfect with pCDNA3+DMSO

	pCDNA3+DMSO	pCDNA3-MDM2-wt+DMSO	pCDNA3-MDM2-wt+MX69	MX69
c-Myc	1	3.01	0.4	0.47

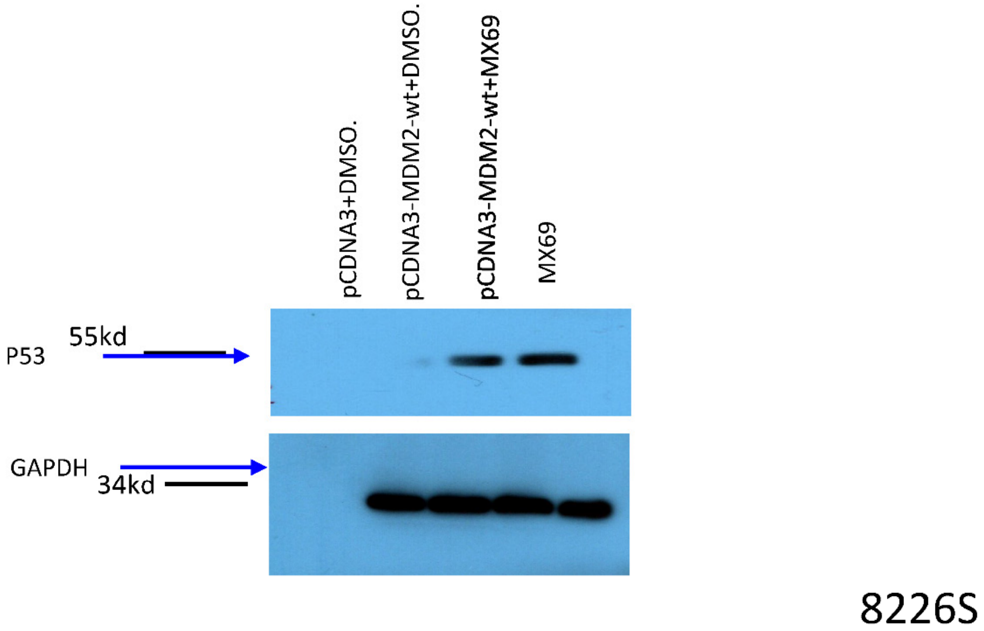

P53 proteins Intensity ratio (GAPDH was used as normalization standard) were calculated respective transfect with MX69

	pCDNA3+DMSO	pCDNA3-MDM2-wt+DMSO	pCDNA3-MDM2-wt+MX69	MX69
P53	0.2	0.45	0.88	1

Figure S22. Uncropped blots for Figure S7B.

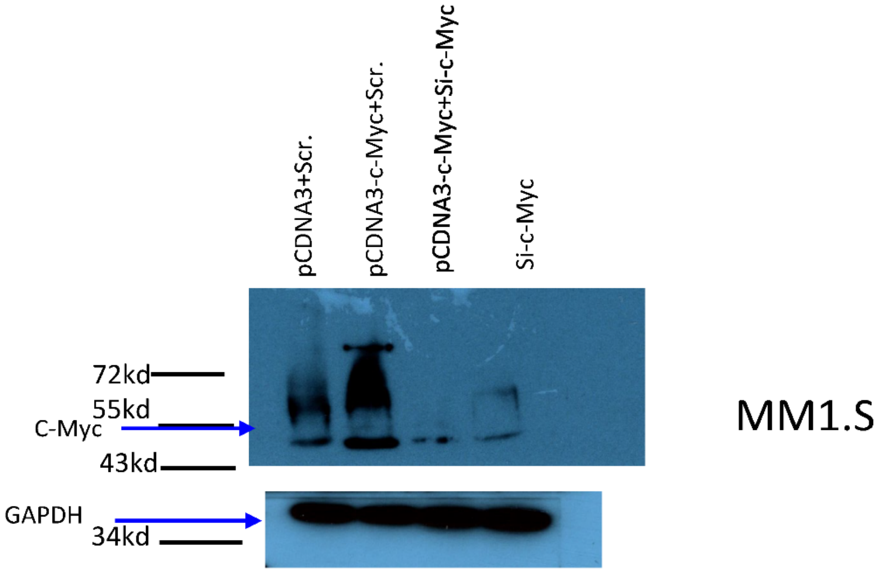

c-Myc proteins Intensity ratio (GAPDH was used as normalization standard) were calculated respective transfect with pCDNA3+Scr.

	pCDNA3+Scr.	pCDNA3-c-Myc+Scr.	pCDNA3-c-Myc+Si-c-Myc	Si-c-Myc
c-Myc	1	3.01	0.57	0.43

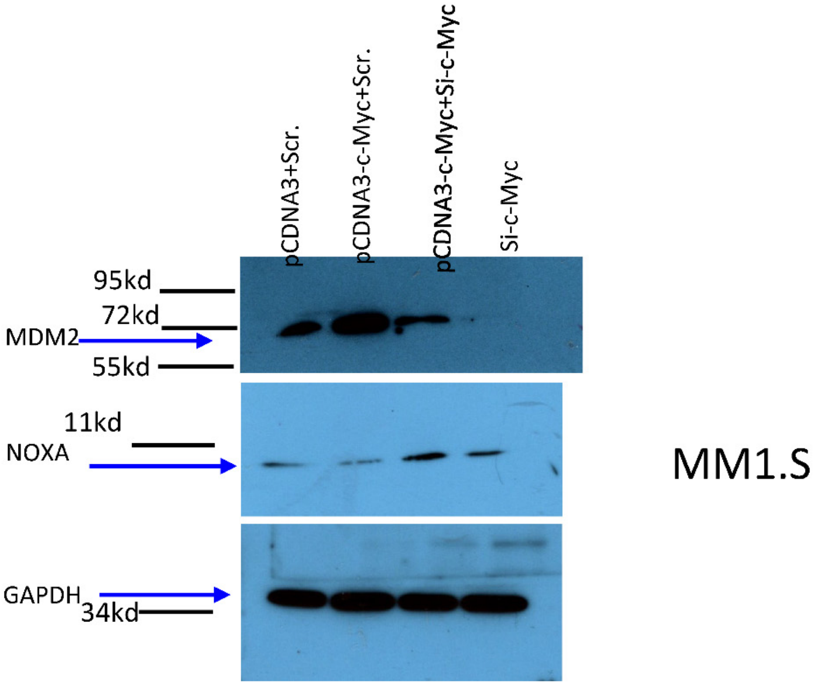

MDM2 and NOXA proteins Intensity ratio (GAPDH was used as normalization standard) were calculated respective transfect with pCDNA3+Scr and si-c-Myc

	pCDNA3+Scr.	pCDNA3-c-Myc+Scr.	pCDNA3-c-Myc+Si-c-Myc	Si-c-Myc
MDM2	1	4.1	1.2	0.21
NOXA	0.22	0.34	1.2	1

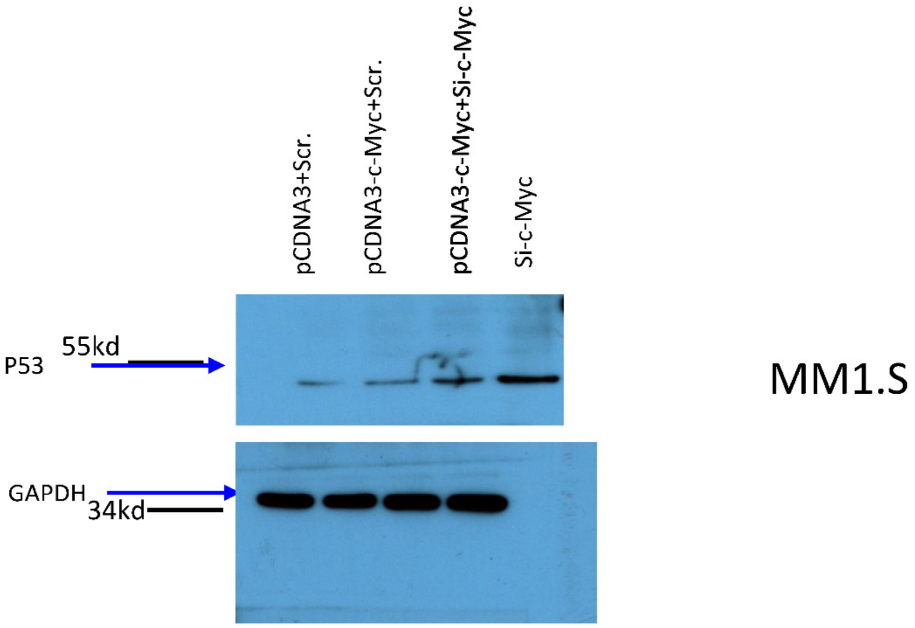

P53 proteins Intensity ratio (GAPDH was used as normalization standard) were calculated respective transfect with si-c-Myc.

	pCDNA3+Scr.	pCDNA3-c-Myc+Scr.	pCDNA3-c-Myc+ Si-c-Myc	Si-c-Myc
P53	0.25	0.28	0.78	1

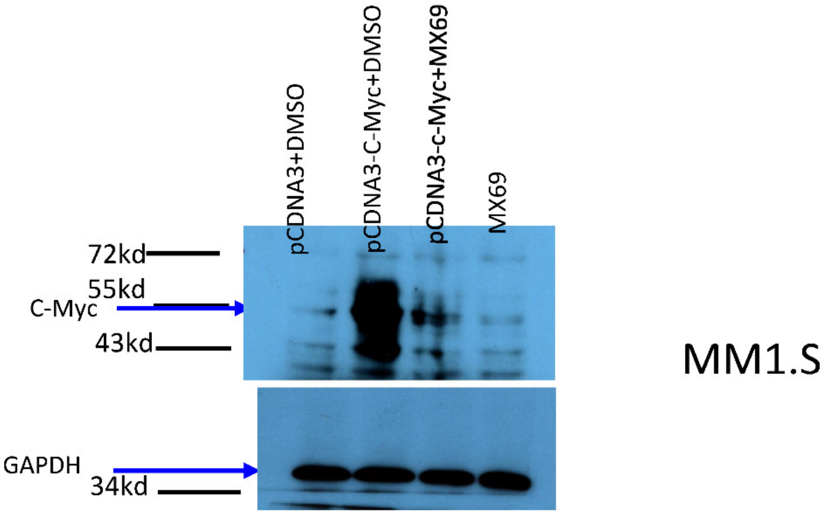

c-Myc proteins Intensity ratio (GAPDH was used as normalization standard) were calculated respective transfect with pCDNA3+DMSO

	pCDNA3+DMSO	pCDNA3-c-Myc+DMSO	pCDNA3-c-Myc+MX69	MX69
c-Myc	1	8.78	0.57	0.43

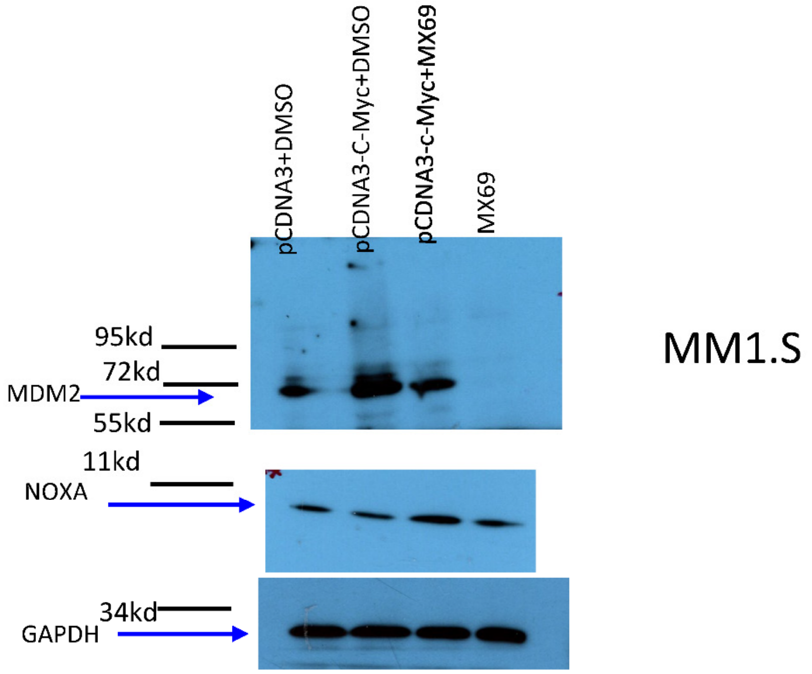

MDM2 and NOXA proteins Intensity ratio (GAPDH was used as normalization standard) were calculated respective transfect with pCDNA3+DMSO and MX69

	pCDNA3+DMSO	pCDNA3-c-Myc+DMSO	pCDNA3-c-Myc+MX69	MX69
MDM2	1	4.1	1.2	0.21
NOXA	0.22	0.34	1.2	1

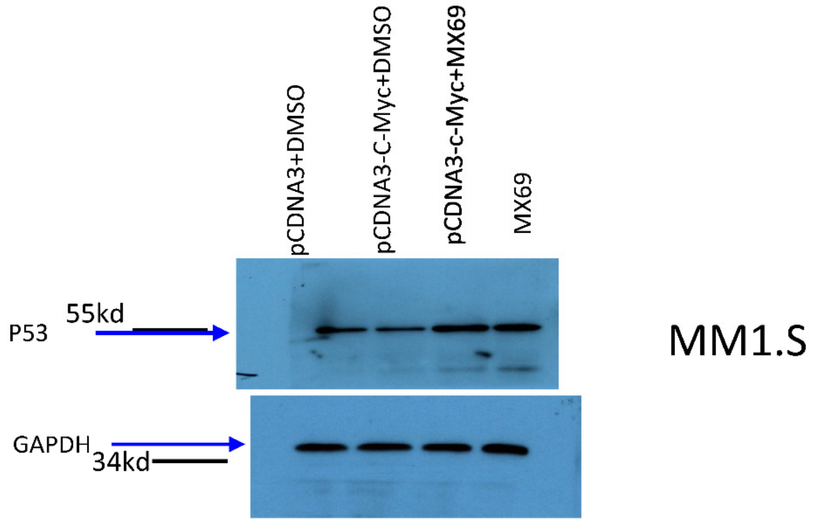

P53 proteins Intensity ratio (GAPDH was used as normalization standard) were calculated respective transfect with MX69.

	pCDNA3+DMSO	pCDNA3-c-Myc+DMSO	pCDNA3-c-Myc+MX69	MX69
P53	0.45	0.41	0.92	1

Figure S23. Uncropped blots for Figure S7C.

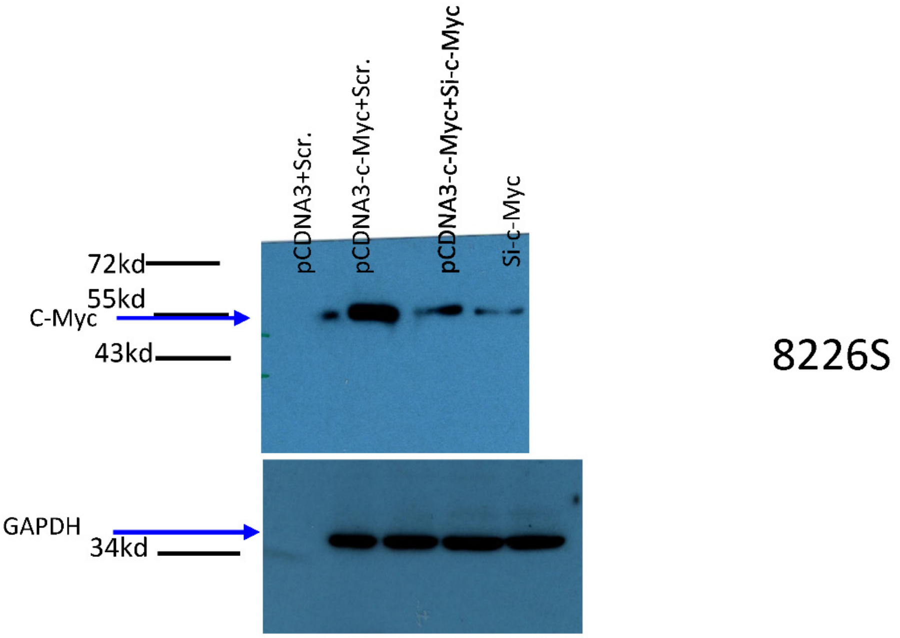

c-Myc proteins Intensity ratio (GAPDH was used as normalization standard) were calculated respective transfect with pCDNA3+Scr.

	pCDNA3+Scr.	pCDNA3-c-Myc+Scr.	pCDNA3-c-Myc+Si-c-Myc	Si-c-Myc
c-Myc	1	11.7	2.1	1.6

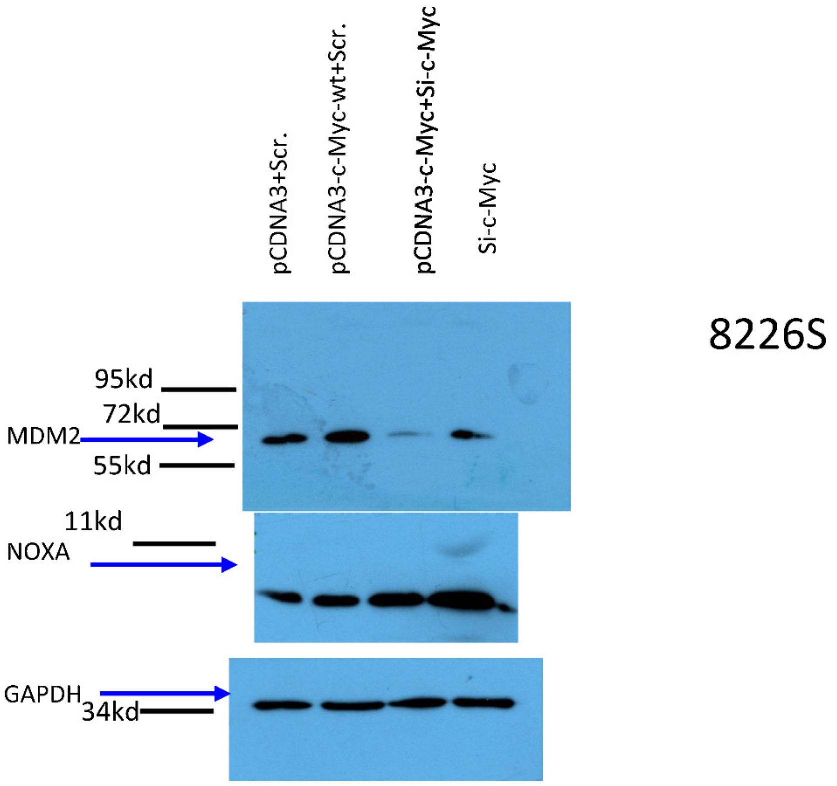

MDM2 and NOXA proteins Intensity ratio (GAPDH was used as normalization standard) were calculated respective transfect with pCDNA3+Scr and si-c-Myc.

	pCDNA3+Scr.	pCDNA3-c-Myc+Scr.	pCDNA3-c-Myc+si-c-Myc	Si-c-Myc
MDM2	1	3.2	0.45	0.6
NOXA	0.45	0.55	6.8	1

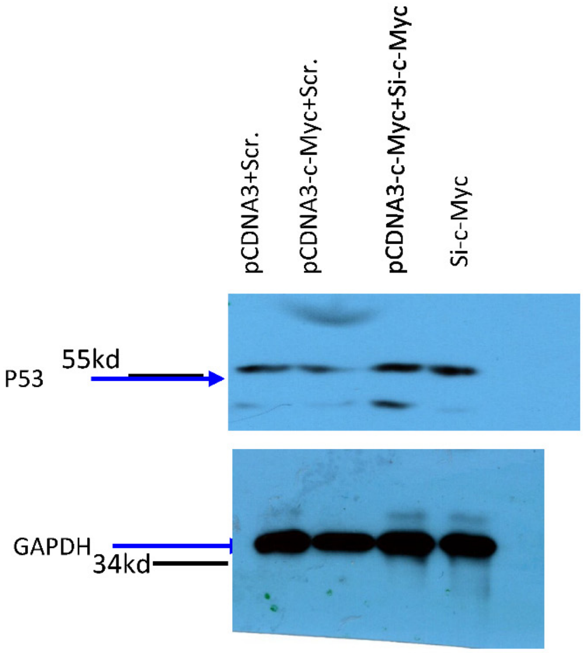

P53 proteins Intensity ratio (GAPDH was used as normalization standard) were calculated respective transfect with si-c-Myc.

	pCDNA3+Scr.	pCDNA3-c-Myc+Scr.	pCDNA3-c-Myc+ Si-c-Myc	Si-c-Myc
P53	0.39	0.35	0.88	1

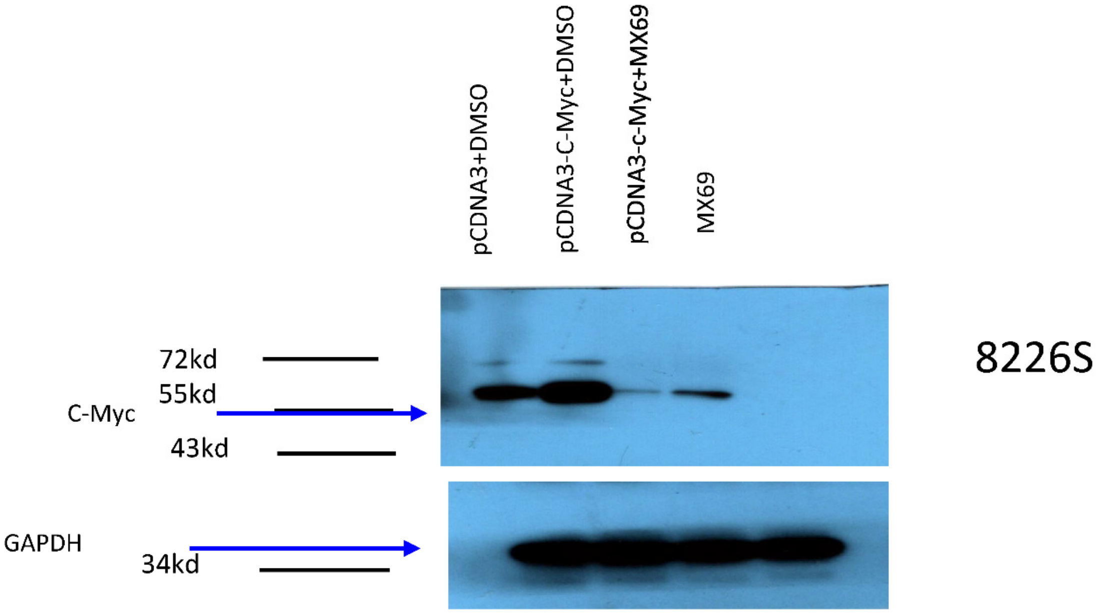

c-Myc proteins Intensity ratio (GAPDH was used as normalization standard) were calculated respective transfect with pCDNA3+DMSO

	pCDNA3+DMSO	pCDNA3-c-Myc+DMSO	pCDNA3-c-Myc+MX69	MX69
c-Myc	1	4.44	0.4	0.51

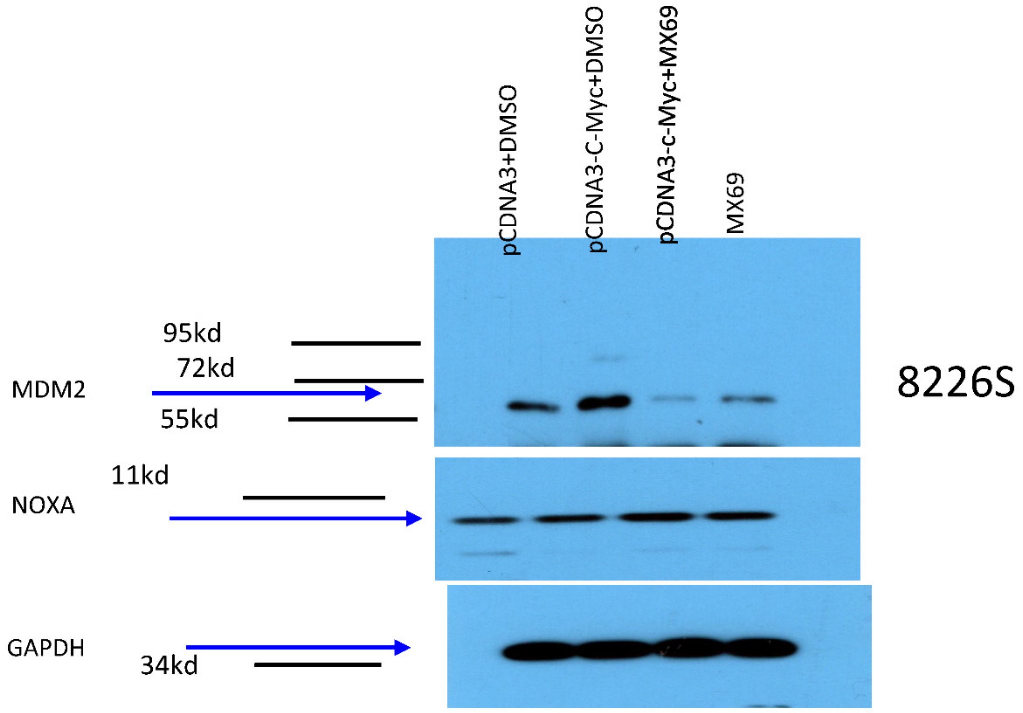

MDM2 and NOXA proteins Intensity ratio (GAPDH was used as normalization standard) were calculated respective transfect with pCDNA3+DMSO and MX69.

	pCDNA3+DMSO	pCDNA3-c-Myc+DMSO	pCDNA3-c-Myc+MX69	MX69
MDM2	1	3.1	0.43	0.53
NOXA	0.46	0.55	0.87	1

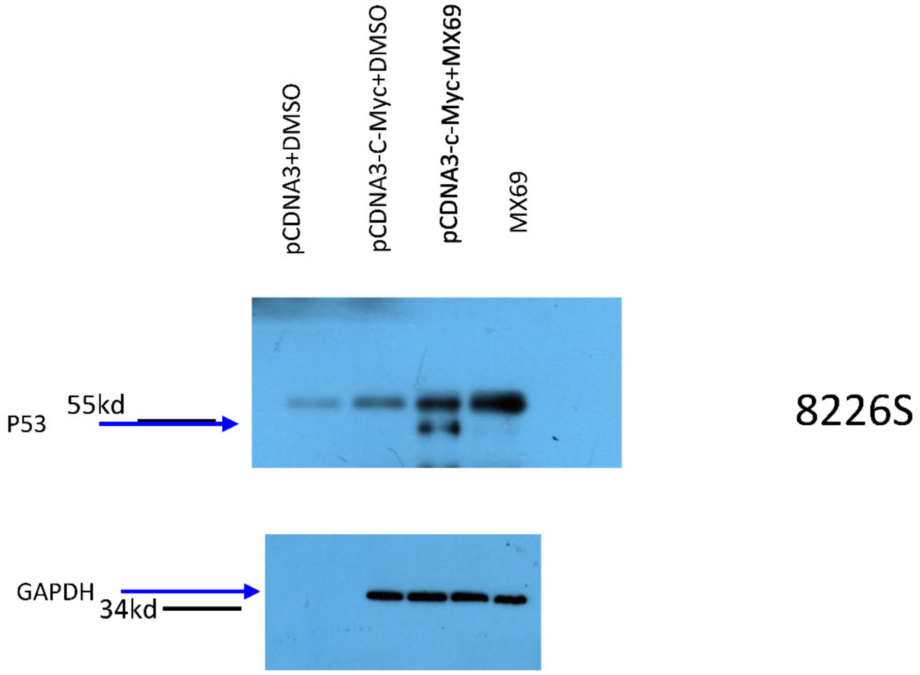

P53 proteins Intensity ratio (GAPDH was used as normalization standard) were calculated respective transfect with MX69.

	pCDNA3+DMSO	pCDNA3-c-Myc+DMSO	pCDNA3-c-Myc+MX69	MX69
P53	0.22	0.36	0.88	1

Figure S24. Uncropped blots for Figure S7D.

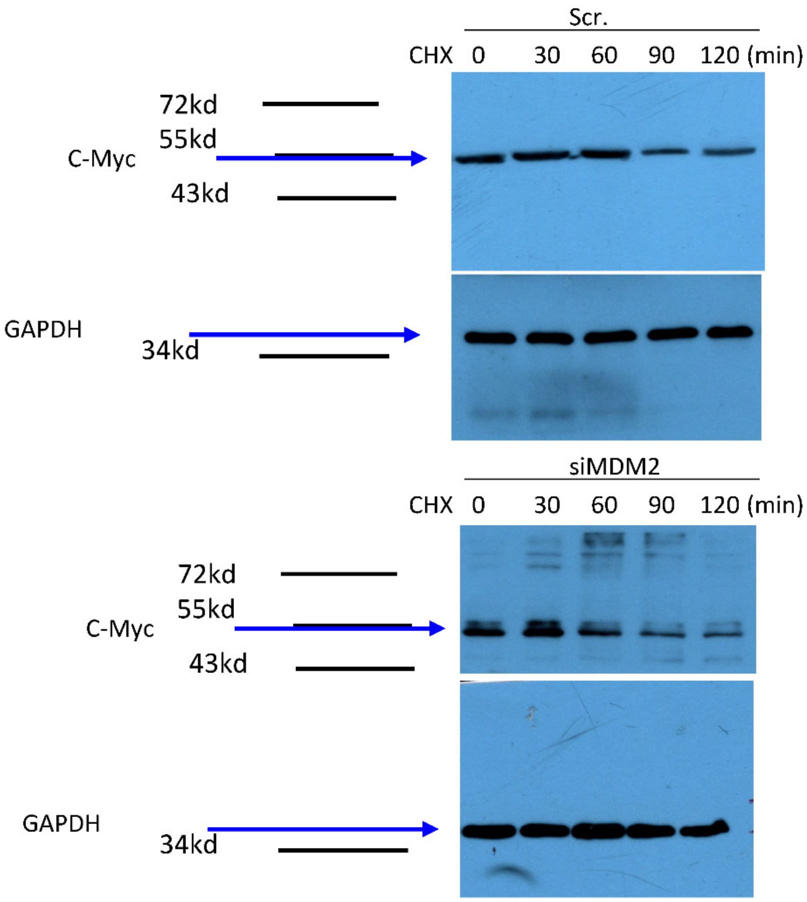

MDM2 protein Intensity ratio (GAPDH was used as normalization standard) was calculated respective treatment with zero minute treatment.

CHX										
	Scr.					siMDM2				
Min.	0	30	60	90	120	0	30	60	90	120
c-Myc	1	1.05	1.1	0.46	0.44	1	1.1	0.43	0.33	0.24

Figure S25. Uncropped blots for Figure S8D.
